# Classification and Analysis of Regulatory Pathways Using Graph Property, Biochemical and Physicochemical Property, and Functional Property

**DOI:** 10.1371/journal.pone.0025297

**Published:** 2011-09-28

**Authors:** Tao Huang, Lei Chen, Yu-Dong Cai, Kuo-Chen Chou

**Affiliations:** 1 Institute of Systems Biology, Shanghai University, Shanghai, People's Republic of China; 2 Key Laboratory of Systems Biology, Shanghai Institutes for Biological Sciences, Chinese Academy of Sciences, Shanghai, People's Republic of China; 3 Shanghai Center for Bioinformation Technology, Shanghai, People's Republic of China; 4 College of Information Engineering, Shanghai Maritime University, Shanghai, People's Republic of China; 5 Gordon Life Science Institute, San Diego, California, United States of America; National University of Ireland Galway, Ireland

## Abstract

Given a regulatory pathway system consisting of a set of proteins, can we predict which pathway class it belongs to? Such a problem is closely related to the biological function of the pathway in cells and hence is quite fundamental and essential in systems biology and proteomics. This is also an extremely difficult and challenging problem due to its complexity. To address this problem, a novel approach was developed that can be used to predict query pathways among the following six functional categories: (i) “Metabolism”, (ii) “Genetic Information Processing”, (iii) “Environmental Information Processing”, (iv) “Cellular Processes”, (v) “Organismal Systems”, and (vi) “Human Diseases”. The prediction method was established trough the following procedures: (i) according to the general form of pseudo amino acid composition (PseAAC), each of the pathways concerned is formulated as a 5570-D (dimensional) vector; (ii) each of components in the 5570-D vector was derived by a series of feature extractions from the pathway system according to its graphic property, biochemical and physicochemical property, as well as functional property; (iii) the minimum redundancy maximum relevance (mRMR) method was adopted to operate the prediction. A cross-validation by the jackknife test on a benchmark dataset consisting of 146 regulatory pathways indicated that an overall success rate of 78.8% was achieved by our method in identifying query pathways among the above six classes, indicating the outcome is quite promising and encouraging. To the best of our knowledge, the current study represents the first effort in attempting to identity the type of a pathway system or its biological function. It is anticipated that our report may stimulate a series of follow-up investigations in this new and challenging area.

## Introduction

During the past decade, much information on different organisms has been accumulated at both the genetic and metabolic levels; meanwhile, many specific databases, such as KEGG/LIGAND [1], [2], [3], [4], ENZYME [5], BRENDA [6], EcoCyc and MetaCyc [7], [8], have been developed. However, biological meaningful pathways, such as the regulatory pathway and metabolic pathway, are still poorly understood. As one of the most important pathways in systems biology, the regulatory pathway includes two kinds of interactions: direct protein–protein interactions (such as physical binding and phosphorylation) and indirect protein–protein interactions (such as the relations between transcription factors and downstream gene products) [2].

KEGG (Kyoto Encyclopedia of Genes and Genomes) [1], [2], [3], [4] is a collection of online databases for dealing with genomes, enzymatic pathways, and biological chemicals. KEGG contains five main databases [4]: (i) KEGG Atlas, (ii) KEGG Pathway, (iii) KEGG Genes, (iv) KEGG Ligand, and (v) KEGG BRITE. The KEGG BRITE database (http://www.genome.jp/kegg/brite.html) includes some known regulatory pathways. It is an ontology database for representing functional hierarchies of various biological objects. The database also includes molecules, cells, organisms, diseases and drugs, as well as the relationships among them [9], [10]. In this database, experimental knowledge is collected and diagramed as pathways, i.e. smaller networks of specific function. Several visualization tools have been developed to view and analyze the global networks through web interfaces [11], [12], [13].

According to the data in KEGG BRITE, regulatory pathways are classified into six pathway classes. Since different class pathway represents different biological function, developing a successful classifier to identify the pathway class is very useful in system biology. Some efforts have been made in this regard. Dale et al. [14] tried to predict whether a metabolic pathway is present or absent in an organism. In our previous work [15], we developed a model to predict whether a regulatory pathway can be formed for a system consisting of certain number of different proteins. But predicting the biological function of regulatory pathway is still an untouched problem. It is a big challenge in both systems biology and proteomics because this kind of information is very hard to recover and transform into the data that can be processed by computers. The purpose of this study is not to achieve a high accuracy, but to analyze some features, which may provide useful information for characterizing a meaningful regulatory pathway.

To realize this, some feature selection methods, such as the minimum redundancy maximum relevance [16] and incremental feature selection approaches, were employed to analyze the relevant features, while Nearest Neighbor Algorithm (NNA) [17], [18], Sequential Minimal Optimization (SMO) [19], [20] and Bayesian network (BayesNet) [21] were used to classify the pathways. Finally, the jackknife cross-validation [22] was adopted to evaluate the prediction performance. As a result, 49 features were selected as the optimal features and the overall accuracy by using these features was 78.8%.

It was suggested by analyzing the optimized features that biochemical and physicochemical property and functional property are important to determine the biological function of each regulatory pathway. Although it represents the first work ever in predicting the classification of regulatory pathways and it is still quite preliminary, we believe that our exploration can stimulate a series of follow-up studies in this area important to both system biology and proteomics.

According to a recent review [23], to establish a really useful statistical predictor for a protein system, we need to consider the following procedures: (i) construct or select a valid benchmark dataset to train and test the predictor; (ii) formulate the protein samples with an effective mathematical expression that can truly reflect their intrinsic correlation with the attribute to be predicted; (iii) introduce or develop a powerful algorithm (or engine) to operate the prediction; (iv) properly perform cross-validation tests to objectively evaluate the anticipated accuracy of the predictor; (v) establish a user-friendly web-server for the predictor that is accessible to the public. Below, let us describe how to deal with these steps one by one.”

## Materials and Methods

### Benchmark dataset

We downloaded the human KGML (KEGG XML) files from KEGG FTP site (ftp://ftp.genome.jp/pub/kegg/xml) in April 2009. We reduced the original data by the following two steps: (i) remove proteins without GO information or biochemical and physicochemical properties in each pathway; (ii) exclude pathways with less than three proteins. As a result, 146 regulatory pathways were obtained. According to the data in KEGG BRITE (http://www.genome.jp/kegg/brite.html), these pathways belong to the following six functional categories: (i) Metabolism, (ii) Genetic Information Processing, (iii) Environmental Information Processing, (iv) Cellular Processes, (v) Organismal Systems, and (vi) Human Diseases. Shown in **Table 1** is the distribution of the six classes of regulatory pathways in this study.

**Table 1:** The distribution of the 146 regulatory pathways.

Pathway class	Number of pathway
Metabolism	73
Genetic Information Processing	2
Environmental Information Processing	15
Cellular Processes	9
Organismal Systems	19
Human Diseases	28
Total	146

### Features construction

To develop a powerful predictor for classifying a protein system or pathway consisting of a set of proteins, one of the keys is to formulate the protein system with an effective mathematical expression that can truly reflect its intrinsic correlation with the attribute to be predicted [23]. In this regard, we can utilize the concept of pseudo amino acid composition (PseAAC) [24]. For a brief introduction about Chou's PseAAC, visit the Wikipedia web-page at http://en.wikipedia.org/wiki/Pseudo_amino_acid_composition. Ever since the concept of PseAAC was introduced, it has been widely used to study various problems in proteins and protein-related systems (see, e.g., [25], [26], [27], [28], [29], [30], [31], [32], [33], [34]). For various different modes of PseAAC, see [35]. Actually, the general form of PseAAC can be formulated as (see Eq.6 of [23]):
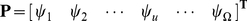
(1)where 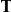 is a transpose operator, while the subscript 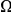 is an integer and its value as well as the components 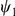, 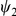, … will depend on how to extract the desired information from the amino acid sequence of 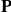. Likewise, a pathway 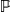 consisting a set of proteins can also be generally formulated as vector with 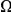 components; i.e.,
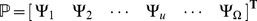
(2)where 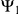 represents the 1^st^ feature of the pathway, 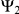 the 2^nd^ feature, and so forth. Below, let us elaborate how to define 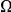 as well as the components in Eq.2.

#### 1. Graph property

Graphic approaches are deemed as useful tools to study complex biological systems as they can provide intuitive insights and the overall structure property, as indicated by various studies on a series of important biological topics [36], [37], [38], [39], [40], [41], [42], [43], [44], [45], [46], [47], [48]. To use the graphic approach for the current study, each regulatory pathway was represented as a graph, where the vertices represent proteins and the arcs represent the relations between the corresponding proteins. In fact, it is a directed graph or digraph [38], [39]. This is because the relation between two proteins is directional; i.e., one protein, say **P**_1_, can regulate another protein, say **P**_2_, while **P**_2_ cannot always regulate **P**_1_. In this paper, we extracted 88 graph features from each directed graph that represents a regulatory pathway. Most of the graph features were derived in [49], [50], [51], [52], [53] where, however, the graphs are undirected. In this study, we extended them into directed graphs. The features of our directed graphs can be briefed as follows.

1. Graph size and graph density. Let *G* = (*V*, *E*) be a pathway graph, where *V* denotes vertex set and *E* arcs set. The graph size is the number of vertices in the graph. |*E*|_max_ = |*V*|^2^ is the theoretical maximum number of arcs in *G* with |*V*| vertices. The graph density is calculated by |E|/|*E*|_max_ [49].
2. Degree statistics. The in-degree (out-degree) of a vertex is the number of its in-neighbors (out-neighbors). The mean, variance, median, and maximum of in-degree and out-degree, respectively, were taken as features in this feature group [50].
3. Edge weight statistics. Let *G* = (*V*, *w*(*E*)) be a weighted pathway graph where each arc is weighted by a weight *w* in the range of [0,1]. The symbol *e* is called a missing edge if *w*(*e*) = 0. In this study, the mean and variance of the arc weights were considered as features, including two different cases (with and without missing edges) [49].
4. Topological change. Let *G* = (*V*, *w*(*E*)) be a weighted pathway graph. This group of features is to measure the topological changes when different cutoffs of the weights are applied to the graph. The weight cutoffs included 0.1, 0.2, 0.3, 0.4, 0.5, 0.6, 0.7 and 0.8. Topology changes were defined as the change rate of the number of arcs in subgraphs under two consecutive cutoffs.
5. Degree correlation. Let *G* = (*V*, *E*) be a pathway graph with *V* = {*u*_1_,*u*_2_,…,*u_n_*}. For each vertex *u_i_*, calculate the average number of arcs of its in-neighbors and out-neighbors, respectively. Considered as features in this study were the mean, variance and maximum of the two kinds of property, respectively [51].
6. Clustering. Let *G* = (*V*, *E*) be a pathway graph with *V* = {*u*_1_,*u*_2_,…,*u_n_*}. For each vertex *u_i_*, calculate the graph density of the subgraph induced by its in-neighbors and out-neighbors, respectively. Take the mean, variance and maximum of the two kinds of property [50], respectively, as the features for the current study.
7. Topological. Let *G* = (*V*, *E*) be a pathway graph with *V* = {*u*_1_,*u*_2_,…,*u_n_*}. Define four function as follows: (i) *in-in*(*u_i_*, *u_j_*) for the number of both in-neighbors of *u_i_* and in-neighbors of *u_j_*; (ii) *in-out*(*u_i_*, *u_j_*) for the number of both in-neighbors of *u_i_* and out-neighbors of *u_j_*; (iii) *out-in*(*u_i_*, *u_j_*) for the number of both out-neighbors of *u_i_* and in-neighbors of *u_j_*; (iv) *out-out*(*u_i_*, *u_j_*) for the number of both out-neighbors of *u_i_* and out-neighbors of *u_j_*. For each vertex *u_i_*, calculate the four values *T_i_*_1_, *T_i_*_2_, *T_i_*_3_, and *T_i_*_4_ as follows: (i) *T_i_*_1_ is the mean of *in-in*(*u_i_*, *u_j_*)/*n_i_*_1_; (ii) *T_i_*_2_ the mean of *in-out*(*u_i_*, *u_j_*)/*n_i_*_1_; (iii) *T_i_*_3_ the mean of *out-in*(*u_i_*, *u_j_*)/*n_i_*_2_; (iv) *T_i_*_4_ the mean of *out-out*(*u_i_*, *u_j_*)/*n_i_*_1_. In the above, *n_i_*_1_ and *n_i_*_2_ are the number of in-neighbors and out-neighbors of *u_i_*, respectively. Take the mean, variance and maximum of *T_i_*_1_, *T_i_*_2_, *T_i_*_3_, and *T_i_*_4_, respectively, as the features [51] for the current study.
8. Singular values. Let *A* be the adjacent matrix of the pathway graph. Take the first three largest singular values [49] as the features for this study.
9. Local density change. Let *G* = (*V*, *E*) be a pathway graph with *V* = {*u*_1_,*u*_2_,…,*u_n_*}. For each vertex *u_i_*, let 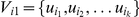 and 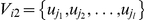 be its in-neighbors and out-neighbors, respectively. Here we only introduce how to extract features from out-neighbors of each vertex under the cutoff *w*, which may be 0, 0.1, 0.2, 0.3, 0.4, 0.5, 0.6, 0.7, 0.8 and 0.9. Construct a weighted undirected complete graph *K_i_* with vertices 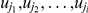 and the weights of each edge can be calculated by Eq. 2 in Section 2 “Gene ontology”. Extract a spanning subgraph *G_i_*(*w*) of *K_i_* with edges whose weights are greater than *w*. Calculate *L_i_*(*w*) = 2|*E*(*G_i_*(*w*))|/(*l*(*l*−1)) (*L_i_*(*w*) = 0 if *l*≤1). Take the mean and maximum of *L*_1_(*w*), *L*_2_(*w*),…, *L_n_*(*w*) under cutoff *w* as the features for the current study.

#### 2. Gene ontology

As mentioned before, some features need the arc weight to evaluate the relation between two proteins. Thus, we used the information from gene ontology consortium (GO) [54] to represent each of the proteins concerned and evaluate its relation with the other proteins. “Ontology” is a specification of a conceptualization and refers to the subject of existence. GO is established according to the following three criteria: molecular function, biological process, and cellular component. Using GO information to represent protein samples can catch their core features [23] as proved by significantly enhancing the success rate in predicting their subcellular localization [55], [56], [57]. The GO approach has also been used to study protein-protein interactions [58], [59]. Here, using the similar method as in [52], each protein sample can be formulated as a 5218-D vector:
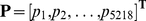
(3)where *p_i_* = 1 if the sample hit the 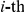 GO number; otherwise, *p_i_* = 0. The interaction between **P** *_i_* and **P** *_j_*, *i.e.* the weight of arc between the two proteins, is defined by
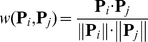
(4)where 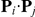 is the dot product of **P** *_i_* and **P** *_j_*, and ∥ **P** *_i_* ∥ and ∥ **P** *_j_* ∥ are their modulus.

#### 3. Biochemical and physicochemical property

Beside the graph property, the biological property of each pathway is also indispensable to characterize meaningful regulatory pathways. In this study, the biochemical and physicochemical properties, which have been used to study various biological problems [60], [61], [62], were employed to represent the biological property of each pathway. These properties included hydrophobicity, normalized van der Waals volume, polarity, polarizability, secondary structure, solvent accessibility, and amino acid compositions. For a regulatory pathway involving *n* proteins, both the mean and maximum values of their biological properties were taken for the features of the pathway, as detailed below.

1. Hydrophobicity, normalized van der Waals volume, polarity and polarizability: 42 features can be extracted from each of these properties [63], [64], respectively. Here we only describe how to obtain the features from the hydrophobicity property, while features from the other properties can be obtained in a similar way. Each amino acid is substituted by one of the three letters, polar (P), neutral (N) and hydrophobic (H). Given a protein sequence, use P, N or H to substitute each amino acid in the sequence, and the sequence thus obtained is called a protein pseudo-sequence. Composition (C) is the percentage of P, N and H in the whole pseudo-sequence. Transition (T) is the changing frequency between any two characters. Distribution (D) is the sequence segment (in percentage) of the pseudo-sequence which is needed to contain the first, 25%, 50%, 75% and the last of the Ps, Ns and Hs, respectively. In conclusion, there are three, three, and fifteen properties for (C), (T) and (D), respectively. Accordingly, we have 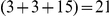 features for the “mean” category, 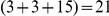 feature for the “maximum” category, and hence a total of 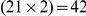 features by considering the “hydrophobicity” property alone. Similarly, we also have 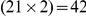 features by considering each of the other three properties, i.e., the “normalized van der Waals volume”, “polarity”, and “polarizability”. Thus, we have a total of 42×4 = 168 features by considering the above four properties.
2. Secondary structure: according to the secondary structural propensity of amino acids, each protein sequence can also be coded with three letters [65], [66]. Thus, like the case in considering hydrophobicity, we also have 21×2 = 42 features by considering the “secondary structure” property (or propensity).
3. Solvent accessibility: ACCpro [67] can be used to predict each amino acid as hidden (H) or exposed (E) to solvent. Then the protein sequence is coded with letters H and E. Use composition (C) for H, transition (T) between H and E, and five distributions (D) for H in this property. Thus we have (1+1+5)×2 = 14 features by considering the “solvent accessibility” property.
4. Amino acid compositions: it contains 20 components with each representing the percentage of each amino acid in a protein sequence [68]. Thus, we have 20 features for the “mean” category, and 20 features for the “maximum” category. Totally, we have 20×2 = 40 features for a pathway system by considering the amino acid composition.

Shown in **Table 2** is a breakdown of the 264 features for a pathway system by considering its biochemical and physicochemical properties. Before taking the mean and maximum values of each property into account, the following equations were used to adjust them according to a standard scale [61]:
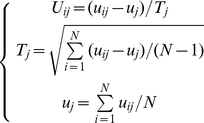
(5)where *T_j_* is the standard deviation of the *j*-th feature and *u_j_* the mean value of the *j*-th feature.

**Table 2:** A breakdown of the 264 features for a pathway system by considering its biochemical and physicochemical properties.

Properties	C	T	D	Mean category	Maximum category	Pathway system
Hydrophobicity	3	3	15	21	21	42
Normalized van der Waals volume	3	3	15	21	21	42
Polarity	3	3	15	21	21	42
Polarizability	3	3	15	21	21	42
Secondary structure	3	3	15	21	21	42
Solvent accessibility	1	1	5	7	7	14
Amino acid composition	20	N/A	N/A	20	20	40
Total	36	36	80	132	132	**264**

#### 4. Functional property

The last category of features is about the functional property of each regulatory pathway. The gene ontology enrichment score of pathway *i* on gene ontology item *j* was defined as the −log_10_ of the hypergeometric test p value [15], [69], [70], [71] of proteins in pathway *i* and can be computed by the following equation:
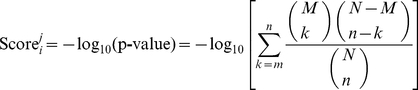
(6)where *N* is the number of overall proteins in KEGG of human, *M* is the number of proteins annotated to gene ontology item *j*, 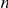 is the number of proteins in pathway *i*, 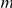 is the number of proteins in pathway *i* that are annotated to gene ontology item *j*. The larger the enrichment score of one gene ontology item, the more overrepresented this item is. There were a total of 5,218 gene ontology (GO) enrichment score features.

#### 5. Representation of each pathway

It follows from the description in Section 1 “Graph property”, 3 “Biochemical and physicochemical property” and 4 “Functional property” that the total number of features was 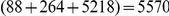, as summarized **Table 3**. Thus, according to Eq.2, each of the 146 pathway samples in the benchmark dataset (Table S1) will be represented by a 5570-D vector.

**Table 3:** A breakdown of the of 5570 features.

Categories	Group name	Number of features
Graph property	Graph size and graph density	2
	Degree statistics	8
	Edge weight statistics	4
	Topological change	7
	Degree correlation	6
	Clustering	6
	Topological	12
	Singular values	3
	Local density change	40
Biochemical and physicochemical property	Amino acid compositions	40
	Hydrophobicity, normalized van der Waals volume, polarity and polarizability	168
	Solvent accessibility	14
	Secondary structure	42
Functional property	Gene ontology enrichment score	5218
Total	N/A	5570

### mRMR method

Minimum Redundancy Maximum Relevance (mRMR), first proposed by Peng *et al.* [16], was employed in this study, as it is established according to two excellent criteria: Max-Relevance and Min-Redundancy. Max-Relevance guarantees that features giving most contribution to the classification will be selected, while Min-Redundancy guarantees that features whose classification ability has already been covered by selected features will be excluded. By mRMR program, we can obtain two feature lists: MaxRel features list and mRMR features list. MaxRel features list sort features only according to the Max-Relevance criteria, while mRMR features list is obtained in terms of both Max-Relevance and Min-Redundancy. Thus, for a feature set **Ω** with *N* features, mRMR program will execute *N* rounds and a feature with maximum relevance and minimum redundancy is selected in each round. Finally, we can obtain an ordered feature list, i.e., mRMR features list:
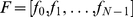
(7)For detail description of the mRMR method, please refer to Peng *et al.*'s paper [16]. Now, mRMR method has been widely utilized to tackle various biological problems [45], [52], [72], [73], [74], [75], [76] and deemed as a powerful and useful tool to extract important information in complex systems. The mRMR program developed by Peng et al [16] is available at http://penglab.janelia.org/proj/mRMR/.

### Prediction model

In this study, we tried three prediction methods: Nearest Neighbor Algorithm (NNA), Sequential Minimal Optimization (SMO) and Bayesian network (BayesNet). NNA using cosine similarity as “nearness” [15], [61], [62], [71], [77] was implemented with in-house script. The NNA program can be downloaded from http://pcal.biosino.org/NNA.html. SMO and BayesNet were implemented in Weka (Waikato Environment for Knowledge Analysis) [78]. Weka, which was developed by the University of Waikato in New Zealand, is software collecting a variety of state-of-art machine learning algorithms and data preprocessing tools. It provides extensive support for the whole process of experimental data mining, including preparing the input data, evaluating learning schemes statistically, and visualizing the input data and the result of learning [78]. Weka can be downloaded from http://www.cs.waikato.ac.nz/ml/weka/.

#### 1. Nearest Neighbor Algorithm (NNA)

Nearest Neighbor Algorithm (NNA) [17], [18], which has been widely used in bioinformatics and computational biology [15], [59], [60], [72], [79], [80], was adopted to predict the pathway class of each query pathway. The “nearness” is calculated as below
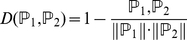
(8)where 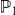 and 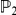 are two vectors representing two pathways, 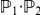 is their dot product, 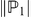 and 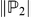 are the modulus of vector 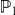 and 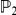. The smaller the 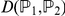, the more similar the two pathways are [55]. In NNA, suppose there are *m* training pathways, each of them belongs to exact one pathway class, and a query pathway needs to be classified into one pathway class. The distances between each of the *m* training pathways and the query pathway can be calculated, and the nearest neighbor of the query pathway is found. If the nearest neighbor belongs to the *i*-th pathway class, the query pathway is classified into the *i*-th pathway class. For an intuitive illustration of how NNA works, see Fig.5 of [23].

#### 2. SMO

SMO implements John Platt's sequential minimal optimization algorithm for training a support vector classifier using polynomial or Gaussian kernels [19], [20]. All attributes are processed before using SMO to make prediction, for example nominal attributes are transformed into binary ones, and attributes are normalized [78].

#### 3. BayesNet

BayesNet learns Bayesian networks under the assumptions that all attributes should be nominal (In particular, numeric ones should be prediscretized) and there are no missing values. Two different algorithms are used to estimate the conditional probability tables of the network [78] and several search algorithms are implemented for local score metrics, such as K2 [81], Hill Climbing [82], TAN [83], [84] and so on. For more detailed description of this classifiers in Weka can be found in [21].

### Jackknife test to examine the quality of the current prediction method

In statistical prediction, the following three cross-validation methods are often used to examine a predictor for its effectiveness in practical application: independent dataset test, subsampling test, and jackknife test [85]. However, of the three test methods, the jackknife test is deemed the most objective [56]. The reasons are as follows. **(i)** For the independent dataset test, although all the proteins used to test the predictor are outside the training dataset used to train it so as to exclude the “memory” effect or bias, the way of how to select the independent proteins to test the predictor could be quite arbitrary unless the number of independent proteins is sufficiently large. This kind of arbitrariness might result in completely different conclusions. For instance, a predictor achieving a higher success rate than the other predictor for a given independent testing dataset might fail to keep so when tested by another independent testing dataset [85]. **(ii)** For the subsampling test, the concrete procedure usually used in literatures is the 5-fold, 7-fold or 10-fold cross-validation. The problem with this kind of subsampling test is that the number of possible selections in dividing a benchmark dataset is an astronomical figure even for a very simple dataset, as demonstrated by Eqs.28–30 in [23]. Therefore, in any actual subsampling cross-validation tests, only an extremely small fraction of the possible selections are taken into account. Since different selections will always lead to different results even for a same benchmark dataset and a same predictor, the subsampling test cannot avoid the arbitrariness either. A test method unable to yield a unique outcome cannot be deemed as a good one. **(iii)** In the jackknife test, all the proteins in the benchmark dataset will be singled out one-by-one and tested by the predictor trained by the remaining protein samples. During the process of jackknifing, both the training dataset and testing dataset are actually open, and each protein sample will be in turn moved between the two. The jackknife test can exclude the “memory” effect. Also, the arbitrariness problem as mentioned above for the independent dataset test and subsampling test can be avoided because the outcome obtained by the jackknife cross-validation is always unique for a given benchmark dataset. Accordingly, the jackknife test has been increasingly and widely used by those investigators with strong math background to examine the quality of various predictors (see, e.g., [25], [26], [27], [28], [29], [30], [31], [32], [33], [34], [86], [87], [88], [89], [90]). In view of this, here the jackknife test was also used to examine the quality of the current predictor in identifying the pathway class.

### Incremental feature selection (IFS)

As described in Section “mRMR method”, mRMR features list *F* = [*f*_0_, *f*_1_,…,*f_N−_*_1_] can be obtained by mRMR program. Denote the *i*-th feature set by *F_i_* = { *f*_0_, *f*_1_,…,*f_i_*} (0≤*i*≤*N*−1). For each *i* (0≤*i*≤*N*−1), execute NNA, SMO and BayesNet with the features in *F_i_*, then the overall accuracy of the classification (ACC), defined by “the number of correctly predicted pathways”/“the total number of pathways”, evaluated by jackknife test, was obtained. As a result, we can plot a curve named IFS curve with *ACC* as its *y*-axis and the index *i* of *F_i_* as its *x*-axis.

## Results and Discussion

### Results of mRMR

The mRMR program was achieved from http://penglab.janelia.org/proj/mRMR. It was run with default parameters and two feature lists were obtained by executing mRMR program: (i) MaxRel features list; (ii) mRMR features list (see Table S2).

MaxRel features list was obtained by sorting features according to their contribution to the classification. We investigated the most relevant 1% of the features (totally 55) and **Table 4** shows the distribution of these features. It is clear that 32 (32/55, 58.18%) features come from biochemical and physicochemical property and 23 (23/55, 41.82%) features come from functional property. All of these indicate that among the adopted features the biochemical and physicochemical property of each pathway provide the most contribution to classification and functional property also gives important contribution. It is startling that none of the features about graph property was the most relevant 1% feature, while they were considered as important factors to form some biological meaningful systems, such as protein complex [45], [53]. In this study, we only take care of classifying a regulatory pathway into correct pathway class but not to analyze which feature is more important to form a regulatory pathway. In this stage, graph property may be not very important while biological and functional properties are more important to determine the biological function of each pathway.

**Table 4:** The distribution of the most relevant 55 features.

Category	Number of features
Graph property	0
Biochemical and physicochemical property	32
Functional property	23
Total	55

### Results of IFS

**Figure 1:**
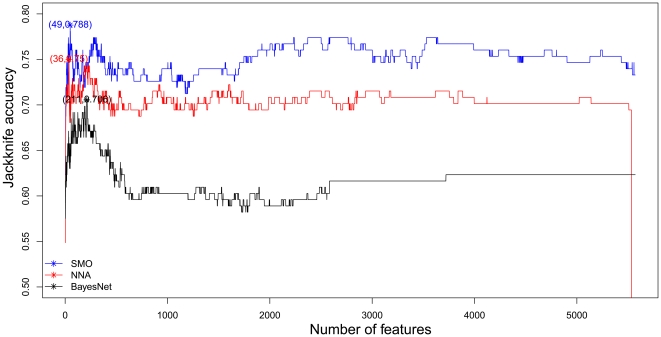
The IFS curve. The highest ACC value of IFS is 78.8% using 49 features and SMO model.

Shown in **Figure 1** are the IFS curves of NNA, SMO and BayesNet. The highest ACC value of IFS is 78.8% using 49 features and SMO models (See **Table 5** for the detail 49 features). The detailed IFS data can be found in Table S3.

**Table 5:** The 49 optimized features.

Order	Featurename
1	secondary_structure_composition_P_max
2	solvent_accessibility_composition_H_mean
3	solvent_accessibility_distribution_H.0.75_max
4	GO:0043627 response to estrogen stimulus
5	GO:0045121 membrane raft
6	secondary_structure_distribution_H.0.25_max
7	AA_composition_S_mean
8	secondary_structure_distribution_N.0.25_max
9	VanDerWaal_composition_P_max
10	GO:0043330 response to exogenous dsRNA
11	VanDerWaal_distribution_H.0.75_max
12	AA_composition_T_max
13	AA_composition_D_max
14	secondary_structure_distribution_H.0.5_max
15	GO:0048519 negative regulation of biological process
16	GO:0002687 positive regulation of leukocyte migration
17	secondary_structure_composition_P_mean
18	polarity_composition_N_max
19	GO:0042088 T-helper 1 type immune response
20	polarity_transition_NH_max
21	AA_composition_S_max
22	GO:0042063 gliogenesis
23	polarizability_distribution_P.0.75_max
24	GO:0090068 positive regulation of cell cycle process
25	GO:0014829 vascular smooth muscle contraction
26	secondary_structure_distribution_H.0.75_max
27	AA_composition_Q_mean
28	GO:0030225 macrophage differentiation
29	GO:0046661 male sex differentiation
30	hydrophobicity_composition_N_max
31	solvent_accessibility_distribution_H.0.0_max
32	polarity_distribution_P.0.5_max
33	polarizability_distribution_H.0.75_max
34	GO:0031594 neuromuscular junction
35	GO:0031330 negative regulation of cellular catabolic process
36	AA_composition_P_max
37	GO:0042953 lipoprotein transport
38	GO:0048523 negative regulation of cellular process
39	GO:0030217 T cell differentiation
40	GO:0007517 muscle organ development
41	GO:0009913 epidermal cell differentiation
42	GO:0042177 negative regulation of protein catabolic process
43	GO:0048641 regulation of skeletal muscle tissue development
44	hydrophobicity_distribution_N.0.75_max
45	hydrophobicity_distribution_H.0.75_max
46	GO:0022408 negative regulation of cell-cell adhesion
47	GO:0048608 reproductive structure development
48	GO:0045638 negative regulation of myeloid cell differentiation
49	GO:0006897 endocytosis

**Figure 2:**
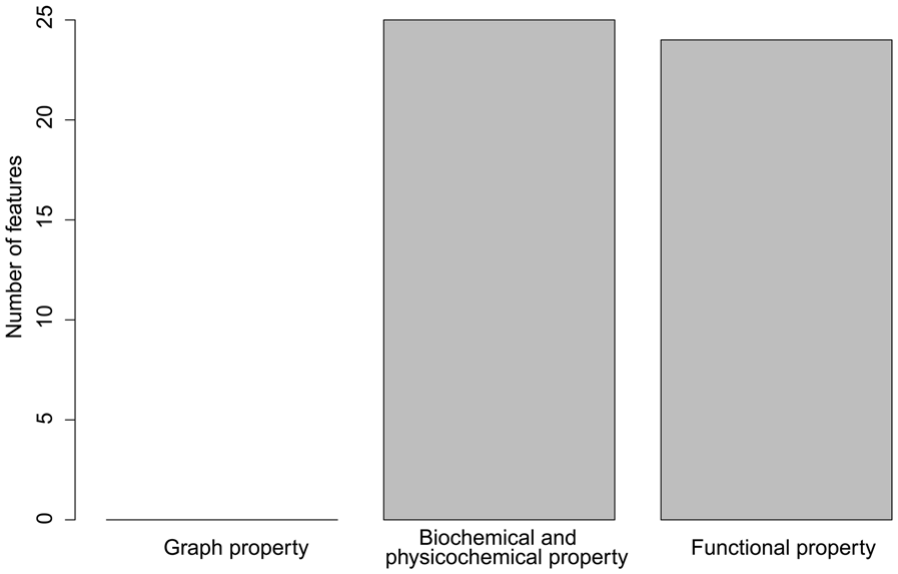
Distribution of the optimized 49 features. It is straightforward to see that 25 (25/49, 51.0%) features were from the biochemical and physicochemical property and 24 (24/49, 49.0%) features were from the functional property, while none of features in graph property was selected into the optimized feature set.

**Figure 2** shows the distribution of the optimized 49 features. It is straightforward to see that 25 (25/49, 51.0%) features were from the biochemical and physicochemical property and 24 (24/49, 49.0%) features were from the functional property, while none of features in graph property was selected into the optimized feature set. All of these indicate the same conclusion as described in Section “Results of mRMR”.

### Analysis of optimal features for pathway classification

It was seen from **Table 5** and **Figure 2** that the biochemical and physicochemical properties and Gene Ontology functional properties were important for pathway classification.

Within the selected 25 biochemical and physicochemical properties, there were 6 secondary structure features, 6 amino acid composition features, 3 solvent accessibility features, 3 polarity features, 3 hydrophobicity features, 2 vanderWaal features and 2 polarizability features. Obviously, secondary structure features and amino acid composition features were more important than other biochemical and physicochemical properties. The correct secondary structure of protein is essential to its function. Structural incorrect proteins are associated with many different kinds of disease such as Alzheimer's disease, Huntington's and Parkinson's disease [91]. In KEGG pathway classification, there are 28 disease pathways. Some of the disease pathways, such as neurodegenerative disease pathways and cancer pathways, are caused by or associated with protein misfolding [91]. Amino acid composition has been used to explain a lot of biological phenomenon, such as translation rate [62] and metabolic stability of proteins [61]. Amino acid composition has a close relationship with protein synthesis and degradation [62], [70]. In KEGG pathway classification, there are 73 metabolism pathways. The amino acid composition features may affect these metabolism pathways.

To investigate the association between KEGG pathway classes and GO terms in optimized features, we calculated their hypergeometric test p values which were shown in **Table 6**. As shown from the table, “Metabolism” pathways were associated with GO term “GO:0043627 response to estrogen stimulus”, “Genetic Information Processing” pathways were associated with GO term “GO:0045121 membrane raft”, “Environmental Information Processing” pathways, “Cellular Processes” pathways, “Organismal Systems” pathways and “Human Diseases” pathways were associated with many GO terms in optimized features. Some associations are obvious and well-known, such as the association between “Environmental Information Processing” pathways and GO term “GO:0043627 response to estrogen stimulus”, the association between “Cellular Processes” pathways and GO terms “GO:0048519 negative regulation of biological process” and “GO:0048523 negative regulation of cellular process”, the association between “Organismal Systems” pathways and GO terms “GO:0030217 T cell differentiation”, “GO:0030225 macrophage differentiation” etc., the association between “Human Diseases” pathways and GO terms “GO:0048519 negative regulation of biological process”, “GO:0048523 negative regulation of cellular process” and “GO:0042063 gliogenesis”. The relationship between “Metabolism” pathways and GO term “GO:0043627 response to estrogen stimulus” may be indirect. Estrogen can introduce dramatic changes of cell, such as apoptosis and carcinogenesis [92], [93]. During these cellular changes, the metabolism pathways will change as well. “Genetic Information Processing” pathways include many biological processes, such as transcription, translation, folding, sorting, degradation, replication and repair. All these steps require translocation of big molecular which needs the assistant of membrane systems. Membrane raft involves in biosynthetic traffic, endocytosis and signal transduction [94].

**Table 6:** Hypergeometric test of overlap between KEGG pathway classes and GO terms in optimized features.

	Metabolism	Genetic Information Processing	Environmental Information Processing	Cellular Processes	Organismal Systems	Human Diseases
GO:0043627 response to estrogen stimulus	0.032588	1	5.15E-16	1.86E-08	0.004826	2.30E-19
GO:0045121 membrane raft	0.681728	0.018851	2.68E-13	7.52E-15	1.09E-22	8.64E-15
GO:0043330 response to exogenous dsRNA	1	1	0.106165	0.003522	0.000117	0.001727
GO:0048519 negative regulation of biological process	1	1	1.86E-59	8.01E-39	4.20E-12	1.90E-51
GO:0002687 positive regulation of leukocyte migration	1	1	2.11E-09	0.001789	0.013702	0.000707
GO:0042088 T-helper 1 type immune response	1	1	3.50E-06	0.471266	0.094723	0.001178
GO:0042063 gliogenesis	0.993714	1	5.20E-11	1.30E-05	0.019525	1.32E-13
GO:0090068 positive regulation of cell cycle process	0.911776	1	9.12E-08	3.49E-06	0.024096	3.29E-08
GO:0014829 vascular smooth muscle contraction	1	1	0.000189	0.049965	0.023416	0.002415
GO:0030225 macrophage differentiation	1	1	0.003204	0.022913	0.00372	0.001178
GO:0046661 male sex differentiation	0.664515	1	4.00E-10	0.036323	0.938207	3.85E-07
GO:0031594 neuromuscular junction	1	1	0.001106	4.49E-06	1.97E-05	0.00224
GO:0031330 negative regulation of cellular catabolic process	1	1	0.006858	0.527536	0.137844	0.00224
GO:0042953 lipoprotein transport	1	1	0.127363	0.312566	0.023416	0.031663
GO:0048523 negative regulation of cellular process	0.999997	1	1.89E-56	1.93E-38	1.57E-08	4.91E-50
GO:0030217 T cell differentiation	0.957773	1	1.26E-16	0.023685	0.000397	1.82E-10
GO:0007517 muscle organ development	0.998366	1	6.32E-12	6.49E-09	0.32379	2.38E-09
GO:0009913 epidermal cell differentiation	1	1	0.123185	0.55964	0.968491	0.395449
GO:0042177 negative regulation of protein catabolic process	1	1	0.019214	0.002942	0.021538	0.001178
GO:0048641 regulation of skeletal muscle tissue development	1	1	5.03E-05	0.001284	0.447341	2.50E-06
GO:0022408 negative regulation of cell-cell adhesion	1	1	0.015685	0.040951	0.017213	0.001727
GO:0048608 reproductive structure development	0.431739	1	2.90E-16	0.036125	0.271969	4.81E-12
GO:0045638 negative regulation of myeloid cell differentiation	1	1	0.032936	0.289118	0.009817	1.09E-06
GO:0006897 endocytosis	0.995474	1	0.000121	0.012134	0.09916	0.006247

Combining the 25 biochemical and physicochemical properties and 24 Gene Ontology functional properties together, most KEGG pathways can correctly classified with reasonable biological meanings. The prediction model can be used to classify new pathway into existing pathway function groups. This means predicting the function of new pathways which is one of the ultimate goals of biology research.

We have analyzed 5570 features extracted from each of known regulatory pathway in humans. Of the 5570 features, 88 were derived from the graph property, 264 from the biochemical and physicochemical property of proteins, and 5218 from the functional property. Subsequently, the mRMR method and IFS techniques were employed to analyze and identify the the important features. Nearest neighbor algorithm and jackknife test were utilized to evaluate the accuracy of the classifier. As a result, 49 features were found to be as the important features for classifying the pathway groups according to their biological functions. These findings might provide useful insights, stimulating in-depth investigation into such an important and challenging problem.

## Supporting Information

Table S1 **The pathway benchmark dataset. It contains 146 pathways classified into six classes or groups according their biological functions.**
(XLS)

Table S2 **Two lists obtained by mRMR program.**
(PDF)

Table S3 **The IFS results for NNA, SMO and BayesNet.**
(XLS)
