# Supplementary material for: Classification and Analysis of Regulatory Pathways Using Graph Property, Biochemical and Physicochemical Property, and Functional Property
Source: PLoS One. 2011 Sep 28;6(9):e25297. doi: 10.1371/journal.pone.0025297 (PMC3182212; doi:10.1371/journal.pone.0025297)
Supplement: Table S2 — Two lists obtained by mRMR program. (PDF) [file pone.0025297.s002.pdf]

Table S2: Two lists obtained by mRMR program

(1) MaxRel features list

Order	Feature name
1	secondary_structure_composition_P_max
2	solvent_accessibility_composition_H_mean
3	solvent_accessibility_distribution_H.0.75_max
4	secondary_structure_distribution_H.0.25_max
5	AA_composition_S_max
6	secondary_structure_distribution_H.0.5_max
7	VanDerWaal_distribution_H.0.75_max
8	polarizability_distribution_H.0.75_max
9	secondary_structure_distribution_N.0.25_max
10	secondary_structure_distribution_H.0.75_max
11	polarity_composition_N_max
12	polarity_transition_NH_max
13	AA_composition_D_max
14	VanDerWaal_composition_P_max
15	hydrophobicity_composition_N_max
16	polarity_distribution_N.0.75_max
17	hydrophobicity_distribution_N.0.75_max
18	AA_composition_T_max
19	secondary_structure_distribution_N.0.5_max
20	polarity_distribution_P.0.5_max
21	polarizability_distribution_P.0.75_max
22	GO:0048519
23	GO:0043627
24	AA_composition_S_mean
25	AA_composition_P_max
26	hydrophobicity_distribution_H.0.75_max
27	hydrophobicity_transition_PN_max
28	secondary_structure_composition_P_mean
29	GO:0045121
30	GO:0048523
31	GO:0043125
32	GO:0022408
33	GO:0030857
34	secondary_structure_distribution_N.0.0_max
35	VanDerWaal_distribution_P.0.75_max
36	GO:0051057
37	GO:0046579
38	GO:0031330
39	GO:0043204
40	GO:0014829
41	GO:0030225

42	VanDerWaal_distribution_H.0.5_max
43	polarizability_distribution_H.0.5_max
44	polarity_composition_H_max
45	GO:0042063
46	GO:0048545
47	GO:0032320
48	GO:0005545
49	hydrophobicity_composition_P_max
50	GO:0016601
51	GO:0090068
52	GO:0048167
53	GO:0051385
54	GO:0051668
55	GO:0043330
56	GO:0045787
57	GO:0043524
58	secondary_structure_transition_PN_max
59	GO:0007517
60	GO:0034109
61	GO:0051353
62	GO:0009411
63	GO:0002687
64	AA_composition_Q_max
65	GO:0032228
66	GO:0048168
67	GO:0008542
68	GO:0007632
69	GO:0051932
70	GO:0030216
71	GO:0043559
72	GO:0042177
73	GO:0043547
74	solvent_accessibility_distribution_H.0.0_max
75	GO:0045737
76	GO:0048169
77	GO:0035020
78	GO:0007569
79	GO:0035022
80	GO:0006897
81	GO:0010324
82	GO:0009913
83	GO:0032891
84	GO:0045600
85	GO:0032890
86	AA_composition_V_mean
87	GO:0003056
88	secondary_structure_distribution_P.0.75_max

89	GO:0032026
90	GO:0050995
91	hydrophobicity_transition_NH_mean
92	GO:0031998
93	GO:0035014
94	GO:0010970
95	GO:0048641
96	GO:0070227
97	GO:0032839
98	GO:0031435
99	GO:0032838
100	AA_composition_Q_mean
101	GO:0032369
102	GO:0032770
103	GO:0040008
104	AA_composition_N_max
105	GO:0042692
106	GO:0050678
107	GO:0050673
108	GO:0043560
109	GO:0005515
110	solvent_accessibility_distribution_H.0.5_max
111	GO:0048009
112	GO:0043393
113	GO:0046661
114	GO:0042953
115	polarity_distribution_P.0.75_max
116	GO:0043069
117	GO:0060548
118	polarity_distribution_N.0.5_max
119	GO:0045137
120	GO:0005829
121	GO:0009991
122	GO:0006915
123	GO:0032355
124	polarity_distribution_N.0.0_max
125	GO:0030855
126	GO:0046982
127	GO:0007009
128	GO:0007568
129	GO:0007398
130	GO:0050930
131	GO:0015911
132	GO:0002028
133	GO:0046326
134	GO:0010828
135	GO:0032768

136	GO:0032000
137	GO:0050994
138	VanDerWaal_distribution_P.0.0_max
139	hydrophobicity_distribution_H.0.5_max
140	GO:0010001
141	AA_composition_C_max
142	GO:0032434
143	GO:0046578
144	polarizability_transition_PH_mean
145	GO:0047496
146	GO:0014706
147	GO:0045930
148	VanDerWaal_composition_N_max
149	GO:0060537
150	GO:0045765
151	GO:0007611
152	polarizability_distribution_P.0.5_max
153	GO:0031575
154	GO:0030217
155	GO:0051650
156	GO:0051648
157	GO:0042088
158	GO:0031594
159	GO:0071216
160	GO:0007548
161	GO:0003006
162	GO:0051716
163	GO:0051049
164	GO:0045767
165	GO:0051341
166	GO:0006468
167	GO:0048869
168	GO:0045725
169	GO:0070875
170	GO:0031328
171	GO:0051056
172	GO:0034620
173	GO:0030968
174	GO:0071445
175	GO:0031069
176	GO:0048608
177	solvent_accessibility_transition_HE_max
178	GO:0023051
179	GO:0009966
180	GO:0010033
181	GO:0003690
182	GO:0016773

183	GO:0009628
184	GO:0010165
185	GO:0045921
186	GO:0048708
187	GO:0007242
188	GO:0051492
189	VanDerWaal_distribution_P.0.5_max
190	GO:0043687
191	GO:0046329
192	GO:0070303
193	GO:0043409
194	GO:0010765
195	GO:0010748
196	GO:0010746
197	GO:0042698
198	GO:0033059
199	GO:0045638
200	GO:0040007
201	GO:0048732
202	polarity_transition_PN_mean
203	GO:0031434
204	GO:0030234
205	GO:0008219
206	GO:0016265
207	GO:0012501
208	GO:0014911
209	GO:0048730
210	GO:0009895
211	GO:0090150
212	GO:0090004
213	GO:0090002
214	GO:0050999
215	GO:0090003
216	GO:0048259
217	GO:0004722
218	GO:0080010
219	GO:0061061
220	GO:0009314
221	GO:0051000
222	GO:0010941
223	GO:0043067
224	GO:0019900
225	GO:0019899
226	GO:0044057
227	third_singular_values
228	GO:0042221
229	GO:0051051

230	GO:0045935
231	GO:0030154
232	GO:0051146
233	GO:0004672
234	GO:0045580
235	GO:0032879
236	GO:0045596
237	GO:0007612
238	GO:0070141
239	GO:0010596
240	GO:0022612
241	GO:0051272
242	GO:0023034
243	GO:0016044
244	GO:0061024
245	GO:0007098
246	GO:0051297
247	GO:0006796
248	GO:0006793
249	GO:0051173
250	GO:0043029
251	GO:0043066
252	AA_composition_M_max
253	GO:0045892
254	GO:0051253
255	GO:0042325
256	GO:0019220
257	GO:0051174
258	GO:0008022
259	GO:0045822
260	GO:0010212
261	GO:0030275
262	secondary_structure_distribution_H.0.0_max
263	GO:0022602
264	GO:0000122
265	GO:0017148
266	GO:0090066
267	GO:0048522
268	secondary_structure_distribution_P.0.5_max
269	GO:0051963
270	GO:0046777
271	GO:0001822
272	GO:0072001
273	GO:0010907
274	GO:0048856
275	GO:0044089
276	GO:0051962

277	GO:0051965
278	GO:0032436
279	GO:0070231
280	GO:0016605
281	GO:0044419
282	GO:0042981
283	GO:0001954
284	GO:0004674
285	hydrophobicity_distribution_H.0.25_max
286	GO:0048634
287	GO:0016202
288	GO:0001558
289	GO:0032231
290	GO:0014068
291	GO:0043523
292	GO:0051219
293	GO:0042633
294	GO:0001942
295	GO:0022405
296	GO:0022404
297	GO:0042303
298	GO:0010557
299	GO:0009891
300	GO:0045840
301	GO:0051785
302	GO:0005942
303	GO:0016303
304	GO:0046834
305	GO:0001727
306	GO:0046934
307	GO:0035004
308	GO:0046854
309	GO:0043583
310	polarizability_distribution_P.0.25_max
311	GO:0050807
312	GO:0006357
313	GO:0040017
314	GO:0009612
315	GO:0048589
316	GO:0007154
317	GO:0009416
318	GO:0048103
319	GO:0032535
320	GO:0032318
321	GO:0030511
322	secondary_structure_distribution_N.0.75_max
323	secondary_structure_distribution_P.0.25_max

324	GO:0019901
325	GO:0006469
326	GO:0009725
327	GO:0090183
328	GO:0090184
329	polarity_composition_P_mean
330	secondary_structure_composition_N_max
331	GO:0048747
332	GO:0043209
333	GO:0006983
334	GO:0032350
335	AA_composition_T_mean
336	GO:0045922
337	secondary_structure_transition_PH_mean
338	GO:0033077
339	GO:0051254
340	GO:0010608
341	GO:0019207
342	GO:0051270
343	GO:0007264
344	GO:0005488
345	GO:0007265
346	GO:0019902
347	GO:0019903
348	GO:0051093
349	GO:0048839
350	GO:0008406
351	GO:0010604
352	GO:0045428
353	GO:0004690
354	GO:0043550
355	GO:0045806
356	GO:0046825
357	GO:0051147
358	GO:0014070
359	GO:0051153
360	GO:0009890
361	GO:0080134
362	GO:0051897
363	GO:0048518
364	GO:0031659
365	GO:0031657
366	hydrophobicity_distribution_N.0.25_max
367	GO:0008361
368	GO:0051412
369	GO:0016049
370	GO:0051291

371	GO:0008286
372	GO:0005100
373	GO:0000186
374	GO:0045727
375	GO:0005901
376	GO:0009653
377	GO:0032502
378	GO:0048731
379	GO:0045619
380	GO:0043270
381	GO:0002762
382	GO:0060263
383	GO:0045429
384	solvent_accessibility_composition_H_max
385	GO:0045793
386	GO:0051348
387	polarity_transition_PH_max
388	GO:0019233
389	GO:0045944
390	GO:0045893
391	GO:0010628
392	GO:0045941
393	GO:0033673
394	GO:0045927
395	GO:0047485
396	GO:0021700
397	GO:0045741
398	GO:0042176
399	secondary_structure_composition_H_mean
400	GO:0050866
401	GO:0048660
402	GO:0048659
403	GO:0030307
404	GO:0019079
405	GO:0007270
406	GO:0007050
407	GO:0044445
408	AA_composition_P_mean
409	GO:0004697
410	GO:0014065
411	GO:0070887
412	GO:0022898
413	GO:0034762
414	GO:0016310
415	GO:0050679
416	GO:0031325
417	GO:0043149

418	GO:0030546
419	GO:0051128
420	GO:0009893
421	GO:0010149
422	GO:0014015
423	hydrophobicity_composition_H_mean
424	GO:0051129
425	GO:0048513
426	GO:0002763
427	GO:0042640
428	GO:0048143
429	GO:0048742
430	GO:0030125
431	GO:0014002
432	GO:0030120
433	GO:0030118
434	GO:0030132
435	GO:0043407
436	GO:0045913
437	GO:0010676
438	GO:0045216
439	GO:0010646
440	GO:0050926
441	GO:0006916
442	GO:0050927
443	out_out_topological_max
444	GO:0050853
445	GO:0048661
446	GO:0034976
447	GO:0046546
448	GO:0002039
449	GO:0044253
450	GO:0001667
451	GO:0045069
452	GO:0051271
453	GO:0051384
454	GO:0050681
455	GO:0008047
456	GO:0045736
457	GO:0042551
458	GO:0030235
459	GO:0048741
460	GO:0040014
461	GO:0006366
462	GO:0002360
463	GO:0001763
464	GO:0018105

465	GO:0050869
466	GO:0007093
467	GO:0007006
468	GO:0048592
469	GO:0043405
470	GO:0034613
471	GO:0070727
472	GO:0001541
473	GO:0044087
474	GO:0006814
475	GO:0021930
476	GO:0021534
477	GO:0021924
478	GO:0021782
479	GO:0046322
480	GO:0001933
481	GO:0010631
482	GO:0010634
483	GO:0090132
484	GO:0090130
485	GO:0010632
486	GO:0007005
487	GO:0000079
488	GO:0051130
489	GO:0006913
490	GO:0030168
491	GO:0051169
492	hydrophobicity_distribution_P.0.25_max
493	GO:0010332
494	GO:0050777
495	GO:0005905
496	GO:0016043
497	GO:0045687
498	GO:0055002
499	GO:0055001
500	GO:0030856
501	graph_size
502	GO:0009888
503	GO:0031960
504	GO:0070201
505	GO:0032880
506	GO:0051168
507	GO:0031646
508	GO:0051971
509	GO:0048538
510	GO:0010629
511	GO:0016481

512	GO:0048813
513	GO:0032368
514	GO:0003704
515	GO:0007492
516	GO:0032582
517	GO:0008544
518	GO:0009719
519	GO:0035116
520	GO:0016301
521	GO:0045646
522	GO:0045597
523	GO:0043406
524	GO:0014066
525	GO:0032412
526	GO:0034765
527	VanDerWaal_transition_NH_max
528	GO:0006984
529	GO:0051050
530	GO:0004715
531	GO:0014009
532	GO:0048754
533	GO:0008283
534	GO:0043198
535	GO:0007281
536	GO:0031327
537	GO:0032403
538	GO:0001889
539	GO:0061008
540	GO:0005979
541	GO:0070873
542	GO:0010962
543	GO:0032885
544	GO:0032881
545	GO:0042127
546	GO:0007406
547	GO:0035051
548	GO:0010638
549	GO:0045595
550	GO:0009887
551	GO:0004723
552	GO:0009617
553	polarity_distribution_P.0.25_max
554	GO:0045621
555	GO:0051496
556	GO:0032233
557	GO:0008285
558	GO:0045670

559	GO:0030097
560	GO:0050769
561	GO:0007635
562	GO:0032570
563	GO:0044459
564	GO:0040013
565	GO:0030336
566	GO:0004691
567	GO:0051783
568	GO:0007088
569	GO:0048705
570	GO:0010692
571	hydrophobicity_distribution_P.0.5_max
572	GO:0032496
573	GO:0055123
574	GO:0048546
575	GO:0004428
576	GO:0031647
577	GO:0045582
578	GO:0008287
579	GO:0010310
580	GO:0051969
581	GO:0033028
582	GO:0046324
583	GO:0048146
584	GO:0000082
585	GO:0006611
586	GO:0035137
587	GO:0007127
588	GO:0010714
589	GO:0032967
590	GO:0030117
591	GO:0048475
592	GO:0032559
593	GO:0009408
594	GO:0007519
595	GO:0048639
596	GO:0060538
597	secondary_structure_distribution_H.0.0_mean
598	GO:0071695
599	GO:0030162
600	GO:0031023
601	GO:0005667
602	GO:0043566
603	GO:0030018
604	GO:0060429
605	GO:0048339

606	GO:0000307
607	GO:0045786
608	GO:0045668
609	GO:0006809
610	GO:0046209
611	GO:0043537
612	GO:0016538
613	GO:0009790
614	GO:0002286
615	GO:0048820
616	GO:0046633
617	GO:0043085
618	GO:0032868
619	GO:0010648
620	GO:0048646
621	GO:0044246
622	GO:0001952
623	GO:0008637
624	GO:0007173
625	GO:0051402
626	GO:0070997
627	GO:0043551
628	GO:0090218
629	GO:0043552
630	GO:0031667
631	GO:0002685
632	GO:0060255
633	GO:0001655
634	GO:0070507
635	GO:0010811
636	GO:0002761
637	AA_composition_A_mean
638	GO:0046902
639	GO:0030889
640	GO:0043025
641	GO:0044297
642	GO:0030032
643	GO:0002237
644	solvent_accessibility_distribution_H.0.25_max
645	GO:0040012
646	GO:0051094
647	GO:0007566
648	GO:0050804
649	GO:0003705
650	GO:0035326
651	GO:0001569
652	GO:0000003

653	GO:0048469
654	VanDerWaal_distribution_P.0.5_mean
655	GO:0009267
656	GO:0016564
657	in_local_density_0.9_max
658	GO:0045066
659	GO:0051240
660	GO:0050996
661	polarizability_distribution_N.0.0_max
662	GO:0045637
663	GO:0043193
664	GO:0051726
665	GO:0051318
666	GO:0000080
667	polarizability_composition_P_max
668	GO:0060541
669	out_in_topological_max
670	GO:0006605
671	GO:0050808
672	GO:0045649
673	GO:0050792
674	GO:0003702
675	GO:0010389
676	AA_composition_G_max
677	GO:0000226
678	GO:0005200
679	GO:0019904
680	GO:0046620
681	GO:0030554
682	GO:0033267
683	GO:0007167
684	GO:0051172
685	GO:0010558
686	GO:0045934
687	GO:0045444
688	GO:0033043
689	VanDerWaal_distribution_N.0.75_max
690	AA_composition_F_mean
691	GO:0007169
692	GO:0043235
693	GO:0048041
694	GO:0032409
695	hydrophobicity_transition_PH_max
696	GO:0008152
697	GO:0030218
698	GO:0048511
699	GO:0031399

700	GO:0048745
701	AA_composition_I_max
702	GO:0043278
703	GO:0014072
704	GO:0005856
705	GO:0035264
706	GO:0008643
707	GO:0019898
708	GO:0001944
709	GO:0001568
710	GO:0050795
711	GO:0048468
712	GO:0007243
713	GO:0030031
714	GO:0045579
715	GO:0022415
716	GO:0016032
717	GO:0032869
718	GO:0051239
719	GO:0048144
720	GO:0048145
721	GO:0030098
722	GO:0030858
723	GO:0051246
724	GO:0045834
725	GO:0030296
726	GO:0035265
727	GO:0050806
728	GO:0045749
729	GO:0044093
730	GO:0030035
731	GO:0045685
732	GO:0030335
733	GO:0031988
734	polarity_distribution_H.0.75_max
735	GO:0007507
736	GO:0032964
737	GO:0007162
738	GO:0031099
739	GO:0048340
740	polarity_distribution_N.0.25_max
741	GO:0046827
742	GO:0001968
743	secondary_structure_distribution_H.1.0_mean
744	polarizability_composition_N_mean
745	GO:0045639
746	GO:0019218

747	GO:0051338
748	GO:0043549
749	GO:0045859
750	GO:0050764
751	VanDerWaal_transition_PN_max
752	GO:0050848
753	GO:0050850
754	GO:0046640
755	AA_composition_K_max
756	GO:0048520
757	AA_composition_W_mean
758	GO:0043242
759	GO:0032092
760	GO:0001701
761	GO:0009792
762	GO:0007423
763	GO:0043009
764	GO:0033365
765	GO:0034504
766	GO:0031349
767	GO:0006917
768	GO:0012502
769	GO:0043535
770	GO:0006906
771	GO:0060350
772	GO:0005154
773	GO:0044092
774	GO:0050920
775	GO:0045862
776	GO:0030155
777	GO:0050921
778	GO:0030324
779	GO:0030323
780	GO:0042476
781	GO:0045833
782	GO:0051495
783	AA_composition_F_max
784	GO:0051100
785	GO:0016566
786	GO:0051250
787	GO:0002695
788	hydrophobicity_distribution_N.0.5_max
789	GO:0048286
790	GO:0002244
791	GO:0030163
792	GO:0002347
793	GO:0042090

794	GO:0060556
795	GO:0042368
796	GO:0060558
797	GO:0051099
798	GO:0030656
799	GO:0045084
800	GO:0045075
801	GO:0015758
802	GO:0046323
803	GO:0015749
804	GO:0008645
805	GO:0030315
806	GO:0070423
807	GO:0070431
808	GO:0009894
809	GO:0023052
810	GO:0009581
811	GO:0008624
812	GO:0045939
813	GO:0010894
814	GO:0043244
815	GO:0022607
816	GO:0010921
817	GO:0032800
818	GO:0033002
819	GO:0005575
820	GO:0032088
821	VanDerWaal_transition_PH_mean
822	GO:0050870
823	GO:0010605
824	GO:0009892
825	GO:0031324
826	GO:0001782
827	GO:0008150
828	GO:0051325
829	GO:0051329
830	GO:0030100
831	GO:0043087
832	GO:0051968
833	GO:0032844
834	VanDerWaal_distribution_N.0.0_max
835	GO:0005524
836	GO:0001883
837	GO:0001882
838	GO:0010827
839	GO:0002690
840	GO:0002688

841	AA_composition_R_max
842	GO:0050820
843	GO:0008134
844	GO:0048872
845	hydrophobicity_distribution_P.0.0_max
846	polarity_distribution_H.0.25_max
847	GO:0001772
848	GO:0071375
849	AA_composition_D_mean
850	GO:0007399
851	GO:0043542
852	GO:0032386
853	GO:0042391
854	GO:0042169
855	GO:0046822
856	GO:0033157
857	GO:0043548
858	GO:0043433
859	GO:0090048
860	GO:0022407
861	GO:0007528
862	GO:0001656
863	GO:0046824
864	GO:0008104
865	GO:0000077
866	GO:0004713
867	GO:0043005
868	GO:0009582
869	hydrophobicity_composition_H_max
870	GO:0017016
871	GO:0051222
872	GO:0051052
873	GO:0060284
874	GO:0030334
875	GO:0005070
876	GO:0055114
877	GO:0032612
878	GO:0032652
879	GO:0032611
880	GO:0032651
881	GO:0022409
882	GO:0032583
883	GO:0005057
884	GO:0043434
885	GO:0000187
886	GO:0019058
887	GO:0033081

888	GO:0002699
889	GO:0044085
890	GO:0009950
891	GO:0043392
892	GO:0009925
893	GO:0045178
894	GO:0006417
895	GO:0001816
896	GO:0031346
897	GO:0060491
898	GO:0034329
899	GO:0001570
900	GO:0046321
901	GO:0032504
902	GO:0048609
903	GO:0010559
904	GO:0031333
905	GO:0010171
906	GO:0010942
907	GO:0043065
908	GO:0043068
909	GO:0002260
910	GO:0042592
911	GO:0051641
912	GO:0051017
913	GO:0032886
914	GO:0019887
915	GO:0048585
916	GO:0035295
917	GO:0035239
918	AA_composition_E_mean
919	GO:0010959
920	GO:0009896
921	GO:0001932
922	GO:0031347
923	polarizability_transition_PH_max
924	GO:0002262
925	GO:0043241
926	GO:0032984
927	GO:0031644
928	GO:0034330
929	GO:0021953
930	GO:0003697
931	GO:0030297
932	GO:0010564
933	GO:0010745
934	GO:0017145

935	GO:0002520
936	GO:0048534
937	GO:0010712
938	GO:0032965
939	GO:0045730
940	GO:0010922
941	GO:0007565
942	GO:0043491
943	GO:0050793
944	GO:0001776
945	GO:0070482
946	GO:0042130
947	GO:0048483
948	GO:0051015
949	GO:0051020
950	GO:0045768
951	GO:0006970
952	out_locta_density_0.2_mean
953	GO:0017076
954	GO:0032663
955	GO:0004693
956	GO:0022403
957	hydrophobicity_distribution_N.0.0_max
958	GO:0010039
959	GO:0014902
960	GO:0032268
961	GO:0045651
962	GO:0051223
963	GO:0046631
964	GO:0016563
965	GO:0021940
966	GO:0021936
967	GO:0000302
968	secondary_structure_distribution_P.0.0_max
969	GO:0002248
970	GO:0031331
971	GO:0051494
972	GO:0043269
973	GO:0051170
974	GO:0006606
975	GO:0005916
976	GO:0033554
977	GO:0014704
978	GO:0044291
979	GO:0042108
980	GO:0033619
981	GO:0044257

982	GO:0051603
983	GO:0046325
984	GO:0065008
985	GO:0002009
986	GO:0007416
987	GO:0048514
988	GO:0007275
989	GO:0008584
990	GO:0021954
991	GO:0005952
992	GO:0065009
993	GO:0010721
994	GO:0006928
995	GO:0050768
996	GO:0048870
997	GO:0016477
998	GO:0051674
999	GO:0050863
1000	GO:0008585
1001	GO:0032388
1002	GO:0002507
1003	GO:0042326
1004	GO:0004860
1005	GO:0045445
1006	GO:0019210
1007	GO:0022414
1008	GO:0045940
1009	GO:0006996
1010	secondary_structure_distribution_H.0.25_mean
1011	GO:0032553
1012	GO:0032555
1013	GO:0044424
1014	GO:0019059
1015	GO:0052547
1016	GO:0043281
1017	GO:0052548
1018	GO:0022603
1019	GO:0016342
1020	GO:0002274
1021	GO:0016491
1022	GO:0044265
1023	out_locta_density_0_mean
1024	out_locta_density_0.1_mean
1025	polarity_distribution_P.0.25_mean
1026	GO:0043353
1027	GO:0000910
1028	GO:0043500

1029	GO:0010875
1030	GO:0010874
1031	GO:0010035
1032	GO:0031226
1033	GO:0048547
1034	GO:0048583
1035	GO:0004683
1036	VanDerWaal_distribution_N.0.25_max
1037	GO:0031267
1038	GO:0051896
1039	GO:0001948
1040	GO:0006298
1041	solvent_accessibility_distribution_H.1.0_mean
1042	GO:0031668
1043	GO:0071496
1044	solvent_accessibility_distribution_H.0.0_mean
1045	GO:0033032
1046	GO:0051259
1047	polarity_composition_P_max
1048	GO:0060627
1049	GO:0071310
1050	hydrophobicity_distribution_H.0.25_mean
1051	polarity_distribution_N.0.25_mean
1052	GO:0030674
1053	GO:0050918
1054	GO:0045671
1055	GO:0051403
1056	GO:0050790
1057	GO:0010552
1058	GO:0051098
1059	GO:0050901
1060	GO:0030194
1061	GO:0048599
1062	GO:0009994
1063	GO:0045309
1064	GO:0019209
1065	GO:0051059
1066	GO:0060606
1067	GO:0030224
1068	GO:0001843
1069	first_singular_values
1070	GO:0010224
1071	GO:0010888
1072	GO:0007266
1073	GO:0030027
1074	GO:0009651
1075	GO:0045884

1076	VanDerWaal_distribution_N.0.75_mean
1077	GO:0042641
1078	polarizability_distribution_P.0.0_max
1079	GO:0005819
1080	GO:0007346
1081	GO:0009986
1082	GO:0045742
1083	AA_composition_E_max
1084	GO:0046885
1085	GO:0051145
1086	GO:0001953
1087	GO:0046666
1088	GO:0008629
1089	GO:0002053
1090	GO:0010464
1091	GO:0033044
1092	GO:0046903
1093	GO:0032940
1094	GO:0070851
1095	GO:0046545
1096	GO:0046660
1097	GO:0007616
1098	GO:0050868
1099	GO:0022008
1100	GO:0032332
1101	GO:0002366
1102	GO:0002263
1103	GO:0006886
1104	GO:0005938
1105	GO:0030175
1106	GO:0032330
1107	GO:0061035
1108	GO:0060326
1109	GO:0060349
1110	AA_composition_A_max
1111	GO:0007431
1112	GO:0010862
1113	GO:0017022
1114	VanDerWaal_distribution_P.0.25_max
1115	GO:0016909
1116	GO:0048871
1117	GO:0030048
1118	GO:0006275
1119	GO:0045785
1120	GO:0030295
1121	GO:0031100
1122	polarizability_transition_NH_mean

1123	GO:0002285
1124	GO:0030666
1125	GO:0010594
1126	GO:0060341
1127	GO:0048565
1128	GO:0001934
1129	GO:0004861
1130	GO:0000278
1131	GO:0022402
1132	GO:0031648
1133	GO:0031571
1134	GO:0032569
1135	GO:0010551
1136	GO:0030877
1137	GO:0002246
1138	GO:0035257
1139	hydrophobicity_transition_PN_mean
1140	GO:0051592
1141	GO:0051893
1142	GO:0090109
1143	hydrophobicity_distribution_P.0.75_max
1144	GO:0045294
1145	GO:0007610
1146	GO:0030742
1147	GO:0016023
1148	GO:0019722
1149	GO:0045807
1150	polarizability_transition_PN_max
1151	GO:0043900
1152	GO:0001837
1153	GO:0000060
1154	GO:0051090
1155	GO:0051101
1156	GO:0043388
1157	GO:0090046
1158	GO:0007213
1159	GO:0017038
1160	GO:0042493
1161	GO:0045995
1162	GO:0010829
1163	GO:0046847
1164	GO:0050798
1165	GO:0032623
1166	GO:0014037
1167	GO:0008385
1168	GO:0042094
1169	GO:0032615

1170	GO:0032655
1171	GO:0003779
1172	GO:0000790
1173	GO:0051493
1174	GO:0005622
1175	GO:0045598
1176	GO:0014074
1177	GO:0007155
1178	GO:0022610
1179	GO:0005912
1180	GO:0070161
1181	GO:0051960
1182	GO:0031528
1183	polarizability_composition_H_mean
1184	VanDerWaal_composition_H_mean
1185	GO:0044106
1186	GO:0016614
1187	GO:0016903
1188	GO:0005769
1189	GO:0002521
1190	polarity_distribution_N.0.0_mean
1191	GO:0002708
1192	GO:0002705
1193	GO:0030183
1194	GO:0034097
1195	GO:0080090
1196	GO:0002573
1197	GO:0043234
1198	GO:0001817
1199	GO:0048524
1200	polarity_composition_H_mean
1201	GO:0031669
1202	GO:0010038
1203	GO:0034101
1204	GO:0014020
1205	GO:0031143
1206	GO:0005955
1207	GO:0050732
1208	GO:0001666
1209	GO:0033273
1210	GO:0030308
1211	GO:0007613
1212	GO:0019002
1213	GO:0046983
1214	GO:0032989
1215	GO:0018209
1216	GO:0030879

1217	GO:0042475
1218	GO:0043502
1219	GO:0005884
1220	GO:0007267
1221	GO:0002437
1222	GO:0006446
1223	GO:0019003
1224	GO:0000018
1225	GO:0030099
1226	GO:0004712
1227	GO:0004708
1228	GO:0043170
1229	GO:0048584
1230	GO:0045732
1231	GO:0010893
1232	GO:0016322
1233	GO:0032695
1234	GO:0005902
1235	hydrophobicity_transition_NH_max
1236	GO:0010543
1237	GO:0045885
1238	GO:0010720
1239	GO:0002021
1240	GO:0050794
1241	GO:0002683
1242	GO:0051602
1243	GO:0044281
1244	GO:0051930
1245	GO:0051931
1246	GO:0032270
1247	GO:0005021
1248	GO:0051247
1249	solvent_accessibility_distribution_H.0.5_mean
1250	GO:0045165
1251	GO:0060205
1252	GO:0031983
1253	GO:0048806
1254	GO:0031674
1255	GO:0042995
1256	GO:0016328
1257	GO:0051701
1258	GO:0010843
1259	GO:0044212
1260	GO:0010553
1261	GO:0050821
1262	GO:0010639
1263	polarizability_composition_N_max

1264	GO:0006903
1265	polarity_distribution_H.0.0_max
1266	GO:0000075
1267	GO:0007089
1268	GO:0031570
1269	GO:0031032
1270	GO:0030239
1271	GO:0045214
1272	GO:0015629
1273	GO:0010468
1274	GO:0000165
1275	GO:0009968
1276	GO:0023057
1277	GO:0050803
1278	GO:0010810
1279	GO:0045861
1280	GO:0050789
1281	GO:0004714
1282	GO:0000902
1283	GO:0008092
1284	GO:0030705
1285	GO:0007051
1286	GO:0043232
1287	GO:0044430
1288	GO:0043228
1289	GO:0019897
1290	GO:0010463
1291	GO:0030425
1292	AA_composition_L_max
1293	GO:0030695
1294	GO:0080135
1295	GO:0043062
1296	GO:0002682
1297	GO:0050776
1298	GO:0032372
1299	GO:0032375
1300	GO:0002532
1301	GO:0071636
1302	GO:0021766
1303	GO:0044444
1304	GO:0040018
1305	GO:0007435
1306	GO:0060389
1307	GO:0008384
1308	polarizability_transition_NH_max
1309	GO:0005887
1310	GO:0042516

1311	GO:0034113
1312	GO:0060393
1313	GO:0035272
1314	polarity_distribution_H.0.5_max
1315	GO:0060589
1316	GO:0002709
1317	GO:0051241
1318	GO:0002711
1319	GO:0002702
1320	GO:0045191
1321	GO:0045911
1322	GO:0045830
1323	GO:0032722
1324	GO:0051336
1325	GO:0070491
1326	GO:0031329
1327	GO:0050900
1328	GO:0051279
1329	GO:0050852
1330	GO:0050709
1331	GO:0042306
1332	GO:0090047
1333	GO:0008593
1334	GO:0051091
1335	GO:0044456
1336	GO:0007420
1337	GO:0043112
1338	GO:0043394
1339	GO:0030863
1340	GO:0031334
1341	GO:0004707
1342	GO:0031982
1343	GO:0014013
1344	GO:0042102
1345	GO:0007269
1346	GO:0048645
1347	GO:0001759
1348	GO:0001654
1349	GO:0032845
1350	AA_composition_C_mean
1351	GO:0046677
1352	GO:0051345
1353	GO:0021781
1354	GO:0012505
1355	GO:0002429
1356	GO:0031016
1357	GO:0045648

1358	GO:0008284
1359	GO:0006836
1360	GO:0048284
1361	GO:0046626
1362	GO:0016620
1363	GO:0006081
1364	GO:0034641
1365	GO:0016616
1366	GO:0004028
1367	GO:0046641
1368	GO:0048536
1369	GO:0030054
1370	GO:0043297
1371	GO:0017166
1372	GO:0048699
1373	GO:0043616
1374	GO:0035313
1375	GO:0018107
1376	GO:0042787
1377	GO:0009897
1378	GO:0007417
1379	GO:0045682
1380	GO:0045684
1381	GO:0001525
1382	GO:0002645
1383	GO:0002643
1384	GO:0065007
1385	GO:0030029
1386	GO:0030016
1387	GO:0032432
1388	GO:0001725
1389	GO:0030676
1390	GO:0035023
1391	GO:0044449
1392	GO:0044433
1393	GO:0001657
1394	GO:0042098
1395	GO:0009952
1396	GO:0048567
1397	GO:0007439
1398	GO:0060986
1399	GO:0032352
1400	GO:0046886
1401	GO:0042035
1402	GO:0002011
1403	GO:0045792
1404	GO:0031625

1405	GO:0048246
1406	GO:0030291
1407	GO:0048489
1408	GO:0019199
1409	GO:0046634
1410	GO:0016247
1411	GO:0060323
1412	GO:0060325
1413	GO:0060322
1414	GO:0042522
1415	GO:0060324
1416	GO:0032963
1417	GO:0030036
1418	GO:0019838
1419	GO:0010523
1420	GO:0009987
1421	GO:0060795
1422	GO:0032331
1423	VanDerWaal_distribution_H.0.25_max
1424	polarizability_distribution_H.0.25_max
1425	GO:0007172
1426	GO:0045076
1427	GO:0045086
1428	GO:0046888
1429	GO:0002821
1430	GO:0002824
1431	GO:0005161
1432	GO:0016772
1433	GO:0045184
1434	GO:0031701
1435	GO:0046332
1436	GO:0014910
1437	GO:0040036
1438	GO:0030182
1439	GO:0019048
1440	GO:0030139
1441	GO:0003015
1442	GO:0060047
1443	GO:0032411
1444	GO:0060395
1445	GO:0016651
1446	GO:0032991
1447	GO:0015800
1448	GO:0006413
1449	GO:0015711
1450	GO:0015813
1451	GO:0048070

1452	GO:0032945
1453	GO:0048729
1454	GO:0046006
1455	GO:0070664
1456	GO:0050672
1457	VanDerWaal_transition_PH_max
1458	secondary_structure_transition_NH_max
1459	GO:0008603
1460	GO:0043624
1461	GO:0034623
1462	GO:0050851
1463	GO:0043534
1464	GO:0045926
1465	GO:0048638
1466	GO:0030900
1467	in_in_topological_max
1468	GO:0002218
1469	GO:0032387
1470	GO:0046823
1471	GO:0002758
1472	GO:0005089
1473	VanDerWaal_transition_NH_mean
1474	polarizability_distribution_N.0.75_max
1475	GO:0031102
1476	GO:0001773
1477	GO:0004716
1478	GO:0051489
1479	GO:0051491
1480	secondary_structure_composition_H_max
1481	in_locta_density_0.3_mean
1482	GO:0030593
1483	GO:0032410
1484	GO:0050881
1485	GO:0050879
1486	GO:0000086
1487	GO:0071158
1488	GO:0006977
1489	GO:0002684
1490	topological_change_0.6_0.7
1491	GO:0051649
1492	GO:0022604
1493	GO:0070661
1494	GO:0050865
1495	GO:0050867
1496	GO:0002696
1497	GO:0002694
1498	GO:0046651

1499	GO:0042129
1500	GO:0032943
1501	GO:0051249
1502	GO:0051251
1503	graph_density
1504	GO:0030316
1505	GO:0005623
1506	GO:0044464
1507	GO:0008156
1508	GO:0042492
1509	GO:0046629
1510	GO:0048011
1511	GO:0019226
1512	GO:0007268
1513	GO:0031558
1514	GO:0031557
1515	solvent_accessibility_distribution_H.0.25_mean
1516	GO:0051224
1517	GO:0003674
1518	GO:0071495
1519	GO:0032870
1520	GO:0032589
1521	GO:0007044
1522	GO:0016323
1523	GO:0045063
1524	GO:0002819
1525	GO:0070663
1526	GO:0032944
1527	GO:0050670
1528	GO:0002822
1529	GO:0044267
1530	GO:0046668
1531	GO:0031252
1532	secondary_structure_transition_PH_max
1533	GO:0050727
1534	polarizability_distribution_H.0.0_max
1535	VanDerWaal_distribution_H.0.0_max
1536	polarity_distribution_P.0.0_max
1537	hydrophobicity_distribution_H.0.0_max
1538	out_local_density_0.9_max
1539	GO:0046636
1540	GO:0045620
1541	GO:0071706
1542	polarizability_distribution_N.0.25_max
1543	GO:0032874
1544	GO:0032872
1545	GO:0043292

1546	GO:0006898
1547	AA_composition_Y_mean
1548	AA_composition_M_mean
1549	GO:0043412
1550	GO:0019538
1551	GO:0042107
1552	GO:0042089
1553	GO:0032371
1554	GO:0032374
1555	GO:0051925
1556	GO:0017134
1557	GO:0045839
1558	GO:0051784
1559	GO:0045577
1560	GO:0046632
1561	GO:0021955
1562	GO:0043525
1563	GO:0007405
1564	GO:0010812
1565	GO:0021872
1566	GO:0051046
1567	GO:0060090
1568	GO:0042226
1569	GO:0035249
1570	GO:0031623
1571	GO:0033036
1572	in_locta_density_0.7_mean
1573	GO:0031293
1574	GO:0051092
1575	GO:0002294
1576	GO:0043367
1577	GO:0002293
1578	GO:0002287
1579	GO:0002292
1580	GO:0043370
1581	GO:0045630
1582	GO:0043372
1583	GO:0002828
1584	GO:0042092
1585	GO:0042093
1586	GO:0050708
1587	GO:0045628
1588	GO:0045064
1589	GO:0019894
1590	GO:0051261
1591	VanDerWaal_composition_P_mean
1592	GO:0030947

1593	GO:0048010
1594	GO:0043536
1595	GO:0010595
1596	GO:0030424
1597	GO:0004030
1598	GO:0005759
1599	GO:0051540
1600	GO:0051536
1601	GO:0006520
1602	GO:0006519
1603	GO:0031980
1604	GO:0046700
1605	GO:0019717
1606	GO:0051220
1607	GO:0043086
1608	VanDerWaal_distribution_N.0.5_max
1609	GO:0002664
1610	GO:0002517
1611	GO:0002666
1612	GO:0001501
1613	GO:0002440
1614	GO:0032101
1615	GO:0051208
1616	GO:0071634
1617	GO:0071604
1618	GO:0034405
1619	GO:0048706
1620	GO:0010563
1621	GO:0045936
1622	GO:0050730
1623	GO:0060562
1624	GO:0060553
1625	GO:0009889
1626	GO:0031326
1627	GO:0070266
1628	GO:0019222
1629	GO:0060555
1630	GO:0060545
1631	GO:0060544
1632	GO:0043280
1633	GO:0010952
1634	GO:0051171
1635	GO:0044403
1636	GO:0045672
1637	GO:0004869
1638	GO:0021675
1639	GO:0051216

1640	GO:0045168
1641	GO:0031128
1642	GO:0007010
1643	GO:0007171
1644	GO:0007252
1645	GO:0033256
1646	GO:0070411
1647	GO:0048341
1648	GO:0000578
1649	GO:0003700
1650	GO:0050767
1651	GO:0048598
1652	GO:0001947
1653	GO:0048709
1654	GO:0010939
1655	GO:0010467
1656	GO:0007176
1657	GO:0048678
1658	GO:0051427
1659	GO:0010469
1660	GO:0010556
1661	GO:0045634
1662	GO:0050932
1663	GO:0050966
1664	GO:0001960
1665	GO:0050765
1666	GO:0016525
1667	GO:0033327
1668	GO:0035258
1669	GO:0018193
1670	GO:0001784
1671	GO:0045202
1672	GO:0045321
1673	polarity_composition_N_mean
1674	second_singular_values
1675	GO:0023056
1676	GO:0009967
1677	GO:0032604
1678	GO:0042253
1679	GO:0030539
1680	GO:0006919
1681	GO:0005158
1682	polarizability_distribution_P.0.75_mean
1683	out_locta_density_0.7_mean
1684	GO:0000188
1685	GO:0005520
1686	GO:0031994

1687	in_locta_density_0.2_mean
1688	GO:0008635
1689	GO:0042770
1690	GO:0010574
1691	GO:0010573
1692	GO:0010575
1693	GO:0051895
1694	GO:0006464
1695	GO:0042542
1696	GO:0018108
1697	GO:0002228
1698	GO:0042267
1699	GO:0018212
1700	GO:0048636
1701	GO:0045844
1702	GO:0048643
1703	GO:0045740
1704	GO:0042634
1705	GO:0051797
1706	GO:0051798
1707	GO:0000166
1708	GO:0001836
1709	GO:0051301
1710	GO:0001704
1711	GO:0000123
1712	GO:0002720
1713	GO:0042060
1714	GO:0005088
1715	GO:0006909
1716	GO:0046635
1717	GO:0009306
1718	GO:0048878
1719	GO:0008360
1720	GO:0006939
1721	GO:0017157
1722	GO:0030165
1723	GO:0050974
1724	GO:0050982
1725	GO:0005083
1726	GO:0032103
1727	GO:0031663
1728	GO:0051607
1729	in_out_topological_max
1730	GO:0060390
1731	GO:0007184
1732	GO:0032469
1733	GO:0005160

1734	GO:0034713
1735	GO:0060391
1736	GO:0033160
1737	GO:0001547
1738	GO:0051781
1739	GO:0033158
1740	GO:0010717
1741	GO:0010770
1742	GO:0060021
1743	GO:0010718
1744	GO:0007179
1745	GO:0030055
1746	GO:0005925
1747	GO:0005924
1748	GO:0001658
1749	GO:0060675
1750	GO:0048663
1751	GO:0030551
1752	GO:0051347
1753	GO:0033674
1754	GO:0001726
1755	GO:0007043
1756	GO:0005913
1757	GO:0015031
1758	polarizability_transition_PN_mean
1759	GO:0002544
1760	GO:0060351
1761	GO:0035176
1762	GO:0021895
1763	GO:0051546
1764	GO:0002703
1765	GO:0001911
1766	GO:0002707
1767	GO:0031342
1768	GO:0002704
1769	GO:0070603
1770	GO:0016514
1771	GO:0032376
1772	GO:0032373
1773	AA_composition_Y_max
1774	GO:0006351
1775	GO:0006355
1776	weight_edge_variance.without_missing_edge.
1777	weight_edge_variance.with_missing_edge.
1778	GO:0002712
1779	GO:0002889
1780	GO:0046330

1781	GO:0060396
1782	GO:0050671
1783	GO:0032946
1784	GO:0071378
1785	GO:0060416
1786	GO:0070665
1787	GO:0008633
1788	GO:0030888
1789	GO:0009948
1790	GO:0042562
1791	GO:0005085
1792	GO:0042501
1793	GO:0008083
1794	GO:0001823
1795	GO:0031344
1796	GO:0006955
1797	GO:0030030
1798	GO:0032507
1799	GO:0045185
1800	GO:0003009
1801	GO:0007369
1802	GO:0002275
1803	GO:0016604
1804	polarizability_distribution_P.0.25_mean
1805	GO:0016831
1806	GO:0014812
1807	GO:0014909
1808	GO:0002757
1809	GO:0002764
1810	GO:0001937
1811	GO:0002253
1812	GO:0051209
1813	GO:0051283
1814	GO:0051282
1815	GO:0001958
1816	GO:0006968
1817	GO:0031406
1818	GO:0051898
1819	GO:0070688
1820	GO:0030010
1821	GO:0035091
1822	GO:0045860
1823	GO:0010927
1824	GO:0051054
1825	GO:0005159
1826	GO:0031103
1827	GO:0043011

1828	hydrophobicity_composition_P_mean
1829	GO:0030111
1830	GO:0004033
1831	GO:0008194
1832	GO:0009308
1833	GO:0009310
1834	GO:0051188
1835	GO:0042573
1836	GO:0051186
1837	GO:0030330
1838	GO:0022411
1839	GO:0010741
1840	GO:0048548
1841	GO:0006907
1842	GO:0051704
1843	GO:0030278
1844	GO:0045778
1845	GO:0030949
1846	GO:0042327
1847	GO:0045937
1848	GO:0010562
1849	GO:0050431
1850	GO:0009266
1851	GO:0045821
1852	polarity_distribution_H.1.0_mean
1853	GO:0044463
1854	GO:0042523
1855	GO:0002456
1856	GO:0030427
1857	GO:0010560
1858	polarity_distribution_N.0.5_mean
1859	GO:0060249
1860	solvent_accessibility_distribution_H.1.0_max
1861	GO:0021697
1862	GO:0043113
1863	GO:0043586
1864	GO:0050912
1865	GO:0001580
1866	GO:0046890
1867	GO:0043254
1868	GO:0001841
1869	GO:0060485
1870	GO:0001775
1871	GO:0004705
1872	GO:0048610
1873	hydrophobicity_distribution_P.1.0_mean
1874	GO:0042113

1875	GO:0006310
1876	GO:0045112
1877	GO:0031295
1878	GO:0031294
1879	GO:0009303
1880	GO:0030238
1881	GO:0010869
1882	GO:0016358
1883	AA_composition_H_mean
1884	polarity_transition_NH_mean
1885	GO:0060079
1886	GO:0002768
1887	GO:0002376
1888	GO:0007250
1889	GO:0019842
1890	GO:0051055
1891	GO:0016079
1892	GO:0000922
1893	GO:0048008
1894	GO:0045622
1895	GO:0045624
1896	GO:0035282
1897	GO:0032633
1898	GO:0032673
1899	GO:0010647
1900	GO:0017048
1901	GO:0010657
1902	GO:0010660
1903	GO:0007049
1904	VanDerWaal_distribution_N.0.25_mean
1905	GO:0043395
1906	GO:0046638
1907	GO:0046637
1908	GO:0055082
1909	GO:0046625
1910	GO:0006873
1911	GO:0000940
1912	GO:0016331
1913	GO:0019219
1914	GO:0002221
1915	GO:0042308
1916	GO:0042992
1917	GO:0042994
1918	GO:0031072
1919	GO:0009636
1920	GO:0051053
1921	GO:0010948

1922	secondary_structure_distribution_H.0.5_mean
1923	topological_change_0.2_0.3
1924	GO:0006929
1925	GO:0002700
1926	GO:0002697
1927	GO:0016573
1928	GO:0046658
1929	GO:0060081
1930	GO:0044060
1931	GO:0044448
1932	GO:0070513
1933	GO:0006975
1934	GO:0006936
1935	GO:0048596
1936	GO:0048048
1937	GO:0031076
1938	GO:0042506
1939	GO:0002040
1940	GO:0045588
1941	GO:0019725
1942	GO:0045586
1943	GO:0046645
1944	GO:0046643
1945	GO:0003824
1946	GO:0043410
1947	GO:0010799
1948	GO:0004702
1949	GO:0010800
1950	GO:0046328
1951	solvent_accessibility_transition_HE_mean
1952	GO:0005576
1953	GO:0042509
1954	GO:0042503
1955	GO:0000118
1956	GO:0016581
1957	GO:0043632
1958	GO:0019941
1959	GO:0003924
1960	GO:0042976
1961	GO:0007204
1962	GO:0045625
1963	GO:0051023
1964	GO:0002825
1965	GO:0031345
1966	GO:0001964
1967	GO:0030521
1968	GO:0048332

1969	GO:0001909
1970	GO:0001906
1971	GO:0003712
1972	secondary_structure_distribution_N.0.75_mean
1973	GO:0007090
1974	GO:0090100
1975	GO:0048407
1976	GO:0002052
1977	polarizability_distribution_N.0.5_max
1978	GO:0051656
1979	GO:0002637
1980	GO:0035108
1981	GO:0035107
1982	GO:0002706
1983	GO:0048736
1984	GO:0021533
1985	GO:0060173
1986	GO:0045177
1987	GO:0023046
1988	GO:0023060
1989	GO:0007260
1990	GO:0030838
1991	GO:0031110
1992	GO:0043497
1993	GO:0031109
1994	GO:0016830
1995	GO:0032094
1996	GO:0009057
1997	GO:0010623
1998	GO:0007584
1999	GO:0004468
2000	GO:0004402
2001	GO:0009755
2002	GO:0045686
2003	GO:0000255
2004	GO:0014014
2005	GO:0042104
2006	GO:0046449
2007	GO:0010092
2008	GO:0042552
2009	polarity_distribution_P.1.0_mean
2010	GO:0005178
2011	GO:0070822
2012	GO:0016580
2013	GO:0006911
2014	GO:0033627
2015	GO:0048863

2016	GO:0031410
2017	GO:0060078
2018	GO:0030595
2019	GO:0043436
2020	GO:0006082
2021	GO:0019752
2022	GO:0042180
2023	GO:0070167
2024	GO:0051148
2025	GO:0030500
2026	GO:0042990
2027	GO:0042991
2028	GO:0005099
2029	GO:0010940
2030	GO:0045449
2031	GO:0002369
2032	GO:0032743
2033	GO:0002726
2034	GO:0002724
2035	GO:0048260
2036	out_locta_density_0.4_mean
2037	GO:0035088
2038	GO:0002209
2039	GO:0001662
2040	GO:0043331
2041	GO:0051149
2042	AA_composition_H_max
2043	GO:0016810
2044	hydrophobicity_distribution_H.1.0_mean
2045	GO:0007442
2046	GO:0060070
2047	GO:0009880
2048	GO:0030528
2049	GO:0051252
2050	GO:0008013
2051	GO:0046850
2052	GO:0045124
2053	GO:0070252
2054	GO:0033275
2055	GO:0030049
2056	GO:0042307
2057	GO:0014896
2058	AA_composition_I_mean
2059	GO:0032273
2060	GO:0005516
2061	secondary_structure_distribution_N.0.5_mean
2062	GO:0010544

2063	GO:0004857
2064	GO:0032956
2065	GO:0055007
2066	GO:0030141
2067	GO:0007131
2068	GO:0030426
2069	GO:0042802
2070	GO:0048844
2071	GO:0060840
2072	GO:0035303
2073	GO:0051302
2074	GO:0050864
2075	GO:0060037
2076	GO:0065003
2077	GO:0043933
2078	GO:0050998
2079	secondary_structure_distribution_P.0.75_mean
2080	GO:0035136
2081	GO:0001502
2082	GO:0004709
2083	GO:0043027
2084	GO:0032909
2085	GO:0032906
2086	GO:0002548
2087	GO:0031821
2088	GO:0007389
2089	GO:0002377
2090	GO:0001708
2091	GO:0009108
2092	GO:0009309
2093	GO:0030170
2094	GO:0009063
2095	GO:0016836
2096	GO:0016758
2097	GO:0016853
2098	GO:0051287
2099	GO:0070279
2100	GO:0016835
2101	GO:0006732
2102	GO:0016757
2103	GO:0044270
2104	GO:0034621
2105	GO:0043623
2106	GO:0034622
2107	GO:0000781
2108	GO:0002698
2109	GO:0051918

2110	GO:0051917
2111	GO:0070587
2112	GO:0010470
2113	GO:0050710
2114	GO:0002374
2115	GO:0002740
2116	GO:0070586
2117	GO:0034114
2118	GO:0002739
2119	GO:0031323
2120	GO:0050778
2121	GO:0010627
2122	GO:0042345
2123	GO:0042348
2124	GO:0033198
2125	GO:0010769
2126	GO:0008045
2127	GO:0032835
2128	GO:0072006
2129	GO:0042228
2130	GO:0048710
2131	secondary_structure_distribution_P.0.5_mean
2132	GO:0014855
2133	GO:0055017
2134	GO:0060038
2135	GO:0060419
2136	GO:0033344
2137	in_locta_density_0.4_mean
2138	GO:0043565
2139	GO:0031090
2140	GO:0016746
2141	GO:0006767
2142	GO:0003713
2143	GO:0046530
2144	GO:0019787
2145	VanDerWaal_distribution_N.1.0_mean
2146	secondary_structure_distribution_P.0.0_mean
2147	GO:0032640
2148	GO:0032680
2149	GO:0048675
2150	GO:0043967
2151	GO:0051048
2152	GO:0008307
2153	GO:0016460
2154	GO:0005859
2155	GO:0045581
2156	GO:0044058

2157	GO:0032269
2158	GO:0048814
2159	GO:0050773
2160	GO:0006937
2161	polarity_transition_PN_max
2162	GO:0044236
2163	GO:0006972
2164	GO:0072175
2165	GO:0035148
2166	GO:0001838
2167	GO:0009620
2168	GO:0015026
2169	GO:0044283
2170	in_locta_density_0_mean
2171	GO:0007530
2172	in_out_topological_mean
2173	GO:0050907
2174	GO:0050913
2175	GO:0010226
2176	GO:0005509
2177	GO:0006308
2178	GO:0008630
2179	GO:0042472
2180	GO:0070304
2181	GO:0060348
2182	GO:0003002
2183	GO:0043487
2184	GO:0043467
2185	GO:0032148
2186	GO:0016504
2187	GO:0060048
2188	polarizability_distribution_N.0.5_mean
2189	AA_composition_V_max
2190	GO:0032735
2191	GO:0048302
2192	GO:0048304
2193	GO:0048291
2194	GO:0045410
2195	GO:0045408
2196	GO:0060134
2197	GO:0004721
2198	GO:0034605
2199	in_locta_density_0.1_mean
2200	GO:0045061
2201	GO:0002088
2202	GO:0006471
2203	GO:0060158

2204	GO:0031702
2205	GO:0005730
2206	VanDerWaal_composition_H_max
2207	polarizability_composition_H_max
2208	GO:0015837
2209	GO:0002674
2210	GO:0046649
2211	GO:0050854
2212	GO:0033138
2213	GO:0007163
2214	GO:0046395
2215	GO:0016054
2216	GO:0032494
2217	GO:0070302
2218	GO:0007253
2219	GO:0042347
2220	GO:0002792
2221	GO:0005096
2222	GO:0031264
2223	GO:0006302
2224	GO:0006474
2225	GO:0018409
2226	GO:0001912
2227	GO:0018076
2228	GO:0031365
2229	GO:0018394
2230	VanDerWaal_distribution_P.0.25_mean
2231	GO:0030169
2232	GO:0042645
2233	GO:0009295
2234	GO:0032862
2235	GO:0032856
2236	GO:0042101
2237	GO:0030522
2238	GO:0043271
2239	GO:0034612
2240	GO:0050810
2241	GO:0045841
2242	GO:0071174
2243	GO:0031577
2244	GO:0071173
2245	GO:0030071
2246	GO:0007094
2247	GO:0016585
2248	GO:0001910
2249	GO:0043501
2250	GO:0021545

2251	GO:0031341
2252	GO:0014888
2253	GO:0048015
2254	GO:0005198
2255	GO:0014823
2256	GO:0070412
2257	GO:0032731
2258	GO:0032732
2259	GO:0034391
2260	GO:0034390
2261	VanDerWaal_distribution_N.0.0_mean
2262	GO:0045471
2263	GO:0043277
2264	GO:0051248
2265	GO:0048812
2266	polarizability_distribution_P.0.0_mean
2267	GO:0003001
2268	polarity_transition_PH_mean
2269	GO:0075136
2270	GO:0052173
2271	GO:0052200
2272	GO:0051176
2273	GO:0009629
2274	GO:0043197
2275	GO:0044309
2276	GO:0050663
2277	GO:0051881
2278	GO:0032957
2279	GO:0006021
2280	GO:0043647
2281	GO:0043114
2282	GO:0001818
2283	GO:0018210
2284	GO:0050819
2285	GO:0030195
2286	GO:0043496
2287	GO:0006865
2288	out_locta_density_0.3_mean
2289	GO:0032321
2290	GO:0007254
2291	GO:0046889
2292	GO:0006470
2293	GO:0044452
2294	GO:0002443
2295	GO:0050801
2296	GO:0048568
2297	GO:0007165

2298	GO:0051879
2299	hydrophobicity_distribution_H.0.0_mean
2300	VanDerWaal_distribution_H.0.0_mean
2301	polarizability_distribution_H.0.0_mean
2302	polarity_distribution_P.0.0_mean
2303	GO:0016638
2304	GO:0033764
2305	GO:0009820
2306	GO:0016229
2307	GO:0019748
2308	GO:0016628
2309	GO:0006725
2310	GO:0008210
2311	GO:0016453
2312	GO:0009055
2313	GO:0006040
2314	GO:0008347
2315	GO:0000904
2316	GO:0033205
2317	GO:0046676
2318	GO:0090278
2319	VanDerWaal_distribution_H.1.0_mean
2320	polarizability_distribution_H.1.0_mean
2321	GO:0045954
2322	GO:0031093
2323	GO:0048588
2324	GO:0002715
2325	GO:0042269
2326	GO:0002717
2327	GO:0031343
2328	GO:0001503
2329	GO:0001707
2330	GO:0031091
2331	GO:0045414
2332	GO:0045416
2333	GO:0050729
2334	GO:0046426
2335	GO:0040011
2336	GO:0042532
2337	GO:0001894
2338	GO:0032677
2339	GO:0044421
2340	GO:0005975
2341	GO:0051289
2342	GO:0050662
2343	GO:0006766
2344	GO:0048037

2345	GO:0021695
2346	GO:0021696
2347	GO:0032970
2348	GO:0051043
2349	GO:0048525
2350	GO:0051044
2351	GO:0030659
2352	GO:0007262
2353	GO:0032635
2354	GO:0032675
2355	GO:0051428
2356	GO:0030001
2357	GO:0042993
2358	GO:0032526
2359	GO:0001709
2360	GO:0048864
2361	GO:0019827
2362	GO:0031254
2363	GO:0042044
2364	GO:0051281
2365	GO:0007159
2366	GO:0005886
2367	GO:0051482
2368	GO:0001931
2369	polarizability_distribution_H.0.5_mean
2370	VanDerWaal_distribution_H.0.5_mean
2371	GO:0048365
2372	GO:0003706
2373	GO:0008625
2374	GO:0006509
2375	GO:0045773
2376	GO:0045123
2377	polarizability_distribution_P.0.5_mean
2378	GO:0005026
2379	GO:0005024
2380	GO:0034616
2381	GO:0001935
2382	GO:0001936
2383	GO:0032927
2384	GO:0007178
2385	GO:0017002
2386	GO:0016361
2387	GO:0005114
2388	GO:0005025
2389	GO:0048179
2390	GO:0004675
2391	GO:0048535

2392	GO:0060688
2393	GO:0035304
2394	GO:0000267
2395	GO:0090092
2396	GO:0042100
2397	GO:0070265
2398	GO:0043123
2399	GO:0030252
2400	GO:0051480
2401	GO:0042508
2402	GO:0031233
2403	GO:0033261
2404	GO:0030496
2405	GO:0044451
2406	GO:0002062
2407	GO:0032429
2408	GO:0032430
2409	GO:0003007
2410	VanDerWaal_distribution_P.0.0_mean
2411	GO:0051047
2412	GO:0006626
2413	GO:0070585
2414	GO:0015909
2415	GO:0051588
2416	GO:0021544
2417	GO:0021756
2418	out_clustering_max
2419	GO:0007494
2420	GO:0005771
2421	polarizability_distribution_N.0.75_mean
2422	AA_composition_R_mean
2423	GO:0043122
2424	GO:0007249
2425	GO:0002335
2426	solvent_accessibility_distribution_H.0.75_mean
2427	in_local_density_0.8_max
2428	GO:0032091
2429	GO:0010740
2430	GO:0009607
2431	GO:0005834
2432	GO:0032801
2433	GO:0045664
2434	GO:0019400
2435	GO:0050772
2436	GO:0071071
2437	GO:0071072
2438	GO:0030017

2439	hydrophobicity_composition_N_mean
2440	GO:0051806
2441	GO:0046718
2442	GO:0052192
2443	GO:0051828
2444	GO:0033005
2445	GO:0044409
2446	GO:0030260
2447	GO:0052126
2448	GO:0009798
2449	GO:0032755
2450	GO:0048771
2451	GO:0031401
2452	GO:0003085
2453	GO:0051260
2454	GO:0006511
2455	GO:0005518
2456	GO:0048738
2457	GO:0005066
2458	GO:0051238
2459	GO:0042346
2460	GO:0006461
2461	GO:0070271
2462	GO:0031584
2463	GO:0060559
2464	GO:0022614
2465	GO:0032715
2466	hydrophobicity_distribution_H.0.75_mean
2467	GO:0002026
2468	GO:0033613
2469	GO:0014031
2470	GO:0048762
2471	GO:0045661
2472	GO:0017124
2473	GO:0006875
2474	GO:0006874
2475	GO:0055074
2476	GO:0051707
2477	GO:0050896
2478	GO:0030574
2479	GO:0009615
2480	polarizability_composition_P_mean
2481	secondary_structure_distribution_N.1.0_mean
2482	hydrophobicity_distribution_N.0.0_mean
2483	GO:0042490
2484	GO:0030326
2485	GO:0035113

2486	VanDerWaal_transition_PN_mean
2487	GO:0030852
2488	GO:0030851
2489	GO:0016407
2490	GO:0021987
2491	hydrophobicity_distribution_N.1.0_mean
2492	polarity_distribution_P.0.75_mean
2493	GO:0045446
2494	GO:0003158
2495	GO:0032799
2496	hydrophobicity_distribution_N.0.5_mean
2497	GO:0034199
2498	GO:0043154
2499	GO:0010466
2500	GO:0042362
2501	GO:0015918
2502	GO:0030301
2503	GO:0045058
2504	GO:0002367
2505	GO:0002718
2506	GO:0002076
2507	GO:0061025
2508	GO:0006944
2509	GO:0032147
2510	GO:0016197
2511	GO:0035115
2512	GO:0000149
2513	GO:0042058
2514	GO:0043034
2515	GO:0048666
2516	GO:0031175
2517	GO:0030665
2518	GO:0006629
2519	GO:0006879
2520	GO:0008201
2521	GO:0000792
2522	GO:0048232
2523	GO:0007283
2524	GO:0050856
2525	GO:0005624
2526	GO:0050715
2527	AA_composition_L_mean
2528	GO:0010883
2529	GO:0051293
2530	GO:0051653
2531	GO:0044260
2532	out_local_density_0.2_max

2533	GO:0051899
2534	out_locta_density_0.9_mean
2535	GO:0042219
2536	GO:0031901
2537	topological_change_0.5_0.6
2538	GO:0030902
2539	GO:0048704
2540	GO:0042135
2541	GO:0051537
2542	GO:0009066
2543	GO:0004029
2544	GO:0042175
2545	GO:0005789
2546	GO:0003985
2547	GO:0008483
2548	GO:0006790
2549	GO:0005783
2550	GO:0042375
2551	GO:0008033
2552	GO:0006536
2553	GO:0031098
2554	GO:0001754
2555	GO:0019915
2556	GO:0006952
2557	GO:0010743
2558	AA_composition_G_mean
2559	GO:0001764
2560	GO:0003012
2561	GO:0033135
2562	GO:0001755
2563	GO:0042110
2564	GO:0032271
2565	GO:0014047
2566	GO:0014048
2567	GO:0001913
2568	GO:0001914
2569	GO:0017046
2570	GO:0001974
2571	GO:0007214
2572	GO:0033209
2573	GO:0005981
2574	GO:0045296
2575	GO:0010953
2576	GO:0010955
2577	GO:0033137
2578	GO:0070613
2579	GO:0051817

2580	GO:0044003
2581	GO:0046907
2582	hydrophobicity_distribution_P.0.25_mean
2583	GO:0048701
2584	out_clustering_mean
2585	GO:0007191
2586	GO:0007212
2587	GO:0023050
2588	GO:0007257
2589	GO:0032102
2590	GO:0003714
2591	GO:0006935
2592	GO:0030501
2593	GO:0042330
2594	GO:0030199
2595	GO:0070169
2596	GO:0030866
2597	GO:0030865
2598	GO:0005123
2599	GO:0016505
2600	GO:0008656
2601	GO:0031000
2602	GO:0043028
2603	GO:0002320
2604	GO:0007352
2605	GO:0022406
2606	GO:0005246
2607	GO:0045190
2608	GO:0016447
2609	GO:0002204
2610	GO:0002562
2611	GO:0021575
2612	GO:0002381
2613	GO:0030177
2614	GO:0048305
2615	GO:0016445
2616	GO:0016444
2617	GO:0002200
2618	GO:0002208
2619	polarizability_distribution_N.0.25_mean
2620	GO:0046427
2621	GO:0030509
2622	GO:0042977
2623	GO:0046884
2624	GO:0042713
2625	GO:0007320
2626	GO:0042531

2627	GO:0032274
2628	GO:0042517
2629	GO:0007259
2630	GO:0016863
2631	GO:0035383
2632	GO:0044282
2633	GO:0006637
2634	GO:0046838
2635	GO:0046855
2636	GO:0046488
2637	GO:0032774
2638	GO:0021532
2639	GO:0030901
2640	GO:0050871
2641	GO:0048505
2642	GO:0040034
2643	GO:0032823
2644	GO:0032825
2645	GO:0001779
2646	GO:0032816
2647	GO:0032814
2648	GO:0031638
2649	GO:0051924
2650	GO:0005007
2651	GO:0031050
2652	GO:0017015
2653	GO:0070410
2654	GO:0031111
2655	GO:0031053
2656	GO:0005072
2657	GO:0031114
2658	GO:0031047
2659	GO:0035195
2660	GO:0032847
2661	GO:0007019
2662	GO:0016441
2663	GO:0007183
2664	GO:0071359
2665	GO:0030618
2666	GO:0005071
2667	GO:0035194
2668	GO:0035196
2669	GO:0007026
2670	GO:0070918
2671	GO:0000768
2672	GO:0006949
2673	GO:0030667

2674	GO:0070555
2675	GO:0033574
2676	AA_composition_W_max
2677	GO:0005131
2678	GO:0006350
2679	GO:0046685
2680	GO:0050771
2681	GO:0021535
2682	GO:0019888
2683	GO:0019208
2684	GO:0008093
2685	GO:0045824
2686	GO:0051952
2687	polarizability_distribution_P.1.0_mean
2688	secondary_structure_distribution_P.0.25_mean
2689	GO:0004435
2690	GO:0004434
2691	GO:0004629
2692	GO:0046717
2693	GO:0043408
2694	GO:0042826
2695	GO:0042471
2696	GO:0051346
2697	GO:0006706
2698	GO:0016860
2699	GO:0007599
2700	GO:0050878
2701	GO:0002710
2702	GO:0001653
2703	GO:0001780
2704	GO:0030673
2705	GO:0050817
2706	GO:0045589
2707	GO:0008528
2708	GO:0007596
2709	GO:0002820
2710	GO:0001781
2711	GO:0046425
2712	GO:0002823
2713	GO:0005041
2714	GO:0002224
2715	GO:0051789
2716	GO:0006887
2717	GO:0050919
2718	GO:0004842
2719	GO:0045823
2720	polarity_distribution_H.0.25_mean

2721	GO:0016788
2722	GO:0045055
2723	GO:0043279
2724	GO:0032587
2725	GO:0060401
2726	GO:0060402
2727	GO:0071156
2728	GO:0032370
2729	GO:0031672
2730	GO:0045766
2731	GO:0005234
2732	GO:0005230
2733	GO:0005231
2734	GO:0030518
2735	GO:0030552
2736	GO:0002089
2737	GO:0070064
2738	GO:0005929
2739	GO:0043236
2740	GO:0044243
2741	GO:0050840
2742	GO:0008305
2743	GO:0070838
2744	GO:0007166
2745	GO:0048266
2746	GO:0015674
2747	GO:0046642
2748	GO:0023033
2749	GO:0032649
2750	GO:0006816
2751	GO:0016500
2752	secondary_structure_composition_N_mean
2753	GO:0005737
2754	out_out_topological_mean
2755	GO:0001957
2756	GO:0060041
2757	GO:0021915
2758	GO:0015459
2759	GO:0021543
2760	GO:0016042
2761	GO:0030971
2762	out_locta_density_0.6_mean
2763	GO:0034103
2764	GO:0007631
2765	GO:0005102
2766	GO:0042518
2767	GO:0003823

2768	GO:0034399
2769	GO:0016363
2770	GO:0007368
2771	GO:0043084
2772	GO:0051082
2773	GO:0050775
2774	GO:0051964
2775	GO:0051961
2776	GO:0040023
2777	GO:0051647
2778	GO:0031092
2779	polarity_distribution_N.0.75_mean
2780	GO:0042596
2781	GO:0008339
2782	GO:0045650
2783	GO:0050886
2784	polarity_distribution_H.0.0_mean
2785	GO:0005637
2786	GO:0070723
2787	topological_change_0.4_0.5
2788	GO:0050688
2789	GO:0051702
2790	GO:0060191
2791	GO:0007015
2792	GO:0045713
2793	GO:0010871
2794	GO:0045666
2795	GO:0042762
2796	GO:0000775
2797	GO:0048854
2798	GO:0006577
2799	GO:0006576
2800	GO:0046914
2801	GO:0016408
2802	GO:0044262
2803	GO:0009437
2804	GO:0009746
2805	GO:0034284
2806	GO:0009749
2807	GO:0046627
2808	GO:0030262
2809	GO:0005634
2810	GO:0000737
2811	GO:0006997
2812	GO:0006309
2813	GO:0005794
2814	GO:0043039

2815	GO:0016876
2816	GO:0043038
2817	GO:0034660
2818	GO:0004812
2819	GO:0006399
2820	GO:0016875
2821	GO:0006418
2822	in_locta_density_0.9_mean
2823	GO:0014033
2824	GO:0014032
2825	GO:0006110
2826	hydrophobicity_distribution_N.0.75_mean
2827	GO:0043208
2828	GO:0051861
2829	GO:0006950
2830	GO:0032855
2831	GO:0004222
2832	GO:0002252
2833	GO:0006473
2834	GO:0016769
2835	GO:0016790
2836	GO:0016846
2837	GO:0034655
2838	GO:0006720
2839	GO:0009225
2840	GO:0043603
2841	GO:0030151
2842	GO:0004497
2843	GO:0009067
2844	GO:0006538
2845	GO:0034656
2846	GO:0006041
2847	GO:0009065
2848	GO:0006044
2849	GO:0000785
2850	GO:0048859
2851	GO:0046639
2852	GO:0046532
2853	GO:0001649
2854	GO:0045669
2855	GO:0005138
2856	GO:0060033
2857	GO:0009299
2858	GO:0060425
2859	GO:0006071
2860	GO:0016597
2861	GO:0050714

2862	GO:0051179
2863	GO:0048477
2864	GO:0007276
2865	GO:0043499
2866	GO:0002279
2867	GO:0043303
2868	GO:0045576
2869	GO:0043299
2870	GO:0002448
2871	GO:0051590
2872	GO:0050728
2873	GO:0051354
2874	GO:0006979
2875	GO:0032787
2876	GO:0048713
2877	GO:0007626
2878	GO:0009953
2879	GO:0050829
2880	GO:0005179
2881	GO:0060591
2882	GO:0030101
2883	GO:0042056
2884	GO:0050680
2885	GO:0010884
2886	GO:0046928
2887	GO:0014003
2888	GO:0005132
2889	GO:0033008
2890	GO:0043383
2891	GO:0045060
2892	GO:0043306
2893	GO:0043302
2894	GO:0019958
2895	GO:0003254
2896	GO:0016458
2897	GO:0016567
2898	GO:0051258
2899	GO:0015630
2900	GO:0007272
2901	GO:0008366
2902	GO:0045453
2903	GO:0045211
2904	GO:0051966
2905	GO:0032947
2906	polarizability_distribution_N.1.0_mean
2907	GO:0001508
2908	GO:0031012

2909	GO:0001530
2910	GO:0005201
2911	GO:0005578
2912	GO:0044304
2913	GO:0043471
2914	GO:0043470
2915	GO:0010043
2916	GO:0008016
2917	GO:0010524
2918	GO:0007143
2919	GO:0001975
2920	GO:0050482
2921	GO:0002250
2922	GO:0032609
2923	GO:0002460
2924	GO:0044241
2925	GO:0051087
2926	GO:0005626
2927	GO:0015276
2928	GO:0022834
2929	GO:0032052
2930	GO:0007194
2931	GO:0031280
2932	GO:0001973
2933	GO:0007193
2934	GO:0051350
2935	GO:0045761
2936	GO:0043030
2937	GO:0031532
2938	polarity_distribution_P.0.5_mean
2939	GO:0030431
2940	GO:0090257
2941	GO:0006940
2942	GO:0004687
2943	GO:0044427
2944	GO:0015872
2945	GO:0000185
2946	GO:0014819
2947	GO:0007052
2948	GO:0006401
2949	GO:0006402
2950	GO:0042577
2951	GO:0046839
2952	GO:0042136
2953	GO:0043589
2954	GO:0070647
2955	GO:0032446

2956	GO:0070925
2957	GO:0000159
2958	GO:0018149
2959	GO:0048667
2960	GO:0032990
2961	GO:0005577
2962	GO:0048858
2963	GO:0031400
2964	GO:0004620
2965	GO:0001959
2966	GO:0030004
2967	GO:0051320
2968	weight_edge_mean.without_missing_edge.
2969	weight_edge_mean.with_missing_edge.
2970	GO:0000084
2971	GO:0046697
2972	GO:0001893
2973	GO:0050707
2974	GO:0001824
2975	secondary_structure_transition_PN_mean
2976	out_in_topological_variance
2977	GO:0016655
2978	GO:0004518
2979	GO:0006661
2980	GO:0051235
2981	out_local_density_0_max
2982	out_local_density_0.1_max
2983	GO:0045715
2984	GO:0045714
2985	GO:0023036
2986	GO:0023049
2987	GO:0019221
2988	GO:0023038
2989	GO:0070053
2990	GO:0045348
2991	GO:0050702
2992	GO:0050706
2993	GO:0055065
2994	GO:0050701
2995	GO:0030159
2996	GO:0050704
2997	GO:0045351
2998	GO:0030005
2999	GO:0030003
3000	GO:0030300
3001	GO:0001955
3002	GO:0032095

3003	GO:0032098
3004	GO:0032107
3005	GO:0032104
3006	GO:0031640
3007	GO:0045667
3008	GO:0021871
3009	GO:0001710
3010	GO:0005148
3011	GO:0048333
3012	GO:0005899
3013	GO:0016311
3014	GO:0005802
3015	GO:0030318
3016	GO:0050931
3017	GO:0043255
3018	GO:0006612
3019	GO:0016337
3020	GO:0005911
3021	GO:0000139
3022	GO:0044431
3023	GO:0043543
3024	GO:0001819
3025	GO:0021537
3026	GO:0045723
3027	hydrophobicity_distribution_H.0.5_mean
3028	GO:0006942
3029	hydrophobicity_distribution_P.0.0_mean
3030	GO:0044432
3031	GO:0071212
3032	GO:0005506
3033	GO:0033293
3034	GO:0042133
3035	GO:0006575
3036	GO:0019530
3037	GO:0031214
3038	GO:0006549
3039	GO:0006573
3040	GO:0003774
3041	GO:0030834
3042	GO:0007016
3043	GO:0016459
3044	GO:0030042
3045	hydrophobicity_transition_PH_mean
3046	GO:0006338
3047	GO:0006066
3048	GO:0016053
3049	GO:0046394

3050	GO:0008610
3051	GO:0046173
3052	out_local_density_0.8_max
3053	out_in_topological_mean
3054	in_clustering_max
3055	VanDerWaal_composition_N_mean
3056	GO:0009855
3057	GO:0009799
3058	GO:0030104
3059	GO:0030041
3060	GO:0008154
3061	GO:0005539
3062	GO:0007498
3063	GO:0045197
3064	GO:0007520
3065	GO:0003707
3066	GO:0004879
3067	GO:0055056
3068	GO:0043516
3069	GO:0046686
3070	GO:0005249
3071	GO:0034374
3072	GO:0001504
3073	out_locta_density_0.8_mean
3074	GO:0000819
3075	GO:0051983
3076	GO:0032155
3077	GO:0032272
3078	GO:0000146
3079	GO:0000279
3080	GO:0000718
3081	GO:0051693
3082	GO:0000070
3083	GO:0000087
3084	GO:0030837
3085	GO:0007067
3086	GO:0030835
3087	GO:0030898
3088	GO:0032153
3089	GO:0000280
3090	GO:0048285
3091	GO:0046696
3092	GO:0017137
3093	GO:0051409
3094	GO:0016854
3095	GO:0016289
3096	GO:0009410

3097	GO:0007040
3098	GO:0003988
3099	GO:0046113
3100	GO:0000062
3101	GO:0016641
3102	GO:0031985
3103	GO:0031984
3104	GO:0003857
3105	GO:0046496
3106	GO:0015020
3107	GO:0042398
3108	GO:0008652
3109	GO:0016866
3110	GO:0071466
3111	GO:0006805
3112	GO:0070330
3113	GO:0008198
3114	GO:0006769
3115	GO:0006730
3116	GO:0006721
3117	GO:0004303
3118	GO:0046112
3119	GO:0005795
3120	GO:0006733
3121	GO:0004300
3122	GO:0017144
3123	GO:0008017
3124	GO:0030073
3125	GO:0016324
3126	GO:0002831
3127	GO:0001556
3128	hydrophobicity_distribution_P.0.5_mean
3129	GO:0030662
3130	GO:0044255
3131	GO:0048662
3132	GO:0016050
3133	GO:0071157
3134	in_locta_density_0.6_mean
3135	GO:0030955
3136	GO:0019228
3137	GO:0033860
3138	GO:0060397
3139	GO:0021522
3140	GO:0021517
3141	GO:0019229
3142	in_local_density_0.6_max
3143	GO:0042803

3144	GO:0042383
3145	GO:0001756
3146	GO:0061053
3147	GO:0043507
3148	GO:0043506
3149	GO:0001890
3150	GO:0046883
3151	GO:0000045
3152	GO:0007059
3153	GO:0042805
3154	GO:0030219
3155	GO:0002839
3156	GO:0002836
3157	GO:0007200
3158	GO:0002833
3159	GO:0002837
3160	GO:0002834
3161	GO:0016236
3162	GO:0004908
3163	GO:0002855
3164	GO:0002858
3165	GO:0002860
3166	GO:0002423
3167	GO:0002857
3168	GO:0002420
3169	GO:0030282
3170	GO:0031581
3171	GO:0031589
3172	GO:0050748
3173	GO:0003676
3174	GO:0043130
3175	GO:0032182
3176	GO:0045059
3177	GO:0022037
3178	out_clustering_variance
3179	GO:0006974
3180	GO:0006921
3181	GO:0043588
3182	GO:0001938
3183	GO:0005172
3184	GO:0045779
3185	GO:0046851
3186	GO:0034104
3187	GO:0007034
3188	GO:0007041
3189	GO:0016667
3190	GO:0007126

3191	GO:0051327
3192	GO:0051321
3193	GO:0051547
3194	GO:0005062
3195	GO:0051549
3196	GO:0021587
3197	AA_composition_N_mean
3198	GO:0003677
3199	GO:0003016
3200	GO:0019216
3201	GO:0048002
3202	out_local_density_0.3_max
3203	GO:0005768
3204	GO:0030146
3205	GO:0019932
3206	GO:0033003
3207	secondary_structure_transition_NH_mean
3208	polarizability_distribution_N.0.0_mean
3209	GO:0030833
3210	GO:0008064
3211	GO:0030832
3212	GO:0030516
3213	GO:0006923
3214	GO:0006922
3215	GO:0033993
3216	GO:0051882
3217	GO:0051900
3218	GO:0015144
3219	GO:0051119
3220	GO:0015145
3221	GO:0015149
3222	GO:0005355
3223	GO:0009143
3224	GO:0043200
3225	GO:0016072
3226	GO:0006364
3227	GO:0042254
3228	GO:0042734
3229	GO:0008306
3230	GO:0031420
3231	GO:0008601
3232	GO:0003300
3233	GO:0050818
3234	GO:0007202
3235	GO:0001916
3236	GO:0007157
3237	GO:0010863

3238	GO:0014743
3239	GO:0019966
3240	GO:0010611
3241	GO:0014897
3242	GO:0002418
3243	GO:0005149
3244	GO:0015057
3245	GO:0070498
3246	GO:0015082
3247	GO:0010742
3248	GO:0090077
3249	GO:0035019
3250	GO:0050660
3251	GO:0019905
3252	GO:0042587
3253	GO:0031348
3254	GO:0043161
3255	GO:0010498
3256	GO:0030545
3257	GO:0033280
3258	GO:0008415
3259	GO:0016747
3260	GO:0050690
3261	GO:0019934
3262	GO:0042462
3263	GO:0015012
3264	GO:0032613
3265	GO:0032501
3266	GO:0044259
3267	GO:0046849
3268	GO:0044422
3269	GO:0005782
3270	GO:0016709
3271	GO:0031907
3272	GO:0042401
3273	GO:0042402
3274	GO:0016705
3275	GO:0044429
3276	GO:0007620
3277	topological_change_0.3_0.4
3278	GO:0030658
3279	GO:0016192
3280	GO:0051591
3281	GO:0016725
3282	GO:0045933
3283	GO:0043368
3284	GO:0043195

3285	GO:0008565
3286	GO:0017156
3287	GO:0021511
3288	GO:0035035
3289	GO:0021513
3290	GO:0021904
3291	GO:0043129
3292	GO:0048875
3293	GO:0009056
3294	GO:0016829
3295	GO:0032642
3296	GO:0008138
3297	GO:0030279
3298	GO:0051640
3299	GO:0014075
3300	GO:0032846
3301	secondary_structure_distribution_N.0.0_mean
3302	GO:0019886
3303	GO:0002667
3304	GO:0002913
3305	GO:0002669
3306	GO:0002249
3307	GO:0002870
3308	GO:0002495
3309	GO:0002478
3310	GO:0019884
3311	GO:0002911
3312	GO:0022029
3313	GO:0021885
3314	GO:0042159
3315	GO:0031639
3316	GO:0032319
3317	GO:0060443
3318	GO:0060056
3319	GO:0055072
3320	GO:0048471
3321	GO:0016811
3322	in_out_topological_variance
3323	GO:0048562
3324	GO:0010517
3325	GO:0048521
3326	GO:0010518
3327	GO:0050780
3328	GO:0060193
3329	GO:0008066
3330	GO:0060113
3331	GO:0035270

3332	GO:0001554
3333	GO:0007160
3334	GO:0050839
3335	GO:0007409
3336	GO:0050770
3337	GO:0007229
3338	GO:0006094
3339	GO:0008509
3340	GO:0005253
3341	GO:0055094
3342	GO:0042277
3343	GO:0030228
3344	GO:0003014
3345	in_locta_density_0.8_mean
3346	GO:0042304
3347	GO:0043422
3348	GO:0045088
3349	GO:0043473
3350	GO:0032720
3351	GO:0005525
3352	GO:0045923
3353	GO:0002886
3354	GO:0008076
3355	GO:0034705
3356	GO:0042033
3357	GO:0045073
3358	GO:0050755
3359	GO:0045080
3360	GO:0048541
3361	GO:0042448
3362	GO:0030850
3363	GO:0048537
3364	GO:0034708
3365	GO:0035097
3366	GO:0019825
3367	GO:0004601
3368	VanDerWaal_distribution_H.1.0_max
3369	GO:0004553
3370	GO:0042537
3371	GO:0030149
3372	GO:0016798
3373	GO:0016684
3374	polarizability_distribution_H.1.0_max
3375	GO:0006069
3376	GO:0006063
3377	GO:0004551
3378	GO:0009166

3379	GO:0006067
3380	GO:0042737
3381	GO:0005501
3382	GO:0019840
3383	GO:0008378
3384	GO:0004527
3385	GO:0019362
3386	GO:0006541
3387	GO:0004024
3388	GO:0044271
3389	GO:0035250
3390	GO:0019438
3391	GO:0004022
3392	GO:0046689
3393	GO:0004745
3394	GO:0047429
3395	GO:0008131
3396	GO:0051539
3397	GO:0032580
3398	GO:0006739
3399	GO:0034308
3400	GO:0016101
3401	GO:0019439
3402	GO:0016712
3403	GO:0001523
3404	GO:0019585
3405	GO:0043526
3406	VanDerWaal_distribution_P.0.75_mean
3407	GO:0050542
3408	GO:0050543
3409	GO:0042755
3410	GO:0071715
3411	GO:0032309
3412	in_clustering_mean
3413	GO:0017053
3414	GO:0048668
3415	GO:0016298
3416	GO:0001825
3417	GO:0048873
3418	GO:0007033
3419	GO:0007292
3420	polarity_distribution_H.0.5_mean
3421	GO:0050661
3422	GO:0042733
3423	GO:0030728
3424	GO:0001542
3425	GO:0042588

3426	GO:0045931
3427	GO:0031300
3428	GO:0032760
3429	GO:0009595
3430	GO:0004175
3431	GO:0008329
3432	GO:0042593
3433	GO:0033500
3434	GO:0005543
3435	GO:0016409
3436	topological_change_0.1_0.2
3437	out_locta_density_0.5_mean
3438	GO:0009914
3439	GO:0030072
3440	GO:0046879
3441	GO:0002790
3442	GO:0015833
3443	GO:0010906
3444	GO:0006109
3445	GO:0009954
3446	GO:0010675
3447	GO:0042439
3448	GO:0006810
3449	GO:0008294
3450	GO:0019935
3451	GO:0007187
3452	GO:0007188
3453	GO:0032045
3454	GO:0019933
3455	GO:0060089
3456	GO:0004871
3457	GO:0019961
3458	GO:0004904
3459	GO:0042095
3460	GO:0034393
3461	GO:0005125
3462	GO:0005143
3463	GO:0008343
3464	GO:0045072
3465	GO:0009605
3466	GO:0005173
3467	GO:0007411
3468	GO:0002032
3469	GO:0042474
3470	GO:0048640
3471	GO:0032729
3472	GO:0010661

3473	GO:0016070
3474	out_local_density_0.4_max
3475	GO:0044450
3476	GO:0001829
3477	GO:0016881
3478	GO:0042533
3479	GO:0042534
3480	GO:0031133
3481	GO:0042535
3482	GO:0032536
3483	GO:0070102
3484	GO:0008589
3485	GO:0006631
3486	secondary_structure_distribution_H.1.0_max
3487	GO:0042834
3488	GO:0043498
3489	GO:0045987
3490	GO:0014805
3491	GO:0007338
3492	GO:0008289
3493	GO:0006941
3494	GO:0045089
3495	in_local_density_0.5_max
3496	GO:0051926
3497	topological_change_0.7_0.8
3498	GO:0043167
3499	GO:0046872
3500	GO:0043169
3501	GO:0031253
3502	GO:0002467
3503	GO:0031649
3504	GO:0005900
3505	GO:0005896
3506	GO:0048861
3507	GO:0046880
3508	GO:0001880
3509	GO:0019955
3510	GO:0042074
3511	GO:0032002
3512	GO:0032230
3513	GO:0004896
3514	GO:0035254
3515	GO:0070120
3516	GO:0032276
3517	GO:0042036
3518	GO:0046882
3519	GO:0034332

3520	GO:0044062
3521	GO:0045161
3522	GO:0004897
3523	GO:0032924
3524	GO:0019976
3525	GO:0032277
3526	GO:0007220
3527	GO:0048712
3528	GO:0030514
3529	GO:0004924
3530	GO:0048185
3531	GO:0030510
3532	GO:0005127
3533	GO:0004911
3534	GO:0002673
3535	GO:0005739
3536	GO:0005743
3537	GO:0019866
3538	out_local_density_0.5_max
3539	GO:0021515
3540	GO:0020037
3541	GO:0016627
3542	GO:0016052
3543	GO:0008395
3544	GO:0046906
3545	GO:0005792
3546	GO:0042598
3547	GO:0006171
3548	GO:0006140
3549	GO:0015844
3550	GO:0046058
3551	GO:0004016
3552	GO:0051339
3553	GO:0009975
3554	GO:0051349
3555	GO:0016849
3556	GO:0030799
3557	GO:0030147
3558	GO:0031279
3559	GO:0009124
3560	GO:0009123
3561	GO:0030802
3562	GO:0030808
3563	GO:0031281
3564	GO:0030817
3565	GO:0009190
3566	GO:0030814

3567	GO:0009187
3568	GO:0055008
3569	GO:0060415
3570	GO:0006259
3571	GO:0006260
3572	GO:0031981
3573	GO:0008202
3574	AA_composition_K_mean
3575	GO:0032813
3576	in_locta_density_0.5_mean
3577	GO:0005694
3578	GO:0042359
3579	GO:0031201
3580	GO:0048193
3581	polarity_distribution_P.1.0_max
3582	GO:0042581
3583	GO:0006904
3584	GO:0005484
3585	GO:0001950
3586	GO:0042147
3587	GO:0048278
3588	GO:0016081
3589	GO:0006888
3590	GO:0007263
3591	GO:0008631
3592	GO:0048753
3593	GO:0004197
3594	GO:0006582
3595	GO:0008344
3596	hydrophobicity_distribution_N.0.25_mean
3597	GO:0012506
3598	GO:0000780
3599	GO:0071103
3600	GO:0000793
3601	GO:0000794
3602	GO:0006323
3603	GO:0022613
3604	GO:0045776
3605	GO:0016417
3606	GO:0004679
3607	GO:0002920
3608	GO:0009611
3609	GO:0031588
3610	GO:0051004
3611	GO:0016494
3612	GO:0046582
3613	GO:0030325

3614	GO:0031058
3615	GO:0031056
3616	GO:0030201
3617	GO:0044272
3618	GO:0009409
3619	GO:0031952
3620	GO:0043304
3621	GO:0046320
3622	GO:0043300
3623	GO:0033006
3624	GO:0003091
3625	GO:0050891
3626	GO:0001840
3627	GO:0001839
3628	GO:0010975
3629	GO:0040015
3630	GO:0048265
3631	GO:0055010
3632	GO:0003229
3633	GO:0003208
3634	GO:0003205
3635	GO:0003231
3636	GO:0003206
3637	GO:0030512
3638	GO:0030529
3639	GO:0022836
3640	GO:0015718
3641	GO:0015908
3642	GO:0042416
3643	GO:0010744
3644	in_degree_mean
3645	out_degree_mean
3646	GO:0016742
3647	GO:0004372
3648	in_local_density_0.7_max
3649	GO:0060420
3650	GO:0055024
3651	GO:0060271
3652	GO:0060043
3653	GO:0055021
3654	GO:0071339
3655	GO:0030903
3656	GO:0032925
3657	GO:0042384
3658	GO:0050435
3659	GO:0050731

3660	GO:0048247
3661	GO:0034394
3662	GO:0048570
3663	GO:0042789
3664	GO:0006953
3665	GO:0033628
3666	in_degree_median
3667	GO:0042578
3668	GO:0045342
3669	GO:0045346
3670	GO:0033631
3671	GO:0031256
3672	secondary_structure_distribution_H.0.75_mean
3673	GO:0043518
3674	GO:0005654
3675	GO:0005773
3676	GO:0005996
3677	GO:0005777
3678	GO:0006090
3679	GO:0019318
3680	GO:0042579
3681	GO:0019319
3682	GO:0048066
3683	GO:0002446
3684	GO:0008582
3685	GO:0030178
3686	GO:0009898
3687	out_degree_correlation_max
3688	GO:0006633
3689	GO:0016208
3690	GO:0008168
3691	GO:0006558
3692	GO:0046121
3693	GO:0046514
3694	GO:0018065
3695	GO:0006518
3696	GO:0009404
3697	GO:0046466
3698	GO:0015925
3699	GO:0070566
3700	GO:0016098
3701	GO:0006085
3702	GO:0006572
3703	GO:0042440
3704	GO:0042558
3705	GO:0042420
3706	GO:0042424

3707	GO:0034313
3708	GO:0030203
3709	GO:0042773
3710	GO:0042775
3711	GO:0034754
3712	GO:0006208
3713	polarizability_distribution_P.1.0_max
3714	GO:0004602
3715	GO:0019859
3716	GO:0019614
3717	GO:0019674
3718	GO:0019377
3719	GO:0006022
3720	GO:0004528
3721	GO:0006210
3722	GO:0006559
3723	GO:0000097
3724	GO:0016624
3725	GO:0009112
3726	GO:0009074
3727	GO:0016840
3728	GO:0009116
3729	GO:0016741
3730	GO:0009100
3731	GO:0006749
3732	GO:0008299
3733	GO:0009069
3734	GO:0006734
3735	GO:0009068
3736	GO:0009120
3737	GO:0009164
3738	GO:0016814
3739	GO:0006892
3740	GO:0005874
3741	GO:0008641
3742	GO:0007018
3743	GO:0051225
3744	GO:0005885
3745	GO:0007017
3746	GO:0031301
3747	GO:0042445
3748	GO:0032602
3749	GO:0005001
3750	GO:0014069
3751	GO:0019198
3752	GO:0005080
3753	GO:0000209

3754	GO:0007156
3755	GO:0045087
3756	GO:0043120
3757	GO:0007215
3758	GO:0008015
3759	GO:0002714
3760	GO:0002891
3761	GO:0003013
3762	GO:0051651
3763	GO:0032769
3764	GO:0000096
3765	GO:0045454
3766	GO:0005740
3767	GO:0031966
3768	GO:0008207
3769	GO:0019637
3770	GO:0006644
3771	GO:0051234
3772	GO:0031965
3773	GO:0006641
3774	GO:0010243
3775	GO:0015697
3776	GO:0070229
3777	GO:0070228
3778	GO:0006101
3779	GO:0006600
3780	GO:0006599
3781	GO:0048038
3782	GO:0009154
3783	GO:0009261
3784	GO:0002524
3785	GO:0002883
3786	GO:0002449
3787	GO:0014820
3788	GO:0014848
3789	GO:0002864
3790	GO:0014832
3791	GO:0045879
3792	GO:0002861
3793	GO:0014824
3794	GO:0007223
3795	GO:0048484
3796	GO:0016055
3797	GO:0002438
3798	GO:0070469
3799	GO:0002791
3800	GO:0090276

3801	GO:0090087
3802	GO:0050796
3803	GO:0051262
3804	GO:0004725
3805	GO:0008328
3806	GO:0019865
3807	GO:0002220
3808	GO:0055102
3809	GO:0005184
3810	GO:0004970
3811	out_local_density_0.6_max
3812	out_degree_median
3813	GO:0005903
3814	in_degree_correlation_max
3815	GO:0030133
3816	GO:0001573
3817	GO:0043403
3818	polarizability_distribution_H.0.25_mean
3819	VanDerWaal_distribution_H.0.25_mean
3820	GO:0004622
3821	GO:0004091
3822	VanDerWaal_distribution_H.0.75_mean
3823	polarizability_distribution_H.0.75_mean
3824	GO:0031670
3825	GO:0030864
3826	GO:0007589
3827	GO:0007585
3828	GO:0048306
3829	GO:0046930
3830	GO:0006508
3831	GO:0000049
3832	hydrophobicity_distribution_H.1.0_max
3833	GO:0005496
3834	GO:0006663
3835	GO:0046469
3836	GO:0015732
3837	GO:0032892
3838	GO:0032303
3839	GO:0032306
3840	GO:0032308
3841	GO:0032310
3842	GO:0032305
3843	GO:0004623
3844	GO:0047498
3845	GO:0016071
3846	GO:0045026
3847	GO:0005504

3848	GO:0044275
3849	GO:0033540
3850	GO:0022904
3851	GO:0019432
3852	GO:0046148
3853	GO:0019841
3854	GO:0030258
3855	GO:0019953
3856	GO:0044242
3857	GO:0007628
3858	GO:0000299
3859	GO:0042461
3860	GO:0005720
3861	GO:0001101
3862	GO:0007586
3863	GO:0055117
3864	GO:0045341
3865	GO:0042612
3866	GO:0032393
3867	GO:0042611
3868	GO:0045343
3869	GO:0002474
3870	GO:0006954
3871	GO:0019882
3872	GO:0001505
3873	GO:0031968
3874	GO:0019867
3875	GO:0006281
3876	GO:0001750
3877	GO:0030140
3878	GO:0010887
3879	secondary_structure_distribution_N.0.25_mean
3880	GO:0042165
3881	GO:0031929
3882	GO:0032279
3883	GO:0016327
3884	GO:0030193
3885	GO:0014821
3886	GO:0016338
3887	GO:0060042
3888	GO:0031398
3889	GO:0043296
3890	GO:0005923
3891	GO:0070160
3892	GO:0042730
3893	GO:0016817
3894	GO:0009062

3895	GO:0009374
3896	GO:0046364
3897	GO:0044438
3898	GO:0044439
3899	GO:0016877
3900	GO:0046165
3901	GO:0008374
3902	GO:0006635
3903	GO:0042288
3904	GO:0045851
3905	GO:0051452
3906	GO:0005625
3907	GO:0046942
3908	GO:0015849
3909	GO:0070011
3910	GO:0034446
3911	GO:0005164
3912	GO:0042153
3913	GO:0044238
3914	GO:0031967
3915	GO:0031975
3916	GO:0007605
3917	GO:0050885
3918	GO:0019370
3919	GO:0043450
3920	GO:0051380
3921	GO:0004952
3922	GO:0003321
3923	GO:0000776
3924	GO:0051378
3925	GO:0030594
3926	GO:0004981
3927	GO:0034331
3928	GO:0004935
3929	GO:0030432
3930	GO:0003678
3931	GO:0015464
3932	GO:0042271
3933	GO:0005588
3934	GO:0005583
3935	GO:0007512
3936	GO:0004993
3937	GO:0008227
3938	GO:0000151
3939	GO:0031461
3940	GO:0045217
3941	GO:0004939

3942	GO:0030819
3943	GO:0016907
3944	GO:0007207
3945	GO:0051954
3946	GO:0001993
3947	GO:0007029
3948	GO:0001976
3949	GO:0031396
3950	GO:0030816
3951	GO:0048665
3952	GO:0005244
3953	GO:0022832
3954	GO:0021520
3955	GO:0021521
3956	GO:0021514
3957	GO:0034702
3958	GO:0060579
3959	GO:0042491
3960	GO:0007224
3961	GO:0030878
3962	GO:0006986
3963	GO:0032637
3964	GO:0005770
3965	GO:0042752
3966	GO:0042246
3967	GO:0002027
3968	GO:0001783
3969	GO:0046521
3970	GO:0016780
3971	GO:0046457
3972	GO:0001516
3973	GO:0030247
3974	GO:0001871
3975	in_degree_variance
3976	GO:0032934
3977	secondary_structure_distribution_P.1.0_mean
3978	GO:0051213
3979	GO:0016701
3980	GO:0016702
3981	GO:0019861
3982	GO:0008234
3983	GO:0003682
3984	GO:0042743
3985	GO:0031225
3986	GO:0034440
3987	GO:0019395
3988	GO:0000038

3989	GO:0042220
3990	GO:0014073
3991	GO:0043233
3992	GO:0034105
3993	hydrophobicity_distribution_P.0.75_mean
3994	GO:0002029
3995	GO:0032606
3996	out_degree_max
3997	GO:0022401
3998	GO:0055080
3999	GO:0023058
4000	GO:0002444
4001	GO:0015631
4002	GO:0043648
4003	GO:0016857
4004	GO:0006547
4005	GO:0045254
4006	GO:0009113
4007	GO:0006534
4008	GO:0032259
4009	GO:0016763
4010	GO:0009130
4011	GO:0016861
4012	GO:0009311
4013	GO:0046835
4014	GO:0009119
4015	GO:0009129
4016	GO:0009220
4017	GO:0009156
4018	GO:0016832
4019	GO:0006548
4020	GO:0016411
4021	GO:0016405
4022	GO:0016646
4023	GO:0009163
4024	GO:0048531
4025	GO:0070988
4026	GO:0042455
4027	GO:0006752
4028	GO:0046129
4029	GO:0017085
4030	GO:0030176
4031	GO:0042451
4032	GO:0046125
4033	GO:0046398
4034	GO:0006740
4035	GO:0018130

4036	GO:0016868
4037	GO:0006027
4038	GO:0006776
4039	GO:0006047
4040	GO:0006106
4041	GO:0042364
4042	GO:0006026
4043	GO:0019321
4044	GO:0004614
4045	GO:0019852
4046	GO:0043413
4047	GO:0070085
4048	GO:0009822
4049	GO:0006479
4050	GO:0016884
4051	GO:0016878
4052	GO:0009101
4053	GO:0043414
4054	GO:0009077
4055	GO:0006486
4056	GO:0034404
4057	GO:0046479
4058	GO:0034654
4059	GO:0070989
4060	GO:0009075
4061	GO:0008213
4062	GO:0009071
4063	GO:0006086
4064	GO:0017040
4065	GO:0019239
4066	GO:0070325
4067	GO:0022838
4068	GO:0022803
4069	GO:0015267
4070	GO:0005216
4071	GO:0001875
4072	GO:0015807
4073	GO:0050750
4074	GO:0006289
4075	GO:0030672
4076	GO:0044248
4077	GO:0006105
4078	GO:0004385
4079	GO:0007168
4080	GO:0008211
4081	GO:0030553
4082	GO:0030433

4083	GO:0015931
4084	GO:0050873
4085	GO:0051351
4086	GO:0051352
4087	GO:0048013
4088	GO:0031397
4089	GO:0031145
4090	GO:0007218
4091	GO:0002755
4092	GO:0034121
4093	GO:0051437
4094	GO:0004704
4095	GO:0051436
4096	GO:0051444
4097	GO:0051439
4098	GO:0051443
4099	GO:0002281
4100	GO:0052033
4101	GO:0052169
4102	GO:0002282
4103	GO:0052167
4104	GO:0002020
4105	GO:0052257
4106	GO:0052255
4107	GO:0052166
4108	GO:0001774
4109	GO:0003708
4110	GO:0052555
4111	GO:0052509
4112	GO:0052553
4113	GO:0052552
4114	GO:0052510
4115	GO:0052031
4116	GO:0052556
4117	GO:0052305
4118	GO:0052308
4119	GO:0052306
4120	GO:0032757
4121	GO:0031113
4122	GO:0031112
4123	GO:0005606
4124	GO:0005604
4125	GO:0005581
4126	GO:0005605
4127	GO:0031116
4128	GO:0043121
4129	GO:0030198

4130	GO:0030935
4131	GO:0046785
4132	GO:0043256
4133	GO:0044420
4134	GO:0005587
4135	GO:0043178
4136	GO:0046461
4137	GO:0019433
4138	GO:0019203
4139	GO:0046464
4140	GO:0044269
4141	GO:0016885
4142	GO:0021549
4143	GO:0021510
4144	GO:0019217
4145	GO:0010565
4146	GO:0030166
4147	in_degree_correlation_mean
4148	GO:0000287
4149	GO:0016874
4150	GO:0006516
4151	GO:0006705
4152	GO:0008212
4153	GO:0032342
4154	GO:0042987
4155	GO:0070646
4156	GO:0032341
4157	GO:0005681
4158	GO:0006812
4159	GO:0006811
4160	GO:0019320
4161	GO:0006091
4162	GO:0016812
4163	GO:0046365
4164	GO:0046504
4165	GO:0046912
4166	GO:0002054
4167	GO:0046164
4168	GO:0046460
4169	GO:0016634
4170	GO:0006096
4171	GO:0046463
4172	GO:0016607
4173	GO:0010008
4174	GO:0044440
4175	GO:0033555
4176	GO:0002675

4177	GO:0008217
4178	GO:0042310
4179	GO:0010578
4180	GO:0045762
4181	GO:0007189
4182	GO:0007190
4183	GO:0010579
4184	GO:0031645
4185	GO:0051970
4186	GO:0050805
4187	GO:0007205
4188	GO:0060137
4189	GO:0020027
4190	GO:0048770
4191	GO:0042470
4192	GO:0015291
4193	GO:0008308
4194	GO:0005267
4195	GO:0005793
4196	GO:0043621
4197	GO:0030299
4198	GO:0033596
4199	GO:0005031
4200	GO:0005035
4201	GO:0005881
4202	GO:0042541
4203	GO:0033089
4204	GO:0006584
4205	GO:0018958
4206	GO:0070013
4207	GO:0009712
4208	in_local_density_0.4_max
4209	GO:0034311
4210	GO:0030246
4211	GO:0051928
4212	GO:0004867
4213	GO:0005741
4214	GO:0043227
4215	GO:0043231
4216	GO:0021795
4217	GO:0055066
4218	GO:0016020
4219	GO:0030136
4220	VanDerWaal_distribution_P.1.0_mean
4221	in_in_topological_mean
4222	out_degree_variance
4223	GO:0046339

4224	GO:0008081
4225	GO:0009395
4226	VanDerWaal_distribution_N.0.5_mean
4227	out_degree_correlation_mean
4228	GO:0044428
4229	GO:0031974
4230	GO:0034470
4231	GO:0001660
4232	GO:0032225
4233	GO:0045347
4234	GO:0002064
4235	GO:0031282
4236	GO:0015695
4237	GO:0051001
4238	GO:0044437
4239	GO:0055088
4240	GO:0016421
4241	GO:0004075
4242	GO:0003987
4243	GO:0008375
4244	GO:0030173
4245	GO:0031228
4246	GO:0051233
4247	GO:0014866
4248	GO:0043088
4249	GO:0015297
4250	GO:0007091
4251	GO:0015300
4252	GO:0032489
4253	GO:0019201
4254	GO:0045744
4255	GO:0004726
4256	GO:0016209
4257	GO:0006820
4258	GO:0007618
4259	GO:0044446
4260	GO:0042742
4261	GO:0005815
4262	GO:0017111
4263	GO:0016818
4264	GO:0016462
4265	GO:0015914
4266	GO:0006111
4267	GO:0045907
4268	GO:0032561
4269	GO:0019001
4270	GO:0052564

4271	GO:0052572
4272	GO:0006457
4273	GO:0006412
4274	GO:0016740
4275	GO:0042287
4276	GO:0006112
4277	GO:0002903
4278	GO:0002902
4279	GO:0046476
4280	GO:0050857
4281	GO:0004117
4282	GO:0005977
4283	GO:0019835
4284	GO:0031224
4285	GO:0045354
4286	GO:0007602
4287	GO:0014049
4288	GO:0005615
4289	GO:0004689
4290	GO:0051019
4291	GO:0008135
4292	GO:0033691
4293	GO:0051919
4294	GO:0005964
4295	GO:0004872
4296	GO:0032607
4297	GO:0045078
4298	GO:0003743
4299	GO:0005126
4300	GO:0002504
4301	GO:0000502
4302	GO:0009583
4303	GO:0016021
4304	GO:0008035
4305	GO:0008432
4306	GO:0007413
4307	GO:0032395
4308	GO:0045349
4309	GO:0042613
4310	GO:0032647
4311	GO:0046486
4312	GO:0046473
4313	GO:0016879
4314	GO:0045909
4315	GO:0003018
4316	GO:0016775
4317	GO:0016416

4318	GO:0016406
4319	GO:0004095
4320	GO:0001702
4321	GO:0043531
4322	GO:0046688
4323	GO:0015850
4324	in_degree_max
4325	GO:0008354
4326	GO:0005635
4327	GO:0018205
4328	GO:0046887
4329	GO:0007595
4330	GO:0045329
4331	GO:0048029
4332	GO:0005536
4333	GO:0043449
4334	GO:0006007
4335	GO:0000323
4336	GO:0005764
4337	GO:0006691
4338	GO:0046470
4339	GO:0019200
4340	GO:0015980
4341	GO:0008203
4342	GO:0016125
4343	GO:0043274
4344	GO:0051117
4345	out_degree_correlation_variance
4346	GO:0006107
4347	GO:0043620
4348	GO:0042393
4349	GO:0060405
4350	GO:0006700
4351	GO:0006029
4352	GO:0032508
4353	GO:0048487
4354	GO:0051340
4355	GO:0070979
4356	GO:0006270
4357	GO:0032982
4358	GO:0030660
4359	GO:0000777
4360	GO:0006268
4361	GO:0000808
4362	GO:0008091
4363	GO:0007062
4364	GO:0000152

4365	GO:0005680
4366	GO:0005664
4367	GO:0050811
4368	GO:0003688
4369	GO:0032392
4370	GO:0051438
4371	GO:0051322
4372	GO:0000090
4373	out_out_topological_variance
4374	GO:0042354
4375	GO:0009219
4376	GO:0070569
4377	GO:0009218
4378	GO:0042572
4379	GO:0015036
4380	GO:0009448
4381	GO:0042278
4382	GO:0006555
4383	GO:0009264
4384	GO:0003836
4385	GO:0019238
4386	GO:0007339
4387	GO:0016291
4388	GO:0031227
4389	GO:0006544
4390	GO:0050667
4391	GO:0046474
4392	GO:0009394
4393	GO:0006533
4394	GO:0008195
4395	GO:0046487
4396	GO:0006695
4397	GO:0008499
4398	GO:0009988
4399	GO:0009060
4400	GO:0009312
4401	GO:0008373
4402	GO:0046477
4403	GO:0046349
4404	GO:0006531
4405	GO:0006023
4406	GO:0006024
4407	GO:0046128
4408	GO:0006098
4409	GO:0006011
4410	GO:0046653
4411	GO:0016645

4412	GO:0006760
4413	GO:0035036
4414	GO:0009070
4415	GO:0009173
4416	GO:0016748
4417	GO:0016841
4418	GO:0016801
4419	GO:0033865
4420	GO:0050897
4421	GO:0046033
4422	GO:0006084
4423	GO:0009161
4424	GO:0006004
4425	GO:0046049
4426	GO:0003854
4427	GO:0006222
4428	GO:0006206
4429	GO:0016782
4430	GO:0005984
4431	GO:0006207
4432	GO:0019856
4433	GO:0016833
4434	GO:0009185
4435	GO:0042392
4436	polarity_distribution_N.1.0_max
4437	GO:0004396
4438	GO:0009174
4439	GO:0050746
4440	GO:0043462
4441	GO:0005507
4442	GO:0030890
4443	GO:0006195
4444	GO:0019992
4445	GO:0006650
4446	GO:0031404
4447	GO:0051181
4448	GO:0004177
4449	GO:0005778
4450	GO:0004467
4451	GO:0031903
4452	GO:0015645
4453	GO:0070372
4454	GO:0070371
4455	GO:0070374
4456	GO:0045136
4457	GO:0048566
4458	GO:0005758

4459	GO:0050830
4460	GO:0043010
4461	GO:0008209
4462	GO:0030641
4463	GO:0051453
4464	GO:0050872
4465	GO:0008277
4466	GO:0021602
4467	GO:0022627
4468	GO:0014051
4469	GO:0022626
4470	GO:0008038
4471	GO:0051605
4472	GO:0002042
4473	GO:0046873
4474	GO:0019724
4475	GO:0015935
4476	GO:0033279
4477	GO:0042813
4478	GO:0021631
4479	GO:0008236
4480	GO:0010872
4481	GO:0014044
4482	GO:0010873
4483	GO:0004252
4484	GO:0004953
4485	GO:0016064
4486	GO:0001967
4487	GO:0015812
4488	GO:0007164
4489	GO:0051604
4490	GO:0051084
4491	GO:0004954
4492	GO:0021554
4493	GO:0001738
4494	GO:0008331
4495	GO:0005840
4496	GO:0016485
4497	GO:0004718
4498	GO:0006458
4499	GO:0005262
4500	GO:0005261
4501	GO:0004955
4502	GO:0001736
4503	GO:0051085
4504	GO:0017171
4505	GO:0004926

4506	GO:0015270
4507	GO:0006677
4508	GO:0050892
4509	GO:0007567
4510	GO:0006006
4511	GO:0016051
4512	GO:0009262
4513	GO:0005774
4514	GO:0009132
4515	GO:0016728
4516	GO:0009263
4517	GO:0004748
4518	GO:0016776
4519	GO:0019205
4520	GO:0005044
4521	GO:0043176
4522	GO:0015698
4523	GO:0008233
4524	GO:0042116
4525	GO:0042745
4526	GO:0006163
4527	GO:0006164
4528	GO:0048512
4529	GO:0009743
4530	GO:0044237
4531	polarity_distribution_N.1.0_mean
4532	in_degree_correlation_variance
4533	GO:0007588
4534	GO:0008654
4535	GO:0033057
4536	GO:0006396
4537	GO:0009247
4538	GO:0005104
4539	GO:0007418
4540	GO:0008543
4541	GO:0005245
4542	GO:0008332
4543	GO:0001892
4544	GO:0005891
4545	GO:0033235
4546	GO:0006884
4547	GO:0034704
4548	GO:0005111
4549	GO:0021938
4550	GO:0031503
4551	GO:0033233
4552	GO:0019789

4553	GO:0016925
4554	GO:0022843
4555	GO:0008330
4556	GO:0021937
4557	GO:0045665
4558	GO:0021910
4559	GO:0034703
4560	GO:0005113
4561	GO:0001669
4562	GO:0031953
4563	in_in_topological_variance
4564	GO:0034614
4565	GO:0030145
4566	GO:0005640
4567	GO:0044264
4568	GO:0002031
4569	GO:0006639
4570	GO:0006638
4571	GO:0044042
4572	GO:0006662
4573	GO:0006073
4574	GO:0006325
4575	GO:0050432
4576	GO:0051937
4577	GO:0050433
4578	GO:0016308
4579	GO:0007625
4580	GO:0016309
4581	GO:0035240
4582	GO:0042166
4583	GO:0005876
4584	GO:0060292
4585	GO:0003073
4586	GO:0031579
4587	GO:0016568
4588	GO:0016307
4589	GO:0008094
4590	GO:0018904
4591	GO:0006914
4592	GO:0046483
4593	GO:0009110
4594	GO:0034599
4595	GO:0055093
4596	GO:0016859
4597	GO:0007623
4598	GO:0007274
4599	GO:0043266

4600	GO:0009250
4601	GO:0090181
4602	GO:0005978
4603	GO:0045540
4604	GO:0006103
4605	GO:0002793
4606	GO:0090277
4607	GO:0009146
4608	GO:0042069
4609	GO:0000228
4610	GO:0044454
4611	GO:0045019
4612	GO:0042053
4613	GO:0031970
4614	GO:0043587
4615	GO:0021536
4616	GO:0008080
4617	GO:0032024
4618	GO:0009165
4619	GO:0007622
4620	GO:0048857
4621	GO:0006014
4622	GO:0009072
4623	GO:0016615
4624	GO:0005976
4625	GO:0033539
4626	GO:0006108
4627	GO:0016713
4628	GO:0045017
4629	GO:0046503
4630	GO:0009593
4631	GO:0016570
4632	GO:0016569
4633	GO:0005776
4634	GO:0005790
4635	GO:0006333
4636	GO:0009744
4637	GO:0034285
4638	GO:0031513
4639	GO:0010889
4640	GO:0034383
4641	GO:0006958
4642	GO:0060228
4643	GO:0035235
4644	GO:0016199
4645	GO:0004886
4646	GO:0033700

4647	GO:0006959
4648	GO:0034380
4649	GO:0002541
4650	GO:0004866
4651	GO:0016198
4652	GO:0006956
4653	GO:0032488
4654	GO:0002526
4655	GO:0005042
4656	GO:0005254
4657	GO:0002455
4658	GO:0005217
4659	GO:0017162
4660	GO:0017127
4661	GO:0006957
4662	GO:0030414
4663	GO:0016529
4664	GO:0016528
4665	GO:0001990
4666	GO:0033189
4667	GO:0055086
4668	GO:0005798
4669	GO:0040020
4670	GO:0043954
4671	GO:0051445
4672	GO:0010460
4673	GO:0006200
4674	GO:0006261
4675	GO:0000731
4676	GO:0016591
4677	GO:0043596
4678	GO:0009186
4679	GO:0009200
4680	in_local_density_0.3_max
4681	GO:0030894
4682	GO:0005658
4683	GO:0000421
4684	GO:0043601
4685	GO:0016799
4686	hydrophobicity_distribution_P.1.0_max
4687	GO:0035005
4688	GO:0042379
4689	GO:0002087
4690	GO:0033549
4691	GO:0070471
4692	GO:0033829
4693	GO:0042923

4694	GO:0070472
4695	GO:0004995
4696	GO:0070474
4697	GO:0033017
4698	GO:0005388
4699	GO:0001992
4700	GO:0044065
4701	GO:0005078
4702	GO:0003078
4703	GO:0005112
4704	GO:0005000
4705	GO:0004974
4706	GO:0001664
4707	GO:0015085
4708	GO:0019956
4709	GO:0019957
4710	GO:0001637
4711	GO:0007386
4712	GO:0008188
4713	GO:0008009
4714	GO:0016493
4715	GO:0004937
4716	GO:0051024
4717	GO:0043576
4718	GO:0007217
4719	GO:0004936
4720	GO:0007219
4721	GO:0017017
4722	GO:0001614
4723	GO:0016502
4724	GO:0004950
4725	GO:0004969
4726	GO:0022600
4727	GO:0019369
4728	GO:0042312
4729	GO:0004383
4730	GO:0046068
4731	GO:0006182
4732	GO:0040029
4733	GO:0030135
4734	GO:0005529
4735	GO:0005765
4736	out_local_density_0.7_max
4737	GO:0034645
4738	GO:0009059
4739	GO:0035150
4740	GO:0050880

4741	GO:0006839
4742	GO:0042301
4743	GO:0006869
4744	GO:0050906
4745	GO:0009203
4746	GO:0009207
4747	GO:0009566
4748	GO:0051028
4749	GO:0010817
4750	GO:0042744
4751	GO:0017169
4752	GO:0070301
4753	GO:0031403
4754	GO:0004143
4755	GO:0050654
4756	GO:0045745
4757	GO:0000779
4758	GO:0006826
4759	GO:0016722
4760	GO:0000041
4761	GO:0008088
4762	GO:0055085
4763	GO:0021983
4764	GO:0010885
4765	GO:0060024
4766	GO:0006144
4767	GO:0005542
4768	GO:0046027
4769	GO:0042355
4770	GO:0004659
4771	GO:0009127
4772	GO:0008417
4773	VanDerWaal_distribution_P.1.0_max
4774	GO:0008380
4775	GO:0009396
4776	GO:0045333
4777	GO:0008253
4778	GO:0008171
4779	GO:0008252
4780	GO:0008199
4781	GO:0046467
4782	GO:0009265
4783	GO:0006221
4784	GO:0006220
4785	GO:0006244
4786	GO:0043094
4787	GO:0019317

4788	GO:0046100
4789	GO:0043043
4790	GO:0004047
4791	GO:0006213
4792	GO:0046085
4793	GO:0004739
4794	GO:0004738
4795	GO:0006775
4796	GO:0042904
4797	GO:0030062
4798	GO:0005746
4799	GO:0016093
4800	GO:0030259
4801	GO:0046356
4802	GO:0030148
4803	GO:0015936
4804	GO:0006750
4805	GO:0003841
4806	GO:0046174
4807	GO:0046131
4808	GO:0003840
4809	GO:0019695
4810	GO:0016090
4811	GO:0006012
4812	GO:0006537
4813	GO:0009064
4814	GO:0015929
4815	GO:0016714
4816	GO:0046950
4817	GO:0009086
4818	GO:0051187
4819	GO:0006563
4820	GO:0046502
4821	GO:0009109
4822	GO:0046519
4823	GO:0006546
4824	GO:0009083
4825	GO:0009084
4826	GO:0009191
4827	GO:0006672
4828	GO:0006551
4829	GO:0009168
4830	GO:0006099
4831	GO:0035238
4832	GO:0009167
4833	GO:0004311
4834	GO:0045239

4835	GO:0019136
4836	GO:0006687
4837	GO:0016803
4838	GO:0019206
4839	GO:0042905
4840	GO:0004332
4841	GO:0016778
4842	GO:0009259
4843	GO:0006565
4844	GO:0009260
4845	GO:0006664
4846	GO:0009126
4847	GO:0009133
4848	GO:0016755
4849	GO:0009134
4850	GO:0004364
4851	GO:0016765
4852	GO:0006643
4853	GO:0006665
4854	GO:0009268
4855	GO:0008034
4856	GO:0033762
4857	GO:0034637
4858	GO:0008238
4859	GO:0050916
4860	GO:0060192
4861	GO:0021988
4862	GO:0004875
4863	GO:0043020
4864	GO:0021891
4865	GO:0070508
4866	GO:0005003
4867	GO:0045350
4868	GO:0050917
4869	GO:0034123
4870	GO:0004972
4871	GO:0005005
4872	GO:0021889
4873	GO:0021772
4874	GO:0090208
4875	GO:0016175
4876	GO:0005579
4877	GO:0035376
4878	GO:0031290
4879	GO:0050909
4880	GO:0035382
4881	GO:0070653

4882	GO:0008048
4883	GO:0090207
4884	GO:0045357
4885	GO:0010851
4886	GO:0019864
4887	GO:0010853
4888	GO:0046875
4889	GO:0022624
4890	GO:0008538
4891	GO:0001867
4892	GO:0015245
4893	GO:0017146
4894	GO:0008046
4895	GO:0045359
4896	GO:0030250
4897	GO:0048843
4898	GO:0010860
4899	GO:0030249
4900	GO:0045499
4901	GO:0048846
4902	GO:0007598
4903	GO:0048841
4904	GO:0030517
4905	GO:0017154
4906	GO:0010903
4907	GO:0034366
4908	GO:0010901
4909	GO:0008537
4910	GO:0010896
4911	GO:0008527
4912	GO:0001848
4913	GO:0016602
4914	GO:0022900
4915	GO:0050953
4916	GO:0007601
4917	GO:0006753
4918	GO:0009117
4919	GO:0015248
4920	GO:0031405
4921	GO:0050665
4922	GO:0048593
4923	polarity_distribution_H.0.75_mean
4924	GO:0045721
4925	GO:0034369
4926	GO:0034367
4927	GO:0034368
4928	GO:0042423

4929	GO:0006636
4930	GO:0046456
4931	GO:0006690
4932	GO:0006694
4933	GO:0033559
4934	GO:0048384
4935	GO:0045814
4936	GO:0051276
4937	GO:0019213
4938	GO:0006342
4939	GO:0005643
4940	GO:0006000
4941	GO:0006656
4942	GO:0042597
4943	GO:0006570
4944	GO:0006002
4945	GO:0030288
4946	GO:0046527
4947	GO:0005945
4948	GO:0070061
4949	GO:0070095
4950	GO:0001961
4951	GO:0034451
4952	GO:0000272
4953	GO:0009251
4954	GO:0034379
4955	GO:0019359
4956	GO:0070542
4957	GO:0004331
4958	GO:0046039
4959	GO:0046459
4960	GO:0004470
4961	GO:0019693
4962	GO:0016594
4963	GO:0000036
4964	GO:0008443
4965	GO:0009125
4966	GO:0044247
4967	GO:0035251
4968	GO:0003872
4969	GO:0030388
4970	GO:0031402
4971	GO:0045187
4972	GO:0042749
4973	GO:0022410
4974	GO:0015485
4975	GO:0050802

4976	GO:0005791
4977	GO:0046500
4978	GO:0008172
4979	GO:0048515
4980	GO:0007286
4981	GO:0008021
4982	GO:0005657
4983	GO:0030730
4984	GO:0000002
4985	GO:0005875
4986	GO:0006654
4987	GO:0006521
4988	GO:0009791
4989	GO:0006692
4990	GO:0003044
4991	GO:0030204
4992	GO:0006693
4993	GO:0048821
4994	GO:0048148
4995	GO:0046498
4996	GO:0010522
4997	GO:0044425
4998	GO:0046513
4999	GO:0006414
5000	GO:0006527
5001	GO:0034185
5002	GO:0044306
5003	GO:0043679
5004	GO:0008144
5005	GO:0006352
5006	GO:0030057
5007	GO:0006367
5008	GO:0006403
5009	GO:0021952
5010	GO:0050658
5011	GO:0051236
5012	GO:0050657
5013	GO:0030823
5014	GO:0030826
5015	GO:0007422
5016	GO:0006688
5017	GO:0019751
5018	GO:0045104
5019	GO:0071514
5020	GO:0060052
5021	GO:0045103
5022	GO:0045109

5023	GO:0005883
5024	GO:0060053
5025	GO:0006349
5026	GO:0006821
5027	GO:0045110
5028	GO:0031018
5029	GO:0001963
5030	in_clustering_variance
5031	GO:0017158
5032	GO:0034381
5033	GO:0019098
5034	GO:0007617
5035	GO:0010876
5036	GO:0030534
5037	GO:0043168
5038	GO:0008237
5039	GO:0008270
5040	GO:0045777
5041	GO:0015874
5042	GO:0042605
5043	GO:0014061
5044	GO:0015075
5045	GO:0042711
5046	GO:0051766
5047	GO:0003071
5048	GO:0046520
5049	GO:0070567
5050	GO:0034593
5051	GO:0048243
5052	GO:0008440
5053	GO:0015662
5054	GO:0034595
5055	GO:0002636
5056	GO:0046030
5057	GO:0004437
5058	GO:0060746
5059	GO:0004445
5060	GO:0002634
5061	GO:0006020
5062	GO:0003084
5063	GO:0004439
5064	GO:0010288
5065	GO:0006813
5066	GO:0050650
5067	GO:0045980
5068	GO:0016234
5069	GO:0004339

5070	GO:0034341
5071	GO:0015926
5072	GO:0006139
5073	GO:0051705
5074	GO:0006807
5075	GO:0006072
5076	GO:0006646
5077	GO:0003995
5078	GO:0050997
5079	GO:0006580
5080	GO:0032608
5081	GO:0032648
5082	GO:0032479
5083	GO:0016717
5084	GO:0019322
5085	GO:0042157
5086	GO:0034235
5087	GO:0002686
5088	GO:0046337
5089	GO:0032481
5090	GO:0046335
5091	GO:0047372
5092	GO:0001659
5093	GO:0021761
5094	GO:0045912
5095	GO:0010677
5096	GO:0015721
5097	GO:0031418
5098	GO:0043691
5099	GO:0055092
5100	GO:0042632
5101	GO:0051606
5102	GO:0033483
5103	GO:0030384
5104	GO:0007140
5105	GO:0005319
5106	GO:0010878
5107	GO:0016791
5108	GO:0050905
5109	GO:0010181
5110	GO:0045986
5111	GO:0030810
5112	GO:0030801
5113	GO:0090075
5114	GO:0030828
5115	GO:0043206
5116	GO:0030825

5117	GO:0060087
5118	GO:0030804
5119	GO:0045981
5120	GO:0070874
5121	GO:0005813
5122	GO:0016410
5123	GO:0006183
5124	GO:0006188
5125	GO:0006177
5126	GO:0016887
5127	GO:0019627
5128	GO:0006189
5129	GO:0050327
5130	GO:0046051
5131	GO:0019363
5132	GO:0019374
5133	GO:0019405
5134	GO:0019441
5135	GO:0004769
5136	GO:0006167
5137	GO:0004774
5138	GO:0018211
5139	GO:0046037
5140	GO:0003831
5141	GO:0006297
5142	GO:0018406
5143	GO:0004849
5144	GO:0042396
5145	GO:0018103
5146	GO:0018317
5147	GO:0004775
5148	GO:0004806
5149	GO:0046040
5150	GO:0006241
5151	GO:0046218
5152	GO:0006228
5153	GO:0033765
5154	GO:0042158
5155	hydrophobicity_distribution_N.1.0_max
5156	GO:0006172
5157	GO:0042430
5158	GO:0004305
5159	GO:0050651
5160	GO:0046489
5161	GO:0004301
5162	GO:0008614
5163	GO:0008615

5164	GO:0004103
5165	GO:0034062
5166	GO:0047115
5167	GO:0006702
5168	GO:0004457
5169	GO:0050655
5170	GO:0032728
5171	GO:0033014
5172	GO:0042623
5173	GO:0006013
5174	in_local_density_0.1_max
5175	GO:0004060
5176	GO:0004090
5177	GO:0000030
5178	GO:0004065
5179	in_local_density_0_max
5180	GO:0046146
5181	GO:0004017
5182	GO:0032993
5183	GO:0006681
5184	GO:0035268
5185	GO:0004582
5186	GO:0035269
5187	GO:0031501
5188	GO:0003887
5189	GO:0006166
5190	GO:0003896
5191	GO:0034061
5192	GO:0003899
5193	GO:0006104
5194	GO:0004550
5195	GO:0004461
5196	in_local_density_0.2_max
5197	GO:0004459
5198	GO:0003945
5199	GO:0017110
5200	GO:0004558
5201	GO:0006703
5202	GO:0005775
5203	GO:0009208
5204	GO:0009150
5205	GO:0006772
5206	GO:0009148
5207	GO:0009147
5208	GO:0009180
5209	secondary_structure_distribution_N.1.0_max
5210	GO:0042816

5211	GO:0006569
5212	GO:0006493
5213	GO:0034416
5214	GO:0009135
5215	GO:0009141
5216	GO:0006360
5217	GO:0006505
5218	GO:0009136
5219	GO:0042723
5220	GO:0046031
5221	GO:0009152
5222	GO:0016779
5223	GO:0006497
5224	GO:0000050
5225	GO:0006586
5226	GO:0043174
5227	GO:0043101
5228	GO:0000428
5229	GO:0008484
5230	GO:0043604
5231	GO:0008757
5232	GO:0055029
5233	GO:0009435
5234	GO:0008401
5235	GO:0009179
5236	GO:0006506
5237	GO:0008376
5238	GO:0042819
5239	GO:0030880
5240	GO:0030206
5241	GO:0016165
5242	GO:0043202
5243	GO:0006354
5244	GO:0042436
5245	GO:0006368
5246	GO:0006779
5247	GO:0032549
5248	GO:0005736
5249	GO:0046874
5250	GO:0006783
5251	GO:0046036
5252	GO:0009209
5253	GO:0003723
5254	GO:0006383
5255	GO:0042434
5256	GO:0042559
5257	GO:0005665

5258	GO:0006568
5259	GO:0016126
5260	GO:0005666
5261	GO:0009188
5262	GO:0015014
5263	GO:0006800
5264	GO:0032781
5265	GO:0042438
5266	GO:0009214
5267	GO:0006659
5268	GO:0004115
5269	GO:0016595
5270	GO:0004768
5271	GO:0042428
5272	GO:0004312
5273	GO:0033162
5274	GO:0045009
5275	GO:0006704
5276	GO:0006658
5277	GO:0016255
5278	GO:0070328
5279	GO:0008184
5280	GO:0050664
5281	GO:0004645
5282	GO:0003997
5283	GO:0045717
5284	GO:0002063
5285	GO:0090101
5286	GO:0043619
5287	GO:0009111
5288	GO:0022891
5289	GO:0042363
5290	GO:0043618
5291	GO:0008324
5292	GO:0005788
5293	GO:0031526
5294	GO:0004630
5295	GO:0030867
5296	GO:0042982
5297	GO:0009750
5298	GO:0009081
5299	GO:0042594
5300	GO:0051597
5301	GO:0033692
5302	GO:0043226
5303	GO:0043229
5304	GO:0006306

5305	GO:0006305
5306	GO:0006304
5307	GO:0032364
5308	GO:0007271
5309	GO:0055081
5310	GO:0008206
5311	GO:0043237
5312	GO:0006397
5313	polarity_distribution_H.1.0_max
5314	GO:0016706
5315	GO:0016787
5316	GO:0042627
5317	GO:0034361
5318	GO:0034382
5319	GO:0006801
5320	GO:0034385
5321	GO:0034384
5322	GO:0042554
5323	GO:0006885
5324	GO:0046716
5325	GO:0034377
5326	GO:0005980
5327	GO:0006707
5328	GO:0065005
5329	GO:0016127
5330	GO:0033238
5331	GO:0001972
5332	GO:0006434
5333	GO:0031543
5334	GO:0006591
5335	GO:0042415
5336	GO:0005882
5337	GO:0045111
5338	GO:0004828
5339	GO:0035094
5340	GO:0032994
5341	GO:0034358
5342	GO:0034372
5343	GO:0034375
5344	GO:0034370
5345	GO:0034364
5346	GO:0030002
5347	GO:0030312
5348	GO:0045932
5349	GO:0009058
5350	GO:0006699
5351	GO:0034435

5352	GO:0006525
5353	GO:0034433
5354	GO:0034434
5355	GO:0046034
5356	GO:0046219
5357	GO:0051567
5358	GO:0051568
5359	GO:0045261
5360	GO:0048385
5361	GO:0044455
5362	GO:0034220
5363	GO:0045259
5364	GO:0045240
5365	GO:0045263
5366	GO:0033180
5367	GO:0033176
5368	GO:0042435
5369	GO:0033178
5370	GO:0042168
5371	GO:0033177
5372	GO:0033015
5373	GO:0045426
5374	GO:0034968
5375	GO:0033179
5376	GO:0034653
5377	GO:0042776
5378	GO:0042765
5379	GO:0048387
5380	GO:0033013
5381	GO:0042054
5382	GO:0000033
5383	GO:0006560
5384	GO:0009215
5385	GO:0006562
5386	GO:0006561
5387	GO:0009205
5388	GO:0070035
5389	GO:0006552
5390	GO:0016279
5391	GO:0009206
5392	GO:0016278
5393	GO:0006564
5394	GO:0009151
5395	GO:0006596
5396	GO:0016744
5397	GO:0016724
5398	GO:0070815

5399	GO:0009201
5400	GO:0006595
5401	GO:0009199
5402	GO:0070568
5403	GO:0009144
5404	GO:0009226
5405	GO:0006491
5406	GO:0006488
5407	GO:0006526
5408	GO:0000309
5409	GO:0008900
5410	GO:0015077
5411	GO:0006487
5412	GO:0008276
5413	GO:0008553
5414	GO:0009142
5415	GO:0015018
5416	GO:0015405
5417	GO:0015399
5418	GO:0015924
5419	GO:0015923
5420	GO:0000015
5421	GO:0000276
5422	GO:0015078
5423	GO:0000275
5424	GO:0009145
5425	GO:0008170
5426	GO:0022890
5427	GO:0022857
5428	GO:0006743
5429	GO:0022892
5430	GO:0043492
5431	GO:0031545
5432	GO:0022804
5433	GO:0008216
5434	GO:0006744
5435	GO:0019829
5436	GO:0006754
5437	GO:0042625
5438	GO:0042626
5439	GO:0034481
5440	GO:0006818
5441	GO:0046933
5442	GO:0006778
5443	GO:0042799
5444	GO:0006787
5445	GO:0008295

5446	GO:0008475
5447	GO:0019798
5448	GO:0050510
5449	GO:0016820
5450	GO:0009103
5451	GO:0050508
5452	GO:0017060
5453	GO:0017176
5454	GO:0016813
5455	GO:0016802
5456	GO:0016997
5457	GO:0016774
5458	GO:0006684
5459	GO:0006685
5460	GO:0018024
5461	GO:0008653
5462	GO:0047238
5463	GO:0046961
5464	GO:0047760
5465	GO:0047555
5466	GO:0047756
5467	GO:0008186
5468	GO:0000104
5469	GO:0004415
5470	GO:0016469
5471	GO:0004449
5472	GO:0004448
5473	GO:0004499
5474	GO:0004515
5475	GO:0016471
5476	GO:0004386
5477	GO:0006102
5478	GO:0004308
5479	GO:0006119
5480	GO:0004594
5481	GO:0004619
5482	GO:0009331
5483	GO:0004114
5484	GO:0006432
5485	GO:0004579
5486	GO:0004576
5487	GO:0004322
5488	GO:0004653
5489	GO:0003873
5490	GO:0003847
5491	GO:0003923
5492	GO:0003876

5493	GO:0003993
5494	GO:0004004
5495	GO:0003829
5496	GO:0003724
5497	GO:0006003
5498	GO:0004571
5499	GO:0004082
5500	GO:0004062
5501	GO:0004111
5502	GO:0004083
5503	GO:0004112
5504	GO:0004013
5505	GO:0004046
5506	GO:0004035
5507	GO:0004634
5508	GO:0008146
5509	GO:0008250
5510	GO:0015985
5511	GO:0005753
5512	GO:0018242
5513	GO:0018196
5514	GO:0015937
5515	GO:0015988
5516	GO:0015986
5517	GO:0015992
5518	GO:0004656
5519	GO:0016571
5520	GO:0015991
5521	GO:0018243
5522	GO:0016254
5523	GO:0004802
5524	GO:0008109
5525	GO:0005947
5526	GO:0004735
5527	GO:0004767
5528	GO:0005215
5529	GO:0015672
5530	GO:0004826
5531	GO:0008026
5532	GO:0018279
5533	GO:0008037
5534	GO:0000271
5535	GO:0042446
5536	GO:0055067
5537	GO:0050954
5538	GO:0042311
5539	GO:0044462

5540	GO:0030313
5541	GO:0001540
5542	GO:0042417
5543	GO:0044249
5544	GO:0007186
5545	GO:0012510
5546	GO:0004568
5547	GO:0004815
5548	secondary_structure_distribution_P.1.0_max
5549	GO:0006422
5550	GO:0007606
5551	GO:0003008
5552	GO:0050877
5553	GO:0050890
5554	GO:0035381
5555	GO:0003886
5556	GO:0006030
5557	GO:0004984
5558	GO:0009008
5559	GO:0006032
5560	GO:0046920
5561	GO:0004064
5562	GO:0004063
5563	GO:0007600
5564	GO:0004931
5565	GO:0007608
5566	polarizability_distribution_N.1.0_max
5567	GO:0030130
5568	GO:0004930
5569	GO:0004888

(2) mRMR features list

Order	Feature name
1	secondary_structure_composition_P_max
2	solvent_accessibility_composition_H_mean
3	solvent_accessibility_distribution_H.0.75_max
4	GO:0043627
5	GO:0045121
6	secondary_structure_distribution_H.0.25_max
7	AA_composition_S_mean
8	secondary_structure_distribution_N.0.25_max
9	VanDerWaal_composition_P_max
10	GO:0043330
11	VanDerWaal_distribution_H.0.75_max
12	AA_composition_T_max

13	AA_composition_D_max
14	secondary_structure_distribution_H.0.5_max
15	GO:0048519
16	GO:0002687
17	secondary_structure_composition_P_mean
18	polarity_composition_N_max
19	GO:0042088
20	polarity_transition_NH_max
21	AA_composition_S_max
22	GO:0042063
23	polarizability_distribution_P.0.75_max
24	GO:0090068
25	GO:0014829
26	secondary_structure_distribution_H.0.75_max
27	AA_composition_Q_mean
28	GO:0030225
29	GO:0046661
30	hydrophobicity_composition_N_max
31	solvent_accessibility_distribution_H.0.0_max
32	polarity_distribution_P.0.5_max
33	polarizability_distribution_H.0.75_max
34	GO:0031594
35	GO:0031330
36	AA_composition_P_max
37	GO:0042953
38	GO:0048523
39	GO:0030217
40	GO:0007517
41	GO:0009913
42	GO:0042177
43	GO:0048641
44	hydrophobicity_distribution_N.0.75_max
45	hydrophobicity_distribution_H.0.75_max
46	GO:0022408
47	GO:0048608
48	GO:0045638
49	GO:0006897
50	GO:0030857
51	hydrophobicity_composition_P_max
52	GO:0051668
53	GO:0019079
54	AA_composition_V_mean
55	VanDerWaal_distribution_H.0.5_max
56	GO:0043125
57	GO:0004690
58	GO:0044057
59	GO:0071216

60	GO:0045137
61	polarity_distribution_N.0.75_max
62	GO:0045580
63	hydrophobicity_transition_PN_max
64	GO:0009411
65	GO:0045921
66	GO:0051492
67	secondary_structure_transition_PN_max
68	GO:0043393
69	polarizability_transition_PH_mean
70	GO:0010324
71	AA_composition_Q_max
72	secondary_structure_distribution_N.0.5_max
73	GO:0050995
74	polarity_distribution_N.0.0_max
75	GO:0043547
76	GO:0007098
77	GO:0019233
78	GO:0048747
79	GO:0048545
80	AA_composition_N_max
81	GO:0010001
82	GO:0045765
83	GO:0048169
84	GO:0051963
85	polarizability_distribution_H.0.5_max
86	GO:0014706
87	GO:0007548
88	GO:0045767
89	GO:0032320
90	secondary_structure_distribution_N.0.0_max
91	GO:0030855
92	secondary_structure_distribution_P.0.75_max
93	GO:0004722
94	GO:0045787
95	polarity_composition_H_max
96	GO:0005545
97	GO:0045670
98	GO:0010596
99	GO:0048167
100	GO:0043209
101	third_singular_values
102	GO:0045737
103	solvent_accessibility_composition_H_max
104	GO:0051297
105	GO:0030216
106	GO:0045727

107	GO:0032355
108	GO:0046982
109	GO:0045069
110	GO:0050930
111	GO:0003006
112	GO:0051057
113	GO:0032231
114	GO:0031575
115	GO:0040008
116	GO:0050807
117	GO:0046825
118	GO:0051129
119	GO:0042692
120	GO:0045930
121	solvent_accessibility_distribution_H.0.5_max
122	GO:0051353
123	GO:0007127
124	GO:0048259
125	polarity_distribution_P.0.75_max
126	GO:0045619
127	GO:0043204
128	GO:0002762
129	GO:0060537
130	GO:0043559
131	GO:0048708
132	polarizability_distribution_P.0.5_max
133	GO:0009612
134	hydrophobicity_transition_NH_mean
135	GO:0080010
136	GO:0050678
137	GO:0046579
138	GO:0045892
139	VanDerWaal_composition_N_max
140	GO:0048589
141	GO:0045822
142	GO:0045840
143	GO:0030511
144	AA_composition_M_max
145	GO:0010970
146	GO:0043407
147	GO:0050673
148	GO:0000226
149	GO:0016601
150	GO:0051253
151	secondary_structure_composition_H_mean
152	GO:0009991
153	VanDerWaal_distribution_P.0.0_max

154	GO:0007611
155	GO:0031069
156	GO:0048741
157	GO:0070227
158	GO:0032350
159	GO:0004691
160	GO:0005515
161	GO:0051785
162	GO:0007569
163	GO:0043149
164	polarizability_distribution_P.0.25_max
165	GO:0048732
166	GO:0043524
167	GO:0090183
168	polarity_distribution_N.0.5_max
169	GO:0005901
170	GO:0014911
171	GO:0032228
172	GO:0048869
173	VanDerWaal_distribution_P.0.75_max
174	GO:0002286
175	GO:0032026
176	GO:0040007
177	GO:0030168
178	GO:0051385
179	GO:0031646
180	AA_composition_T_mean
181	GO:0044459
182	GO:0035014
183	secondary_structure_distribution_P.0.25_max
184	GO:0048168
185	GO:0090184
186	GO:0005200
187	GO:0042551
188	GO:0007398
189	GO:0051271
190	GO:0010165
191	GO:0008542
192	GO:0051971
193	GO:0030097
194	GO:0009617
195	GO:0048730
196	GO:0032000
197	GO:0022602
198	GO:0008047
199	GO:0000122
200	GO:0007632

201	GO:0005856
202	GO:0045621
203	GO:0042221
204	secondary_structure_transition_PH_mean
205	GO:0016043
206	GO:0017148
207	GO:0007005
208	GO:0034109
209	GO:0044253
210	GO:0031328
211	VanDerWaal_distribution_P.0.5_max
212	GO:0050848
213	GO:0002763
214	GO:0001954
215	GO:0051932
216	hydrophobicity_distribution_H.0.5_max
217	GO:0042698
218	GO:0055002
219	GO:0003690
220	GO:0044089
221	GO:0043560
222	GO:0050792
223	GO:0047485
224	secondary_structure_distribution_H.0.0_max
225	GO:0051716
226	GO:0048592
227	GO:0035020
228	GO:0010212
229	AA_composition_C_max
230	GO:0050850
231	GO:0032770
232	graph_size
233	GO:0003704
234	polarity_composition_P_mean
235	GO:0051962
236	GO:0080134
237	GO:0051147
238	solvent_accessibility_transition_HE_max
239	GO:0035022
240	GO:0055001
241	GO:0048009
242	GO:0004715
243	GO:0034620
244	hydrophobicity_distribution_N.0.25_max
245	GO:0006357
246	GO:0045806
247	GO:0001933

248	GO:0031998
249	GO:0051965
250	hydrophobicity_distribution_H.0.25_max
251	GO:0048634
252	GO:0044419
253	GO:0043242
254	GO:0050926
255	GO:0030968
256	GO:0030154
257	GO:0046633
258	GO:0051272
259	GO:0051153
260	GO:0032369
261	VanDerWaal_distribution_P.0.5_mean
262	GO:0051168
263	GO:0045582
264	GO:0023034
265	GO:0008152
266	GO:0006915
267	GO:0048813
268	GO:0010033
269	polarity_transition_PN_mean
270	GO:0050927
271	GO:0071445
272	GO:0014015
273	GO:0043244
274	GO:0002039
275	GO:0060263
276	GO:0045216
277	GO:0050994
278	GO:0022898
279	secondary_structure_composition_N_max
280	GO:0009890
281	GO:0043029
282	GO:0016605
283	GO:0045600
284	GO:0043235
285	GO:0045935
286	GO:0009895
287	GO:0008022
288	GO:0003705
289	GO:0016202
290	secondary_structure_distribution_H.1.0_mean
291	GO:0051049
292	GO:0050853
293	GO:0008406
294	GO:0043198

295	GO:0051650
296	GO:0034762
297	GO:0048742
298	GO:0051341
299	GO:0009581
300	GO:0043583
301	GO:0008287
302	GO:0035116
303	GO:0040017
304	GO:0045596
305	GO:0007009
306	GO:0001541
307	GO:0051648
308	secondary_structure_distribution_P.0.5_max
309	GO:0035326
310	GO:0051128
311	GO:0043550
312	GO:0055123
313	GO:0002685
314	GO:0032891
315	GO:0030032
316	GO:0048103
317	AA_composition_P_mean
318	GO:0003056
319	GO:0051783
320	GO:0021700
321	GO:0051051
322	GO:0019898
323	GO:0022612
324	GO:0044087
325	GO:0048546
326	GO:0032890
327	GO:0001772
328	GO:0007006
329	GO:0051897
330	GO:0046640
331	GO:0022607
332	GO:0004723
333	GO:0007088
334	GO:0022415
335	GO:0032839
336	GO:0010648
337	GO:0050681
338	GO:0050820
339	GO:0014009
340	GO:0014068
341	GO:0051240

342	hydrophobicity_distribution_P.0.0_max
343	GO:0032434
344	GO:0031435
345	GO:0050769
346	GO:0051146
347	GO:0032768
348	GO:0001667
349	GO:0006906
350	GO:0016032
351	GO:0061061
352	GO:0048639
353	GO:0034976
354	GO:0005829
355	VanDerWaal_transition_NH_max
356	GO:0005905
357	GO:0050777
358	GO:0032838
359	GO:0051496
360	GO:0040013
361	GO:0032403
362	GO:0051173
363	GO:0030098
364	out_out_topological_max
365	GO:0004697
366	hydrophobicity_composition_H_mean
367	GO:0031647
368	GO:0030307
369	GO:0051412
370	GO:0051254
371	GO:0007568
372	GO:0002690
373	GO:0050806
374	GO:0032233
375	GO:0030163
376	GO:0046777
377	GO:0005575
378	GO:0046326
379	GO:0001822
380	GO:0045066
381	polarity_transition_PH_max
382	GO:0010714
383	secondary_structure_distribution_H.0.0_mean
384	GO:0007519
385	GO:0019900
386	GO:0032412
387	GO:0060350
388	GO:0051000

389	GO:0044085
390	GO:0048839
391	GO:0004672
392	GO:0030336
393	GO:0010828
394	GO:0072001
395	GO:0002688
396	GO:0019058
397	GO:0070887
398	polarizability_distribution_N.0.0_max
399	GO:0051093
400	GO:0032967
401	GO:0034765
402	GO:0008219
403	GO:0060538
404	GO:0019902
405	GO:0045725
406	GO:0005488
407	GO:0050869
408	GO:0046578
409	GO:0009653
410	GO:0010631
411	VanDerWaal_transition_PH_mean
412	GO:0019899
413	GO:0000003
414	GO:0030234
415	GO:0045793
416	GO:0032496
417	GO:0070507
418	GO:0070875
419	GO:0044246
420	GO:0007635
421	GO:0019903
422	GO:0016265
423	GO:0048041
424	GO:0006611
425	GO:0051056
426	GO:0010634
427	GO:0045736
428	GO:0050870
429	GO:0023052
430	GO:0035137
431	GO:0045429
432	GO:0045687
433	GO:0007612
434	GO:0090132
435	GO:0008150

436	GO:0045927
437	GO:0042325
438	GO:0032318
439	GO:0050808
440	GO:0019218
441	GO:0007093
442	AA_composition_I_max
443	GO:0032879
444	GO:0006983
445	GO:0045741
446	GO:0019059
447	GO:0010557
448	GO:0043241
449	GO:0030018
450	GO:0070423
451	GO:0090130
452	GO:0016481
453	GO:0044265
454	GO:0043069
455	GO:0046546
456	GO:0043548
457	GO:0016773
458	GO:0030162
459	GO:0010310
460	out_in_topological_max
461	GO:0048856
462	GO:0019220
463	GO:0032984
464	GO:0005952
465	GO:0010632
466	GO:0031023
467	GO:0007154
468	GO:0070431
469	GO:0016044
470	GO:0001968
471	GO:0009725
472	GO:0031347
473	GO:0000079
474	GO:0045944
475	GO:0012501
476	GO:0019904
477	GO:0060548
478	GO:0051495
479	GO:0071695
480	GO:0006468
481	GO:0010629
482	GO:0051174

483	GO:0048469
484	GO:0061024
485	AA_composition_G_max
486	GO:0004674
487	GO:0051015
488	GO:0045893
489	GO:0015911
490	GO:0033077
491	GO:0021782
492	GO:0003702
493	GO:0002237
494	GO:0032502
495	GO:0050866
496	GO:0033059
497	GO:0009582
498	GO:0048660
499	GO:0045685
500	GO:0021953
501	GO:0010907
502	GO:0031226
503	GO:0010628
504	GO:0043687
505	GO:0002366
506	AA_composition_D_mean
507	GO:0009314
508	GO:0048731
509	GO:0045940
510	GO:0002761
511	VanDerWaal_distribution_N.0.0_max
512	GO:0055114
513	in_local_density_0.9_max
514	GO:0048659
515	GO:0007242
516	GO:0045941
517	GO:0045646
518	GO:0034329
519	GO:0001816
520	GO:0001558
521	GO:0007416
522	GO:0042641
523	GO:0051494
524	GO:0043270
525	GO:0048538
526	GO:0002263
527	GO:0046620
528	GO:0070141
529	GO:0009891

530	GO:0006366
531	GO:0002028
532	GO:0000186
533	GO:0048583
534	GO:0009888
535	GO:0010941
536	GO:0004683
537	GO:0047496
538	GO:0016564
539	GO:0090066
540	GO:0046631
541	GO:0035051
542	GO:0002285
543	GO:0002360
544	hydrophobicity_distribution_P.0.25_max
545	GO:0046827
546	GO:0043067
547	GO:0048705
548	polarizability_composition_N_mean
549	GO:0045428
550	GO:0007566
551	GO:0032409
552	GO:0051017
553	GO:0033028
554	GO:0031434
555	GO:0044257
556	GO:0048646
557	GO:0045597
558	GO:0019901
559	GO:0030048
560	GO:0048661
561	GO:0010389
562	GO:0010638
563	secondary_structure_distribution_N.0.75_max
564	GO:0043537
565	GO:0030546
566	GO:0032663
567	GO:0051603
568	GO:0090150
569	GO:0032582
570	AA_composition_F_max
571	GO:0006469
572	GO:0030275
573	GO:0046885
574	GO:0044424
575	GO:0010332
576	GO:0030194

577	GO:0048143
578	GO:0046824
579	GO:0060606
580	GO:0007270
581	GO:0045768
582	GO:0090004
583	GO:0017016
584	GO:0006984
585	GO:0006903
586	GO:0014070
587	GO:0002262
588	GO:0044092
589	GO:0051270
590	GO:0019207
591	GO:0021930
592	hydrophobicity_distribution_P.0.5_max
593	GO:0090002
594	GO:0043535
595	GO:0010604
596	secondary_structure_distribution_P.0.0_max
597	GO:0032368
598	GO:0030125
599	GO:0032800
600	GO:0001843
601	GO:0009628
602	GO:0060429
603	GO:0043087
604	GO:0090003
605	GO:0014002
606	GO:0031349
607	AA_composition_A_mean
608	GO:0042391
609	GO:0021534
610	GO:0019722
611	GO:0048513
612	GO:0051348
613	GO:0051969
614	GO:0045749
615	GO:0042633
616	GO:0001948
617	GO:0006996
618	GO:0050679
619	GO:0050863
620	GO:0023051
621	GO:0045668
622	GO:0007266
623	GO:0010875

624	GO:0030120
625	GO:0031327
626	GO:0050901
627	GO:0008361
628	GO:0005070
629	AA_composition_W_mean
630	GO:0040014
631	GO:0007051
632	GO:0043523
633	GO:0007213
634	polarity_distribution_P.0.25_max
635	GO:0021924
636	GO:0001942
637	GO:0007507
638	first_singular_values
639	GO:0052547
640	GO:0009966
641	GO:0030118
642	GO:0010874
643	GO:0007050
644	GO:0043005
645	GO:0032612
646	GO:0006796
647	GO:0050764
648	GO:0031659
649	GO:0034613
650	GO:0022409
651	GO:0051250
652	GO:0045444
653	GO:0022405
654	hydrophobicity_transition_PH_max
655	GO:0030132
656	GO:0043900
657	GO:0016049
658	GO:0044430
659	GO:0007281
660	GO:0042169
661	GO:0043281
662	GO:0031657
663	GO:0045649
664	GO:0032092
665	GO:0006793
666	hydrophobicity_distribution_N.0.0_max
667	GO:0070727
668	GO:0017022
669	GO:0007492
670	GO:0046903

671	GO:0045922
672	GO:0032535
673	GO:0005769
674	GO:0022404
675	GO:0002695
676	GO:0016491
677	GO:0005938
678	GO:0050821
679	GO:0010559
680	GO:0032652
681	GO:0048524
682	GO:0052548
683	GO:0042303
684	GO:0033673
685	GO:0001889
686	GO:0045834
687	GO:0014065
688	GO:0021954
689	GO:0009416
690	AA_composition_F_mean
691	GO:0042981
692	GO:0032940
693	GO:0046321
694	GO:0048511
695	GO:0010745
696	GO:0009986
697	GO:0030117
698	GO:0032611
699	GO:0061008
700	GO:0007528
701	GO:0042108
702	GO:0043566
703	GO:0030175
704	GO:0032886
705	GO:0032615
706	GO:0048475
707	polarity_distribution_H.0.75_max
708	GO:0050999
709	GO:0032651
710	solvent_accessibility_distribution_H.0.25_max
711	GO:0043232
712	GO:0009790
713	GO:0051291
714	GO:0043025
715	GO:0050732
716	GO:0048745
717	GO:0003779

718	GO:0051219
719	GO:0060541
720	GO:0032655
721	GO:0043228
722	GO:0051726
723	GO:0045730
724	GO:0048339
725	GO:0005100
726	GO:0010692
727	polarity_distribution_N.0.25_mean
728	GO:0030889
729	GO:0046329
730	GO:0043278
731	GO:0044297
732	GO:0000910
733	GO:0051318
734	GO:0042176
735	GO:0051930
736	VanDerWaal_distribution_N.0.75_max
737	GO:0001763
738	GO:0051020
739	GO:0070303
740	GO:0002532
741	GO:0000080
742	GO:0032964
743	GO:0044456
744	GO:0014066
745	GO:0048468
746	GO:0014072
747	GO:0043409
748	VanDerWaal_distribution_N.0.75_mean
749	GO:0051931
750	GO:0070201
751	GO:0007264
752	GO:0007155
753	GO:0005819
754	VanDerWaal_transition_PN_max
755	AA_composition_E_mean
756	hydrophobicity_composition_H_max
757	GO:0031325
758	GO:0008624
759	GO:0010765
760	GO:0050804
761	GO:0030674
762	GO:0035265
763	GO:0022610
764	GO:0032880

765	GO:0030858
766	GO:0005942
767	GO:0031988
768	GO:0046902
769	GO:0042516
770	GO:0005912
771	GO:0005667
772	GO:0043551
773	GO:0010748
774	AA_composition_K_max
775	GO:0048286
776	GO:0007613
777	GO:0031333
778	GO:0070161
779	GO:0010608
780	GO:0051172
781	GO:0046666
782	GO:0010746
783	GO:0014020
784	GO:0018105
785	GO:0000077
786	GO:0090218
787	GO:0007616
788	GO:0030856
789	GO:0043542
790	GO:0006836
791	GO:0010149
792	GO:0030100
793	GO:0016303
794	GO:0010558
795	GO:0002520
796	GO:0010811
797	out_local_density_0.9_max
798	GO:0048522
799	GO:0030031
800	GO:0010639
801	GO:0000307
802	out_locta_density_0.2_mean
803	GO:0045934
804	GO:0046834
805	GO:0031399
806	GO:0051493
807	GO:0048534
808	GO:0033043
809	GO:0006913
810	GO:0010171
811	GO:0008603

812	GO:0043552
813	GO:0001727
814	GO:0030705
815	GO:0002699
816	GO:0032844
817	GO:0003697
818	GO:0051050
819	hydrophobicity_transition_NH_max
820	polarity_distribution_N.0.25_max
821	GO:0045637
822	GO:0005887
823	GO:0043085
824	GO:0046934
825	GO:0051169
826	GO:0031644
827	GO:0008643
828	GO:0045786
829	GO:0008092
830	GO:0007269
831	GO:0009719
832	GO:0045807
833	GO:0045939
834	GO:0030218
835	GO:0019897
836	GO:0009267
837	GO:0008544
838	GO:0035004
839	GO:0007399
840	GO:0044106
841	polarizability_transition_PH_max
842	GO:0032352
843	GO:0044445
844	GO:0051336
845	GO:0010894
846	GO:0008637
847	GO:0048754
848	GO:0046886
849	GO:0045913
850	GO:0031701
851	GO:0030035
852	polarizability_composition_P_max
853	GO:0010605
854	GO:0046854
855	GO:0050795
856	polarity_distribution_N.0.0_mean
857	GO:0051094
858	GO:0031032

859	GO:0032623
860	GO:0016322
861	GO:0002347
862	GO:0010676
863	GO:0016614
864	GO:0048146
865	GO:0030239
866	GO:0044281
867	GO:0042094
868	GO:0009887
869	GO:0032436
870	GO:0031267
871	GO:0010712
872	GO:0060627
873	GO:0016538
874	GO:0051402
875	GO:0045214
876	GO:0016903
877	GO:0008585
878	GO:0008584
879	GO:0051145
880	GO:0032965
881	GO:0030235
882	GO:0070997
883	GO:0044093
884	GO:0010721
885	GO:0005955
886	GO:0042090
887	GO:0042326
888	GO:0034330
889	GO:0043066
890	GO:0045671
891	GO:0008629
892	GO:0050768
893	GO:0010888
894	GO:0050996
895	GO:0043062
896	GO:0007406
897	GO:0007265
898	GO:0022414
899	hydrophobicity_distribution_N.0.5_max
900	GO:0006814
901	GO:0050776
902	GO:0031667
903	GO:0030666
904	GO:0048547
905	out_locta_density_0_mean

906	GO:0006605
907	GO:0050803
908	GO:0060255
909	GO:0042102
910	GO:0001569
911	GO:0032722
912	GO:0045084
913	GO:0015629
914	GO:0042640
915	GO:0002437
916	GO:0050918
917	GO:0007423
918	GO:0032088
919	GO:0043269
920	GO:0048340
921	GO:0006417
922	GO:0042130
923	GO:0048520
924	GO:0031143
925	GO:0045075
926	GO:0009792
927	GO:0032695
928	GO:0045595
929	solvent_accessibility_distribution_H.0.0_mean
930	GO:0000082
931	GO:0046325
932	polarity_distribution_P.0.25_mean
933	GO:0051960
934	GO:0048284
935	GO:0005916
936	GO:0048870
937	GO:0032504
938	GO:0051384
939	GO:0043009
940	out_locta_density_0.1_mean
941	GO:0002274
942	GO:0002009
943	GO:0033267
944	GO:0048518
945	GO:0030863
946	GO:0051241
947	GO:0009925
948	polarity_distribution_H.0.0_max
949	GO:0016477
950	GO:0048609
951	GO:0014704
952	GO:0032559

953	GO:0031324
954	GO:0043406
955	GO:0031623
956	GO:0006916
957	GO:0048483
958	GO:0051674
959	GO:0046641
960	GO:0001655
961	solvent_accessibility_distribution_H.0.5_mean
962	GO:0043500
963	GO:0045178
964	GO:0016566
965	GO:0045862
966	GO:0044291
967	GO:0046324
968	GO:0007267
969	GO:0001952
970	GO:0045639
971	GO:0009894
972	GO:0032583
973	GO:0043394
974	GO:0031099
975	polarity_composition_P_max
976	GO:0001782
977	GO:0004693
978	GO:0040012
979	GO:0008286
980	GO:0032432
981	GO:0009893
982	GO:0006928
983	GO:0015758
984	GO:0009892
985	GO:0004713
986	GO:0032410
987	GO:0006298
988	GO:0050727
989	GO:0043193
990	GO:0007173
991	GO:0044449
992	GO:0031960
993	GO:0009897
994	GO:0046545
995	GO:0002248
996	GO:0006970
997	GO:0001725
998	GO:0015749
999	hydrophobicity_distribution_P.0.75_max

1000	hydrophobicity_distribution_H.0.25_mean
1001	GO:0050920
1002	GO:0030676
1003	GO:0051345
1004	GO:0046660
1005	GO:0048144
1006	GO:0008645
1007	GO:0030016
1008	polarity_distribution_H.0.25_max
1009	GO:0010893
1010	GO:0032570
1011	GO:0050921
1012	GO:0001817
1013	GO:0012505
1014	GO:0048145
1015	GO:0007565
1016	GO:0042098
1017	GO:0051968
1018	GO:0014074
1019	GO:0001944
1020	GO:0017076
1021	GO:0014013
1022	GO:0022407
1023	GO:0070231
1024	GO:0030054
1025	GO:0050868
1026	GO:0034641
1027	GO:0001656
1028	GO:0048584
1029	GO:0030554
1030	GO:0043297
1031	GO:0032868
1032	GO:0033002
1033	GO:0006917
1034	GO:0001568
1035	GO:0030224
1036	GO:0050709
1037	GO:0048645
1038	GO:0046634
1039	GO:0016342
1040	GO:0033032
1041	secondary_structure_distribution_H.0.25_mean
1042	GO:0010921
1043	GO:0001759
1044	GO:0012502
1045	GO:0043405
1046	GO:0043624

1047	GO:0005622
1048	GO:0032386
1049	GO:0016247
1050	GO:0031346
1051	GO:0006809
1052	GO:0034623
1053	GO:0045995
1054	GO:0002521
1055	GO:0042592
1056	GO:0050798
1057	GO:0016616
1058	GO:0045861
1059	GO:0046822
1060	GO:0010829
1061	GO:0017166
1062	GO:0051641
1063	GO:0014902
1064	GO:0060326
1065	GO:0002711
1066	GO:0002244
1067	GO:0046323
1068	GO:0016620
1069	GO:0030551
1070	polarizability_composition_H_mean
1071	GO:0000018
1072	GO:0046209
1073	GO:0030029
1074	GO:0030425
1075	GO:0033157
1076	GO:0004428
1077	GO:0042035
1078	GO:0048246
1079	GO:0000902
1080	GO:0010552
1081	GO:0051279
1082	GO:0006081
1083	GO:0008285
1084	GO:0045445
1085	GO:0016909
1086	GO:0008283
1087	GO:0007275
1088	GO:0060589
1089	GO:0051059
1090	GO:0060284
1091	GO:0010959
1092	GO:0071706
1093	GO:0060556

1094	GO:0004028
1095	GO:0008134
1096	GO:0030335
1097	GO:0005884
1098	GO:0005623
1099	GO:0051893
1100	GO:0042368
1101	GO:0045063
1102	GO:0051403
1103	VanDerWaal_composition_H_mean
1104	GO:0030877
1105	GO:0030695
1106	GO:0033554
1107	GO:0048585
1108	GO:0030324
1109	GO:0046632
1110	GO:0090109
1111	GO:0009408
1112	GO:0044464
1113	GO:0030296
1114	GO:0032330
1115	GO:0008104
1116	GO:0042476
1117	GO:0060558
1118	GO:0002819
1119	polarizability_distribution_P.0.0_max
1120	GO:0010039
1121	GO:0030323
1122	GO:0043502
1123	GO:0009968
1124	GO:0061035
1125	GO:0030315
1126	GO:0002822
1127	GO:0006446
1128	GO:0051222
1129	GO:0042127
1130	GO:0051100
1131	GO:0009950
1132	GO:0016301
1133	GO:0030656
1134	GO:0035313
1135	GO:0042995
1136	GO:0045076
1137	GO:0023057
1138	GO:0030165
1139	GO:0002709
1140	AA_composition_E_max

1141	GO:0010646
1142	GO:0022403
1143	GO:0048514
1144	GO:0034113
1145	GO:0045202
1146	GO:0016310
1147	solvent_accessibility_distribution_H.1.0_mean
1148	GO:0031674
1149	GO:0004861
1150	GO:0045086
1151	GO:0048878
1152	GO:0048820
1153	GO:0016651
1154	GO:0007420
1155	GO:0051261
1156	GO:0002682
1157	GO:0016023
1158	GO:0007162
1159	GO:0002702
1160	GO:0048638
1161	GO:0001701
1162	GO:0030316
1163	GO:0010922
1164	GO:0019717
1165	GO:0051130
1166	GO:0010543
1167	GO:0032101
1168	GO:0045620
1169	GO:0045579
1170	GO:0002821
1171	GO:0046677
1172	GO:0030155
1173	GO:0000302
1174	GO:0002294
1175	GO:0043112
1176	GO:0051325
1177	GO:0021675
1178	GO:0046626
1179	GO:0007610
1180	GO:0035257
1181	GO:0005154
1182	GO:0033365
1183	GO:0045294
1184	GO:0051329
1185	polarity_composition_H_mean
1186	GO:0035239
1187	GO:0002824

1188	GO:0032553
1189	GO:0002246
1190	GO:0032989
1191	GO:0043367
1192	GO:0043433
1193	GO:0032869
1194	GO:0017145
1195	GO:0007243
1196	GO:0051701
1197	GO:0031669
1198	GO:0002684
1199	GO:0032569
1200	GO:0002293
1201	GO:0005057
1202	GO:0032555
1203	GO:0090048
1204	GO:0034504
1205	GO:0046700
1206	GO:0048871
1207	hydrophobicity_transition_PN_mean
1208	GO:0022008
1209	GO:0051099
1210	GO:0016328
1211	GO:0035264
1212	GO:0002287
1213	GO:0000790
1214	GO:0021940
1215	GO:0048709
1216	GO:0021872
1217	GO:0010827
1218	GO:0002429
1219	GO:0048599
1220	GO:0048872
1221	GO:0051208
1222	GO:0010551
1223	GO:0010594
1224	GO:0070663
1225	GO:0021936
1226	GO:0060986
1227	GO:0002292
1228	GO:0043292
1229	GO:0033081
1230	GO:0045165
1231	GO:0051540
1232	GO:0009994
1233	GO:0032944
1234	GO:0045672

1235	GO:0031528
1236	GO:0030334
1237	GO:0050790
1238	GO:0043370
1239	GO:0016563
1240	GO:0050900
1241	GO:0051536
1242	GO:0030036
1243	GO:0031571
1244	GO:0021895
1245	GO:0001570
1246	GO:0050670
1247	GO:0060349
1248	GO:0009896
1249	GO:0045630
1250	GO:0016079
1251	GO:0010939
1252	GO:0006898
1253	GO:0007172
1254	GO:0048011
1255	GO:0051895
1256	GO:0030182
1257	GO:0030742
1258	GO:0042129
1259	GO:0006519
1260	GO:0043372
1261	GO:0045742
1262	GO:0032589
1263	AA_composition_C_mean
1264	GO:0016323
1265	GO:0043491
1266	GO:0002828
1267	GO:0015800
1268	GO:0030900
1269	GO:0005979
1270	GO:0001960
1271	GO:0010942
1272	GO:0005524
1273	GO:0042092
1274	GO:0001932
1275	GO:0051224
1276	GO:0006520
1277	GO:0022411
1278	GO:0019002
1279	GO:0035295
1280	GO:0046322
1281	GO:0009651

1282	GO:0042093
1283	GO:0043065
1284	GO:0070873
1285	GO:0015711
1286	GO:0001883
1287	GO:0002708
1288	GO:0065009
1289	GO:0010035
1290	GO:0002253
1291	GO:0007044
1292	GO:0045628
1293	GO:0005759
1294	GO:0010962
1295	GO:0006909
1296	GO:0060491
1297	GO:0015813
1298	GO:0043392
1299	VanDerWaal_distribution_H.0.25_max
1300	GO:0043616
1301	GO:0001882
1302	GO:0070661
1303	GO:0048489
1304	GO:0043068
1305	GO:0032885
1306	GO:0007043
1307	GO:0002275
1308	GO:0045064
1309	GO:0042475
1310	GO:0031980
1311	GO:0002705
1312	GO:0032881
1313	GO:0001654
1314	GO:0019226
1315	VanDerWaal_distribution_P.0.25_max
1316	GO:0032507
1317	GO:0051898
1318	GO:0030027
1319	GO:0050865
1320	GO:0000123
1321	GO:0003674
1322	GO:0045185
1323	GO:0006955
1324	GO:0046636
1325	GO:0002021
1326	GO:0060351
1327	GO:0071375
1328	GO:0032387

1329	GO:0080090
1330	polarizability_distribution_H.0.25_max
1331	GO:0004030
1332	GO:0050851
1333	GO:0048699
1334	GO:0050867
1335	GO:0003015
1336	GO:0050966
1337	GO:0007089
1338	GO:0051925
1339	GO:0048536
1340	GO:0004712
1341	GO:0006939
1342	GO:0046823
1343	GO:0046658
1344	GO:0007169
1345	GO:0002696
1346	GO:0031016
1347	GO:0030593
1348	GO:0070482
1349	GO:0004708
1350	GO:0006968
1351	GO:0032388
1352	GO:0060047
1353	GO:0032371
1354	GO:0002694
1355	GO:0008635
1356	GO:0051052
1357	GO:0016525
1358	GO:0019894
1359	GO:0045859
1360	GO:0005089
1361	GO:0046651
1362	GO:0045839
1363	GO:0032374
1364	GO:0032604
1365	GO:0006886
1366	polarizability_distribution_P.0.75_mean
1367	GO:0033044
1368	GO:0070851
1369	GO:0043353
1370	GO:0051784
1371	polarizability_transition_NH_mean
1372	GO:0030139
1373	polarity_distribution_H.1.0_mean
1374	GO:0032943
1375	GO:0048806

1376	GO:0033619
1377	GO:0071310
1378	GO:0044403
1379	GO:0060395
1380	GO:0045651
1381	GO:0042253
1382	GO:0031331
1383	GO:0042492
1384	GO:0051249
1385	GO:0060341
1386	GO:0050974
1387	GO:0043434
1388	GO:0046629
1389	GO:0050982
1390	GO:0004860
1391	GO:0051251
1392	GO:0040036
1393	GO:0001911
1394	GO:0051239
1395	in_in_topological_max
1396	GO:0019210
1397	GO:0002707
1398	GO:0031668
1399	GO:0007167
1400	GO:0044444
1401	GO:0031570
1402	GO:0001837
1403	GO:0010574
1404	GO:0001934
1405	GO:0045884
1406	GO:0030297
1407	GO:0031342
1408	GO:0032411
1409	polarizability_composition_N_max
1410	GO:0021766
1411	GO:0048548
1412	GO:0071496
1413	GO:0008360
1414	GO:0001841
1415	GO:0010573
1416	GO:0051246
1417	GO:0002704
1418	GO:0000187
1419	GO:0007417
1420	GO:0006907
1421	GO:0042522
1422	GO:0046006

1423	GO:0000075
1424	GO:0007268
1425	GO:0030879
1426	GO:0045833
1427	GO:0010575
1428	polarizability_distribution_N.0.25_max
1429	polarizability_transition_NH_max
1430	GO:0002228
1431	GO:0035249
1432	GO:0006413
1433	GO:0009952
1434	GO:0007204
1435	GO:0004707
1436	GO:0016831
1437	GO:0010564
1438	GO:0002218
1439	GO:0035023
1440	GO:0042267
1441	GO:0031648
1442	GO:0050912
1443	GO:0002260
1444	GO:0048596
1445	VanDerWaal_transition_PH_max
1446	topological_change_0.6_0.7
1447	GO:0045191
1448	GO:0001947
1449	GO:0002758
1450	GO:0048070
1451	GO:0010468
1452	GO:0001580
1453	GO:0045168
1454	GO:0045911
1455	GO:0048048
1456	GO:0031128
1457	GO:0031102
1458	polarizability_distribution_N.0.75_max
1459	GO:0045830
1460	GO:0051546
1461	GO:0030427
1462	GO:0001953
1463	GO:0042976
1464	GO:0030295
1465	GO:0051209
1466	GO:0031076
1467	GO:0009987
1468	GO:0030055
1469	GO:0060553

1470	GO:0051283
1471	GO:0043113
1472	GO:0021955
1473	GO:0022603
1474	GO:0042107
1475	GO:0051338
1476	GO:0005913
1477	GO:0008385
1478	GO:0035176
1479	GO:0005925
1480	GO:0051282
1481	GO:0043549
1482	GO:0042089
1483	hydrophobicity_distribution_P.1.0_mean
1484	GO:0005924
1485	GO:0010862
1486	GO:0019887
1487	GO:0046330
1488	GO:0080135
1489	GO:0007010
1490	GO:0005902
1491	GO:0070266
1492	GO:0034097
1493	GO:0019209
1494	GO:0021697
1495	GO:0035258
1496	GO:0045785
1497	GO:0051188
1498	GO:0001776
1499	GO:0031406
1500	GO:0051896
1501	GO:0060555
1502	GO:0034101
1503	GO:0001726
1504	GO:0031334
1505	GO:0007171
1506	GO:0008194
1507	GO:0070688
1508	GO:0060545
1509	GO:0001937
1510	GO:0008593
1511	GO:0010927
1512	GO:0048567
1513	GO:0045634
1514	GO:0051186
1515	GO:0060544
1516	GO:0043234

1517	GO:0005576
1518	GO:0021781
1519	GO:0050932
1520	GO:0007439
1521	GO:0010038
1522	GO:0005021
1523	GO:0043632
1524	GO:0051607
1525	GO:0004033
1526	GO:0043086
1527	GO:0009308
1528	GO:0014037
1529	GO:0032469
1530	GO:0042509
1531	GO:0031252
1532	GO:0042060
1533	GO:0019941
1534	secondary_structure_transition_PH_max
1535	GO:0051427
1536	GO:0050767
1537	GO:0042503
1538	GO:0051259
1539	GO:0000922
1540	GO:0019838
1541	GO:0055082
1542	GO:0051592
1543	GO:0002053
1544	GO:0032945
1545	GO:0005083
1546	GO:0008633
1547	GO:0009310
1548	GO:0006873
1549	GO:0010464
1550	GO:0070664
1551	GO:0001964
1552	GO:0044463
1553	AA_composition_M_mean
1554	GO:0060079
1555	GO:0010553
1556	GO:0050672
1557	GO:0002376
1558	GO:0022402
1559	in_locta_density_0.7_mean
1560	GO:0019842
1561	AA_composition_R_max
1562	GO:0051170
1563	GO:0044448

1564	GO:0050852
1565	GO:0009755
1566	GO:0019725
1567	GO:0071636
1568	GO:0019003
1569	GO:0000278
1570	GO:0006606
1571	GO:0065008
1572	GO:0043280
1573	GO:0046625
1574	GO:0007346
1575	GO:0000188
1576	GO:0060562
1577	GO:0010467
1578	GO:0032963
1579	GO:0003924
1580	GO:0051046
1581	GO:0045321
1582	GO:0017134
1583	GO:0042573
1584	GO:0010952
1585	AA_composition_L_max
1586	GO:0010563
1587	GO:0051223
1588	GO:0016331
1589	GO:0040018
1590	GO:0002507
1591	GO:0017157
1592	GO:0046635
1593	GO:0071495
1594	GO:0048598
1595	GO:0045936
1596	GO:0009306
1597	GO:0034621
1598	GO:0050793
1599	GO:0032870
1600	GO:0043623
1601	polarity_distribution_N.0.5_mean
1602	GO:0002683
1603	GO:0034622
1604	GO:0032372
1605	GO:0051216
1606	GO:0007260
1607	GO:0002440
1608	GO:0001958
1609	GO:0045648
1610	GO:0032375

1611	GO:0043534
1612	GO:0006975
1613	GO:0030183
1614	GO:0031821
1615	GO:0060795
1616	GO:0031110
1617	GO:0032376
1618	GO:0005520
1619	GO:0050671
1620	GO:0045309
1621	GO:0014910
1622	GO:0032331
1623	GO:0010810
1624	GO:0031109
1625	GO:0051649
1626	GO:0032373
1627	GO:0010720
1628	GO:0031994
1629	GO:0032946
1630	GO:0031982
1631	GO:0031558
1632	GO:0019048
1633	GO:0007131
1634	GO:0070665
1635	GO:0032332
1636	GO:0031557
1637	GO:0048341
1638	GO:0060323
1639	GO:0021533
1640	GO:0051171
1641	GO:0070491
1642	GO:0060325
1643	GO:0030099
1644	GO:0042501
1645	GO:0031329
1646	GO:0042228
1647	GO:0048729
1648	GO:0060322
1649	GO:0060078
1650	GO:0004714
1651	GO:0045622
1652	GO:0060090
1653	GO:0051220
1654	GO:0043395
1655	GO:0031100
1656	GO:0060324
1657	GO:0045624

1658	GO:0060081
1659	GO:0043388
1660	GO:0032633
1661	GO:0005178
1662	GO:0001704
1663	GO:0006919
1664	GO:0032673
1665	second_singular_values
1666	GO:0071634
1667	GO:0060249
1668	GO:0006936
1669	GO:0005158
1670	GO:0071604
1671	GO:0002720
1672	GO:0042506
1673	GO:0010843
1674	secondary_structure_transition_NH_max
1675	GO:0002573
1676	GO:0016358
1677	polarity_composition_N_mean
1678	GO:0004716
1679	GO:0032268
1680	GO:0044212
1681	GO:0016810
1682	GO:0065007
1683	GO:0016573
1684	GO:0010869
1685	GO:0030539
1686	GO:0017048
1687	GO:0050708
1688	GO:0031663
1689	GO:0045061
1690	GO:0030424
1691	GO:0051918
1692	GO:0070822
1693	GO:0008384
1694	GO:0042562
1695	GO:0030111
1696	GO:0051917
1697	GO:0023046
1698	GO:0051023
1699	GO:0001525
1700	GO:0016580
1701	GO:0023060
1702	GO:0032640
1703	in_locta_density_0.3_mean
1704	GO:0030947

1705	GO:0050794
1706	polarizability_distribution_H.0.0_max
1707	GO:0030291
1708	GO:0043254
1709	GO:0044270
1710	GO:0001657
1711	GO:0016853
1712	graph_density
1713	GO:0010224
1714	GO:0004468
1715	GO:0032680
1716	GO:0042226
1717	GO:0031702
1718	GO:0005088
1719	GO:0006310
1720	GO:0004402
1721	GO:0005516
1722	GO:0031072
1723	GO:0048678
1724	GO:0000166
1725	GO:0000086
1726	GO:0002011
1727	GO:0009108
1728	GO:0060205
1729	GO:0031295
1730	VanDerWaal_distribution_H.0.0_max
1731	GO:0030521
1732	GO:0016830
1733	GO:0001909
1734	GO:0045686
1735	GO:0048706
1736	solvent_accessibility_transition_HE_mean
1737	GO:0031983
1738	GO:0016758
1739	secondary_structure_distribution_H.0.5_mean
1740	GO:0030888
1741	GO:0014014
1742	GO:0001906
1743	GO:0031294
1744	GO:0043436
1745	GO:0002712
1746	GO:0009057
1747	GO:0045577
1748	GO:0032874
1749	GO:0030030
1750	GO:0016757
1751	GO:0060393

1752	polarity_distribution_P.0.0_max
1753	GO:0030170
1754	GO:0051090
1755	GO:0032991
1756	VanDerWaal_transition_NH_mean
1757	GO:0042787
1758	GO:0002889
1759	GO:0045581
1760	GO:0044433
1761	VanDerWaal_distribution_N.0.25_mean
1762	GO:0048663
1763	GO:0006082
1764	GO:0006351
1765	GO:0042493
1766	GO:0031345
1767	GO:0006275
1768	GO:0051101
1769	GO:0006732
1770	GO:0032872
1771	AA_composition_Y_mean
1772	GO:0046847
1773	GO:0030010
1774	GO:0070279
1775	GO:0000940
1776	GO:0002703
1777	GO:0033273
1778	GO:0018108
1779	GO:0004857
1780	GO:0006355
1781	GO:0070252
1782	GO:0045732
1783	GO:0000118
1784	GO:0090046
1785	GO:0060389
1786	GO:0033275
1787	GO:0019752
1788	hydrophobicity_distribution_H.0.0_max
1789	GO:0018212
1790	GO:0001823
1791	GO:0009309
1792	GO:0030049
1793	GO:0016581
1794	GO:0048260
1795	GO:0032845
1796	GO:0051602
1797	solvent_accessibility_distribution_H.0.25_mean
1798	GO:0071158

1799	GO:0016836
1800	GO:0044283
1801	GO:0046888
1802	GO:0006977
1803	GO:0046638
1804	GO:0042180
1805	GO:0065003
1806	GO:0010812
1807	GO:0006911
1808	GO:0003824
1809	GO:0046637
1810	GO:0016835
1811	GO:0043933
1812	GO:0001547
1813	GO:0042523
1814	polarity_distribution_H.0.5_max
1815	GO:0035088
1816	polarity_transition_NH_mean
1817	GO:0035108
1818	GO:0033344
1819	GO:0001666
1820	GO:0045885
1821	polarizability_transition_PN_mean
1822	GO:0051287
1823	GO:0035107
1824	GO:0016504
1825	weight_edge_variance.without_missing_edge.
1826	GO:0046668
1827	GO:0048675
1828	GO:0045177
1829	GO:0048736
1830	GO:0045682
1831	GO:0010741
1832	GO:0050998
1833	GO:0007431
1834	GO:0009063
1835	GO:0002209
1836	GO:0050789
1837	GO:0010092
1838	GO:0070513
1839	GO:0042101
1840	GO:0060173
1841	GO:0045684
1842	GO:0060134
1843	GO:0019219
1844	GO:0010463
1845	GO:0042770

1846	GO:0055007
1847	GO:0045598
1848	GO:0072175
1849	GO:0001662
1850	GO:0002544
1851	GO:0030426
1852	weight_edge_variance.with_missing_edge.
1853	GO:0002456
1854	GO:0050907
1855	solvent_accessibility_distribution_H.1.0_max
1856	GO:0032270
1857	GO:0035148
1858	GO:0050913
1859	GO:0050730
1860	GO:0003712
1861	GO:0051247
1862	GO:0021545
1863	GO:0002674
1864	GO:0070411
1865	VanDerWaal_composition_P_mean
1866	GO:0001838
1867	GO:0022604
1868	GO:0042306
1869	GO:0015837
1870	GO:0031344
1871	VanDerWaal_distribution_N.0.25_max
1872	GO:0006308
1873	secondary_structure_distribution_N.0.75_mean
1874	GO:0045821
1875	GO:0002052
1876	GO:0032862
1877	GO:0060396
1878	AA_composition_A_max
1879	GO:0045625
1880	GO:0009889
1881	GO:0032856
1882	GO:0042307
1883	GO:0045841
1884	GO:0005509
1885	GO:0071378
1886	GO:0031090
1887	GO:0010556
1888	GO:0002825
1889	GO:0046332
1890	GO:0030838
1891	GO:0071174
1892	GO:0016746

1893	GO:0001501
1894	GO:0060416
1895	GO:0031625
1896	GO:0005085
1897	GO:0031577
1898	GO:0032956
1899	GO:0031326
1900	GO:0051656
1901	GO:0071173
1902	GO:0001708
1903	GO:0017038
1904	GO:0030071
1905	GO:0010940
1906	GO:0048636
1907	GO:0006767
1908	GO:0007094
1909	GO:0002768
1910	GO:0045844
1911	secondary_structure_distribution_N.0.5_mean
1912	GO:0000255
1913	GO:0051098
1914	GO:0009948
1915	GO:0046983
1916	GO:0007262
1917	GO:0051704
1918	GO:0046328
1919	GO:0046449
1920	GO:0048643
1921	GO:0090047
1922	in_locta_density_0.2_mean
1923	GO:0031103
1924	GO:0035282
1925	GO:0002040
1926	GO:0005161
1927	GO:0033327
1928	GO:0051091
1929	GO:0050801
1930	GO:0006972
1931	GO:0007405
1932	GO:0000578
1933	GO:0010623
1934	GO:0008307
1935	GO:0032103
1936	GO:0002088
1937	GO:0044058
1938	GO:0016460
1939	secondary_structure_composition_H_max

1940	GO:0000781
1941	GO:0048565
1942	GO:0005859
1943	GO:0003713
1944	GO:0031264
1945	GO:0001836
1946	GO:0007435
1947	GO:0032269
1948	GO:0048010
1949	GO:0030496
1950	GO:0048710
1951	polarity_distribution_P.1.0_mean
1952	GO:0045414
1953	GO:0051148
1954	GO:0004869
1955	GO:0034405
1956	GO:0007369
1957	GO:0045416
1958	GO:0006471
1959	GO:0045588
1960	GO:0009266
1961	GO:0030330
1962	GO:0060158
1963	GO:0002637
1964	GO:0002369
1965	GO:0045586
1966	polarizability_transition_PN_max
1967	GO:0019199
1968	GO:0032743
1969	GO:0046645
1970	GO:0030574
1971	GO:0042104
1972	GO:0005198
1973	GO:0048610
1974	GO:0002726
1975	GO:0032677
1976	GO:0007176
1977	GO:0046643
1978	GO:0042345
1979	GO:0045184
1980	GO:0043410
1981	GO:0018209
1982	GO:0043967
1983	GO:0010469
1984	GO:0002724
1985	GO:0042348
1986	GO:0051482

1987	GO:0048814
1988	secondary_structure_distribution_P.0.75_mean
1989	GO:0002377
1990	GO:0001658
1991	GO:0010595
1992	GO:0043565
1993	GO:0045926
1994	GO:0007389
1995	GO:0050773
1996	hydrophobicity_composition_P_mean
1997	GO:0002697
1998	GO:0035272
1999	GO:0043170
2000	GO:0060675
2001	GO:0044060
2002	GO:0033198
2003	GO:0000060
2004	GO:0018107
2005	GO:0006937
2006	GO:0048812
2007	GO:0031233
2008	GO:0007250
2009	GO:0016628
2010	GO:0002698
2011	GO:0061025
2012	GO:0006725
2013	GO:0016604
2014	GO:0010560
2015	GO:0005771
2016	GO:0006944
2017	GO:0051281
2018	GO:0014812
2019	GO:0004705
2020	GO:0030308
2021	hydrophobicity_distribution_H.1.0_mean
2022	GO:0004709
2023	GO:0002757
2024	GO:0009629
2025	GO:0050765
2026	GO:0014909
2027	GO:0002700
2028	GO:0000165
2029	GO:0051480
2030	GO:0000267
2031	GO:0043525
2032	GO:0002764
2033	GO:0021695

2034	GO:0002645
2035	polarizability_distribution_P.0.25_mean
2036	GO:0006040
2037	GO:0002443
2038	GO:0033036
2039	GO:0046395
2040	GO:0043497
2041	GO:0005886
2042	GO:0019787
2043	GO:0004721
2044	GO:0042113
2045	GO:0021696
2046	GO:0001773
2047	out_clustering_max
2048	GO:0008210
2049	GO:0002643
2050	GO:0030595
2051	GO:0002221
2052	secondary_structure_distribution_P.0.5_mean
2053	GO:0051289
2054	GO:0016054
2055	GO:0042308
2056	GO:0030169
2057	GO:0070603
2058	GO:0005975
2059	GO:0009055
2060	GO:0001775
2061	GO:0042992
2062	GO:0016514
2063	GO:0046850
2064	GO:0003700
2065	GO:0015026
2066	GO:0042994
2067	GO:0016197
2068	GO:0045124
2069	GO:0048332
2070	GO:0051055
2071	GO:0033205
2072	in_out_topological_max
2073	GO:0032835
2074	GO:0033764
2075	GO:0002706
2076	GO:0016638
2077	GO:0009820
2078	out_locta_density_0.4_mean
2079	GO:0072006
2080	GO:0000149

2081	GO:0023056
2082	GO:0016229
2083	GO:0031293
2084	GO:0043197
2085	GO:0051428
2086	GO:0019748
2087	GO:0030238
2088	GO:0030659
2089	GO:0031254
2090	GO:0009967
2091	GO:0051260
2092	GO:0051301
2093	GO:0001931
2094	GO:0044309
2095	GO:0030252
2096	GO:0010657
2097	GO:0050778
2098	GO:0051149
2099	GO:0048588
2100	GO:0033005
2101	GO:0043586
2102	GO:0051489
2103	GO:0006875
2104	VanDerWaal_distribution_N.0.5_max
2105	GO:0016453
2106	GO:0010660
2107	GO:0032148
2108	GO:0051491
2109	GO:0007163
2110	in_out_topological_mean
2111	GO:0005099
2112	GO:0045792
2113	GO:0032273
2114	GO:0015031
2115	GO:0042645
2116	GO:0046890
2117	GO:0043277
2118	AA_composition_Y_max
2119	GO:0051252
2120	GO:0032957
2121	GO:0006470
2122	GO:0009295
2123	GO:0046530
2124	GO:0043536
2125	GO:0006874
2126	GO:0006021
2127	GO:0034612

2128	GO:0008630
2129	GO:0055074
2130	polarity_transition_PN_max
2131	GO:0030001
2132	GO:0043647
2133	GO:0043501
2134	GO:0050854
2135	GO:0014888
2136	GO:0042990
2137	GO:0006626
2138	GO:0030949
2139	GO:0008156
2140	out_locta_density_0.7_mean
2141	GO:0070585
2142	GO:0042991
2143	GO:0006865
2144	GO:0032094
2145	GO:0070304
2146	GO:0042472
2147	GO:0010226
2148	AA_composition_H_mean
2149	GO:0008284
2150	GO:0051588
2151	GO:0008013
2152	GO:0008083
2153	GO:0031584
2154	GO:0007252
2155	GO:0032735
2156	GO:0051781
2157	GO:0070302
2158	GO:0006474
2159	GO:0033256
2160	GO:0006766
2161	GO:0048302
2162	GO:0014823
2163	GO:0006509
2164	GO:0019400
2165	GO:0007442
2166	GO:0018409
2167	GO:0051293
2168	GO:0048304
2169	GO:0005246
2170	GO:0010948
2171	GO:0051653
2172	GO:0018076
2173	GO:0016772
2174	GO:0048291

2175	GO:0007159
2176	GO:0031365
2177	GO:0051248
2178	VanDerWaal_distribution_N.0.0_mean
2179	GO:0045664
2180	GO:0005730
2181	GO:0014896
2182	GO:0032091
2183	GO:0018394
2184	GO:0007090
2185	GO:0050662
2186	GO:0033627
2187	GO:0031410
2188	GO:0042044
2189	GO:0003002
2190	GO:0008045
2191	GO:0051707
2192	GO:0046426
2193	GO:0070587
2194	GO:0051817
2195	GO:0009615
2196	GO:0005834
2197	GO:0042532
2198	GO:0048037
2199	GO:0044003
2200	GO:0010470
2201	GO:0005624
2202	GO:0046649
2203	GO:0050710
2204	GO:0048008
2205	GO:0021987
2206	GO:0045449
2207	GO:0002664
2208	GO:0003007
2209	GO:0032970
2210	GO:0060048
2211	polarity_transition_PH_mean
2212	GO:0021544
2213	GO:0002374
2214	GO:0002517
2215	GO:0009303
2216	GO:0060070
2217	GO:0002666
2218	GO:0002740
2219	GO:0005159
2220	GO:0034199
2221	GO:0021756

2222	GO:0048365
2223	GO:0070586
2224	GO:0050810
2225	GO:0034114
2226	GO:0022614
2227	GO:0050819
2228	GO:0050896
2229	GO:0042100
2230	GO:0048407
2231	GO:0010523
2232	GO:0005096
2233	GO:0016407
2234	GO:0002739
2235	GO:0050881
2236	GO:0030195
2237	GO:0007049
2238	GO:0042993
2239	GO:0000904
2240	GO:0001784
2241	GO:0009880
2242	GO:0050879
2243	out_local_density_0.2_max
2244	GO:0051806
2245	GO:0005234
2246	GO:0030278
2247	GO:0046718
2248	GO:0005230
2249	GO:0032321
2250	GO:0019222
2251	GO:0005231
2252	GO:0050431
2253	polarizability_distribution_P.0.0_mean
2254	GO:0052192
2255	GO:0031901
2256	AA_composition_H_max
2257	GO:0046838
2258	GO:0051828
2259	GO:0006461
2260	GO:0003001
2261	GO:0046855
2262	GO:0001818
2263	GO:0044409
2264	GO:0051537
2265	GO:0032147
2266	GO:0045471
2267	GO:0051238
2268	VanDerWaal_distribution_P.0.25_mean

2269	GO:0070271
2270	GO:0030260
2271	GO:0042490
2272	GO:0042508
2273	hydrophobicity_distribution_H.0.75_mean
2274	GO:0032799
2275	GO:0052126
2276	GO:0006790
2277	GO:0003085
2278	secondary_structure_distribution_P.0.0_mean
2279	GO:0044421
2280	GO:0070265
2281	GO:0048232
2282	polarizability_distribution_N.0.5_max
2283	GO:0051092
2284	GO:0018193
2285	GO:0060485
2286	GO:0030552
2287	polarizability_distribution_H.0.5_mean
2288	GO:0045773
2289	GO:0007283
2290	AA_composition_I_mean
2291	GO:0042634
2292	GO:0001502
2293	GO:0030528
2294	GO:0021575
2295	GO:0042175
2296	GO:0051797
2297	GO:0048863
2298	GO:0045740
2299	GO:0030665
2300	GO:0002335
2301	GO:0001910
2302	GO:0043271
2303	GO:0042542
2304	GO:0006952
2305	GO:0051798
2306	GO:0005789
2307	VanDerWaal_distribution_N.1.0_mean
2308	VanDerWaal_distribution_H.0.5_mean
2309	GO:0009066
2310	GO:0031341
2311	GO:0006511
2312	GO:0042375
2313	GO:0046488
2314	in_local_density_0.8_max
2315	GO:0006887

2316	GO:0006929
2317	GO:0030852
2318	GO:0007165
2319	GO:0001709
2320	GO:0075136
2321	GO:0030851
2322	GO:0005783
2323	GO:0005518
2324	GO:0010544
2325	GO:0050729
2326	GO:0006536
2327	GO:0032102
2328	GO:0008033
2329	GO:0052173
2330	GO:0043027
2331	GO:0045778
2332	GO:0043114
2333	GO:0005041
2334	GO:0052200
2335	GO:0044267
2336	GO:0019888
2337	GO:0005123
2338	GO:0042135
2339	GO:0019208
2340	GO:0090100
2341	GO:0030017
2342	GO:0019538
2343	GO:0048666
2344	GO:0045123
2345	GO:0045661
2346	GO:0031175
2347	GO:0007254
2348	GO:0046427
2349	GO:0060390
2350	GO:0008483
2351	GO:0051924
2352	GO:0016500
2353	GO:0042977
2354	GO:0007184
2355	GO:0042346
2356	in_locta_density_0_mean
2357	GO:0042531
2358	GO:0005160
2359	GO:0042517
2360	GO:0045296
2361	GO:0007214
2362	GO:0007259

2363	GO:0034713
2364	GO:0031111
2365	GO:0050864
2366	GO:0050772
2367	GO:0031638
2368	GO:0060391
2369	GO:0008625
2370	GO:0030866
2371	GO:0031114
2372	GO:0003985
2373	GO:0033160
2374	VanDerWaal_distribution_H.1.0_mean
2375	GO:0030865
2376	GO:0043496
2377	GO:0030141
2378	GO:0004029
2379	GO:0030326
2380	GO:0032755
2381	GO:0033158
2382	out_locta_density_0.9_mean
2383	GO:0043034
2384	GO:0007019
2385	solvent_accessibility_distribution_H.0.75_mean
2386	GO:0010717
2387	GO:0007191
2388	GO:0035113
2389	GO:0007026
2390	GO:0050663
2391	GO:0010770
2392	GO:0006302
2393	in_locta_density_0.1_mean
2394	GO:0048738
2395	GO:0007212
2396	GO:0001912
2397	GO:0010718
2398	GO:0023050
2399	GO:0032909
2400	polarizability_distribution_H.1.0_mean
2401	GO:0022406
2402	GO:0007179
2403	GO:0045058
2404	GO:0006629
2405	GO:0003009
2406	GO:0035136
2407	GO:0035091
2408	polarizability_distribution_P.0.5_mean
2409	GO:0032906

2410	GO:0043154
2411	GO:0004702
2412	polarity_distribution_P.0.75_mean
2413	GO:0010466
2414	GO:0035383
2415	GO:0045954
2416	GO:0030522
2417	GO:0046717
2418	GO:0021535
2419	GO:0006637
2420	GO:0002715
2421	GO:0043487
2422	GO:0051347
2423	GO:0001653
2424	GO:0042269
2425	out_locta_density_0.3_mean
2426	GO:0040011
2427	GO:0017124
2428	GO:0010953
2429	GO:0010769
2430	GO:0046425
2431	GO:0016863
2432	GO:0008528
2433	VanDerWaal_distribution_P.0.0_mean
2434	GO:0033674
2435	GO:0045410
2436	GO:0010955
2437	GO:0002717
2438	GO:0046889
2439	GO:0044243
2440	GO:0002548
2441	GO:0070613
2442	GO:0045408
2443	GO:0048568
2444	GO:0031343
2445	GO:0045055
2446	GO:0043467
2447	GO:0042219
2448	GO:0030673
2449	GO:0016788
2450	GO:0003823
2451	GO:0051054
2452	GO:0001707
2453	GO:0030667
2454	GO:0032526
2455	GO:0070064
2456	GO:0007631

2457	GO:0021532
2458	GO:0007599
2459	GO:0051082
2460	GO:0044282
2461	GO:0034605
2462	secondary_structure_distribution_N.1.0_mean
2463	GO:0051879
2464	GO:0050878
2465	GO:0007584
2466	GO:0042552
2467	GO:0050817
2468	GO:0016505
2469	GO:0016042
2470	in_locta_density_0.4_mean
2471	GO:0007596
2472	GO:0060401
2473	GO:0031401
2474	GO:0008656
2475	GO:0016585
2476	GO:0005929
2477	GO:0060402
2478	GO:0043028
2479	GO:0042327
2480	GO:0045112
2481	GO:0051176
2482	GO:0045937
2483	GO:0004222
2484	GO:0048015
2485	GO:0010562
2486	GO:0033137
2487	GO:0008093
2488	polarizability_distribution_N.0.5_mean
2489	GO:0051590
2490	GO:0009620
2491	GO:0001913
2492	GO:0046884
2493	GO:0043412
2494	GO:0032274
2495	GO:0001914
2496	topological_change_0.2_0.3
2497	GO:0030901
2498	GO:0010799
2499	VanDerWaal_composition_H_max
2500	GO:0045666
2501	GO:0002026
2502	GO:0006879
2503	GO:0010800

2504	GO:0051702
2505	GO:0060021
2506	GO:0044452
2507	GO:0007257
2508	GO:0030177
2509	GO:0007166
2510	GO:0031323
2511	GO:0031093
2512	polarizability_composition_H_max
2513	GO:0023033
2514	GO:0051881
2515	out_clustering_mean
2516	GO:0050856
2517	GO:0070167
2518	GO:0031091
2519	GO:0005794
2520	GO:0060688
2521	GO:0048844
2522	GO:0030500
2523	GO:0060191
2524	GO:0032587
2525	GO:0045860
2526	GO:0060840
2527	GO:0051048
2528	GO:0010043
2529	GO:0001755
2530	GO:0044236
2531	AA_composition_V_max
2532	GO:0016860
2533	GO:0010627
2534	GO:0043039
2535	hydrophobicity_composition_N_mean
2536	GO:0009607
2537	GO:0004435
2538	GO:0032271
2539	GO:0033209
2540	GO:0016876
2541	GO:0048864
2542	GO:0043011
2543	GO:0005981
2544	GO:0004434
2545	GO:0043331
2546	GO:0043038
2547	GO:0015918
2548	GO:0015630
2549	GO:0019827
2550	GO:0006706

2551	GO:0004629
2552	GO:0001935
2553	GO:0030151
2554	GO:0034660
2555	GO:0030301
2556	GO:0044262
2557	GO:0001936
2558	GO:0010740
2559	GO:0031672
2560	GO:0004812
2561	GO:0051047
2562	GO:0034655
2563	GO:0015276
2564	GO:0001764
2565	GO:0006399
2566	GO:0034656
2567	GO:0022834
2568	GO:0008339
2569	GO:0045824
2570	GO:0032494
2571	GO:0016875
2572	GO:0032635
2573	GO:0009225
2574	GO:0034391
2575	GO:0070838
2576	GO:0014047
2577	hydrophobicity_distribution_H.0.0_mean
2578	GO:0007253
2579	GO:0051053
2580	GO:0051043
2581	GO:0006418
2582	GO:0048505
2583	GO:0015674
2584	GO:0034390
2585	GO:0030509
2586	GO:0007494
2587	GO:0014048
2588	GO:0042347
2589	GO:0008305
2590	GO:0040034
2591	GO:0032675
2592	GO:0006041
2593	GO:0006816
2594	GO:0005066
2595	GO:0060041
2596	GO:0045190
2597	GO:0016846

2598	GO:0051044
2599	GO:0009636
2600	GO:0042713
2601	GO:0006044
2602	GO:0007320
2603	GO:0042518
2604	GO:0016447
2605	GO:0050771
2606	VanDerWaal_distribution_H.0.0_mean
2607	GO:0033574
2608	GO:0002204
2609	GO:0048771
2610	GO:0002562
2611	GO:0051258
2612	GO:0016769
2613	GO:0043383
2614	GO:0002381
2615	GO:0045060
2616	GO:0043208
2617	polarizability_distribution_H.0.0_mean
2618	GO:0031098
2619	GO:0016445
2620	GO:0019958
2621	GO:0016790
2622	GO:0051861
2623	GO:0030262
2624	GO:0006720
2625	GO:0042802
2626	GO:0005138
2627	GO:0003012
2628	GO:0016444
2629	GO:0070925
2630	GO:0002089
2631	GO:0004497
2632	GO:0021915
2633	GO:0000737
2634	GO:0002200
2635	GO:0042577
2636	GO:0006997
2637	GO:0002224
2638	GO:0051302
2639	polarity_distribution_P.0.0_mean
2640	GO:0046914
2641	GO:0002208
2642	GO:0009067
2643	GO:0060348
2644	GO:0006309

2645	GO:0046839
2646	GO:0014003
2647	GO:0051899
2648	GO:0001780
2649	GO:0006464
2650	hydrophobicity_distribution_N.0.0_mean
2651	GO:0001781
2652	GO:0000775
2653	GO:0015459
2654	GO:0002710
2655	GO:0042471
2656	GO:0006071
2657	GO:0033008
2658	GO:0045589
2659	GO:0043603
2660	GO:0002820
2661	GO:0043306
2662	GO:0006538
2663	GO:0002823
2664	GO:0043302
2665	GO:0006576
2666	GO:0043123
2667	GO:0031092
2668	GO:0048525
2669	GO:0009065
2670	GO:0031000
2671	GO:0007015
2672	GO:0010647
2673	GO:0070555
2674	GO:0007530
2675	AA_composition_L_mean
2676	GO:0050886
2677	GO:0048305
2678	VanDerWaal_transition_PN_mean
2679	GO:0048854
2680	GO:0005131
2681	GO:0043499
2682	GO:0000768
2683	GO:0002062
2684	GO:0004518
2685	GO:0006949
2686	GO:0032787
2687	GO:0009798
2688	GO:0070053
2689	GO:0003706
2690	GO:0001894
2691	GO:0033261

2692	GO:0001974
2693	GO:0046532
2694	GO:0045650
2695	GO:0000792
2696	GO:0051966
2697	GO:0005249
2698	GO:0045713
2699	GO:0002792
2700	GO:0008201
2701	GO:0043122
2702	GO:0002279
2703	GO:0010871
2704	GO:0032052
2705	GO:0007249
2706	GO:0001955
2707	GO:0046907
2708	GO:0043303
2709	GO:0046639
2710	GO:0016408
2711	GO:0016597
2712	GO:0043084
2713	GO:0043299
2714	GO:0046627
2715	GO:0007143
2716	GO:0002448
2717	GO:0014855
2718	GO:0005132
2719	GO:0044241
2720	GO:0055017
2721	GO:0001503
2722	hydrophobicity_distribution_N.1.0_mean
2723	GO:0007368
2724	GO:0005007
2725	GO:0060038
2726	polarizability_distribution_N.0.75_mean
2727	GO:0005626
2728	GO:0048266
2729	GO:0050775
2730	GO:0045211
2731	GO:0060419
2732	GO:0044304
2733	GO:0006577
2734	GO:0050840
2735	GO:0050728
2736	hydrophobicity_distribution_N.0.5_mean
2737	GO:0009437
2738	GO:0032847

2739	GO:0043236
2740	GO:0045576
2741	GO:0015872
2742	GO:0010883
2743	GO:0042110
2744	GO:0043030
2745	GO:0002252
2746	GO:0033613
2747	GO:0032370
2748	GO:0017137
2749	GO:0006401
2750	GO:0006612
2751	GO:0046928
2752	GO:0051964
2753	GO:0003707
2754	GO:0006402
2755	GO:0001530
2756	out_out_topological_mean
2757	GO:0001504
2758	GO:0051961
2759	GO:0004879
2760	GO:0000139
2761	GO:0040023
2762	GO:0044431
2763	GO:0014033
2764	GO:0001754
2765	GO:0051647
2766	GO:0044427
2767	GO:0014032
2768	GO:0009299
2769	GO:0002367
2770	GO:0016311
2771	GO:0050829
2772	GO:0002718
2773	GO:0035115
2774	GO:0030005
2775	GO:0044451
2776	GO:0016655
2777	GO:0032731
2778	GO:0050871
2779	GO:0032823
2780	GO:0032732
2781	GO:0030300
2782	GO:0016053
2783	GO:0032825
2784	GO:0032990
2785	GO:0007194

2786	GO:0032095
2787	out_local_density_0_max
2788	GO:0001779
2789	GO:0048858
2790	GO:0032098
2791	GO:0031280
2792	polarizability_distribution_P.1.0_mean
2793	GO:0032816
2794	GO:0046394
2795	out_local_density_0.1_max
2796	GO:0001957
2797	GO:0032107
2798	GO:0032814
2799	GO:0001973
2800	GO:0034103
2801	GO:0060559
2802	GO:0048667
2803	GO:0050688
2804	GO:0007193
2805	GO:0032104
2806	GO:0006066
2807	GO:0004620
2808	GO:0032715
2809	GO:0046685
2810	GO:0035303
2811	AA_composition_R_mean
2812	GO:0030834
2813	GO:0042826
2814	GO:0051350
2815	GO:0005899
2816	GO:0032855
2817	GO:0030042
2818	GO:0055065
2819	GO:0045761
2820	in_locta_density_0.9_mean
2821	GO:0030003
2822	GO:0006473
2823	GO:0051952
2824	GO:0050482
2825	GO:0071071
2826	GO:0003714
2827	GO:0071072
2828	GO:0060037
2829	GO:0008016
2830	GO:0032155
2831	GO:0032272
2832	GO:0045766

2833	GO:0032774
2834	GO:0007352
2835	GO:0000146
2836	GO:0005179
2837	GO:0051693
2838	GO:0008610
2839	GO:0000159
2840	GO:0001508
2841	GO:0005634
2842	GO:0030837
2843	GO:0070412
2844	GO:0030835
2845	GO:0030662
2846	GO:0010524
2847	GO:0030898
2848	GO:0030902
2849	GO:0000045
2850	GO:0032153
2851	GO:0032649
2852	GO:0004842
2853	GO:0016236
2854	GO:0032609
2855	GO:0006950
2856	GO:0008198
2857	GO:0017046
2858	GO:0050715
2859	GO:0030518
2860	GO:0046112
2861	GO:0031012
2862	GO:0016337
2863	GO:0002320
2864	GO:0005911
2865	GO:0005201
2866	GO:0055056
2867	GO:0008017
2868	GO:0007040
2869	GO:0007276
2870	GO:0009953
2871	GO:0016854
2872	GO:0048859
2873	GO:0046113
2874	GO:0005578
2875	GO:0033138
2876	GO:0060425
2877	in_clustering_max
2878	GO:0003774
2879	GO:0021543

2880	GO:0042596
2881	GO:0000185
2882	GO:0016459
2883	GO:0048713
2884	GO:0007052
2885	GO:0016866
2886	AA_composition_G_mean
2887	GO:0006110
2888	GO:0015020
2889	GO:0048535
2890	GO:0031400
2891	GO:0005795
2892	GO:0042398
2893	GO:0004687
2894	GO:0006730
2895	GO:0005802
2896	GO:0014819
2897	GO:0005148
2898	hydrophobicity_distribution_P.0.25_mean
2899	GO:0060591
2900	GO:0046676
2901	GO:0051346
2902	GO:0017144
2903	GO:0006733
2904	GO:0090278
2905	GO:0009410
2906	GO:0006575
2907	GO:0045723
2908	GO:0001649
2909	GO:0016192
2910	GO:0000819
2911	GO:0031985
2912	GO:0071466
2913	GO:0045669
2914	GO:0042133
2915	GO:0051983
2916	GO:0060033
2917	GO:0044432
2918	GO:0000279
2919	GO:0016289
2920	GO:0000718
2921	GO:0031984
2922	GO:0030658
2923	GO:0001975
2924	GO:0030041
2925	GO:0006805
2926	GO:0000070

2927	GO:0001959
2928	GO:0000087
2929	secondary_structure_composition_N_mean
2930	GO:0046496
2931	GO:0008154
2932	GO:0007067
2933	GO:0071212
2934	GO:0000280
2935	GO:0008347
2936	GO:0042058
2937	GO:0048285
2938	GO:0007034
2939	GO:0016641
2940	GO:0006769
2941	GO:0046642
2942	GO:0016567
2943	GO:0042805
2944	GO:0005506
2945	GO:0007041
2946	GO:0090092
2947	GO:0007200
2948	GO:0004303
2949	GO:0045446
2950	GO:0070330
2951	GO:0042136
2952	GO:0002250
2953	GO:0043279
2954	GO:0003158
2955	GO:0002460
2956	GO:0006721
2957	GO:0046173
2958	GO:0030159
2959	GO:0051409
2960	hydrophobicity_distribution_N.0.75_mean
2961	GO:0019915
2962	GO:0043589
2963	GO:0045348
2964	GO:0018149
2965	GO:0008652
2966	out_clustering_variance
2967	out_in_topological_variance
2968	GO:0048704
2969	GO:0005577
2970	GO:0045059
2971	GO:0033293
2972	GO:0005768
2973	GO:0034374

2974	GO:0042056
2975	GO:0006940
2976	GO:0003988
2977	GO:0030431
2978	GO:0045715
2979	GO:0032801
2980	GO:0045714
2981	GO:0000062
2982	GO:0043471
2983	GO:0045823
2984	GO:0043470
2985	secondary_structure_distribution_P.0.25_mean
2986	GO:0003857
2987	GO:0032947
2988	GO:0045197
2989	GO:0004300
2990	GO:0051179
2991	GO:0014031
2992	GO:0018210
2993	GO:0050702
2994	GO:0050706
2995	GO:0050701
2996	GO:0006350
2997	GO:0048762
2998	GO:0050704
2999	GO:0009143
3000	GO:0010743
3001	GO:0019229
3002	GO:0007016
3003	polarizability_distribution_N.0.25_mean
3004	GO:0044255
3005	GO:0045351
3006	GO:0031640
3007	GO:0006935
3008	GO:0007059
3009	GO:0042330
3010	GO:0090257
3011	topological_change_0.5_0.6
3012	GO:0043130
3013	GO:0002076
3014	GO:0032182
3015	GO:0022037
3016	GO:0032429
3017	GO:0030004
3018	GO:0050818
3019	GO:0030955
3020	GO:0048701

3021	GO:0008509
3022	GO:0021522
3023	GO:0071156
3024	GO:0006549
3025	GO:0005253
3026	GO:0033860
3027	GO:0032430
3028	GO:0009855
3029	GO:0048002
3030	GO:0021517
3031	out_in_topological_mean
3032	GO:0034399
3033	GO:0009799
3034	AA_composition_W_max
3035	GO:0016363
3036	GO:0019530
3037	GO:0046697
3038	GO:0007126
3039	polarity_distribution_N.0.75_mean
3040	GO:0051327
3041	GO:0051321
3042	GO:0006573
3043	GO:0016324
3044	GO:0033003
3045	GO:0001893
3046	GO:0021537
3047	GO:0070647
3048	GO:0032446
3049	GO:0021587
3050	GO:0015057
3051	GO:0005026
3052	GO:0030971
3053	GO:0021871
3054	GO:0005024
3055	GO:0042762
3056	GO:0001710
3057	GO:0032927
3058	GO:0030516
3059	GO:0048333
3060	GO:0007178
3061	GO:0008565
3062	GO:0043543
3063	GO:0017002
3064	GO:0030501
3065	GO:0003016
3066	out_locta_density_0.8_mean
3067	GO:0016361

3068	GO:0006338
3069	GO:0070169
3070	GO:0050542
3071	GO:0005025
3072	GO:0008076
3073	GO:0046696
3074	GO:0050543
3075	GO:0048179
3076	GO:0034705
3077	GO:0031532
3078	GO:0004675
3079	GO:0015909
3080	GO:0044422
3081	GO:0001556
3082	GO:0030199
3083	GO:0030318
3084	GO:0006921
3085	out_local_density_0.3_max
3086	GO:0015082
3087	GO:0050931
3088	GO:0008138
3089	polarizability_distribution_N.1.0_mean
3090	GO:0000785
3091	GO:0051235
3092	GO:0060113
3093	GO:0016667
3094	GO:0043408
3095	GO:0007157
3096	GO:0007202
3097	GO:0002839
3098	GO:0010863
3099	GO:0002836
3100	GO:0030101
3101	GO:0002833
3102	GO:0002837
3103	GO:0042587
3104	GO:0002834
3105	GO:0031050
3106	GO:0002855
3107	GO:0009746
3108	GO:0002858
3109	GO:0017015
3110	GO:0030146
3111	GO:0002860
3112	GO:0034616
3113	GO:0002423
3114	GO:0070410

3115	GO:0008601
3116	GO:0002857
3117	GO:0006923
3118	GO:0005114
3119	GO:0034284
3120	GO:0002420
3121	GO:0048477
3122	GO:0031053
3123	polarity_distribution_P.1.0_max
3124	GO:0035304
3125	GO:0006922
3126	GO:0045667
3127	in_local_density_0.6_max
3128	GO:0005072
3129	GO:0050919
3130	GO:0009749
3131	GO:0031047
3132	GO:0006942
3133	GO:0009056
3134	GO:0035195
3135	GO:0016725
3136	GO:0035035
3137	GO:0016441
3138	GO:0016050
3139	GO:0017156
3140	GO:0016811
3141	GO:0007183
3142	GO:0050660
3143	GO:0042383
3144	GO:0071359
3145	polarity_distribution_H.0.0_mean
3146	GO:0019932
3147	GO:0030618
3148	GO:0019438
3149	GO:0007626
3150	GO:0005071
3151	GO:0002831
3152	out_locta_density_0.6_mean
3153	GO:0055072
3154	GO:0022029
3155	GO:0035194
3156	GO:0021885
3157	GO:0035196
3158	GO:0004551
3159	GO:0031581
3160	GO:0043161
3161	GO:0070918

3162	GO:0031589
3163	GO:0010498
3164	GO:0031201
3165	GO:0004527
3166	GO:0048193
3167	GO:0050748
3168	GO:0042581
3169	GO:0051547
3170	GO:0006904
3171	GO:0005484
3172	GO:0007272
3173	GO:0001950
3174	GO:0030228
3175	GO:0051549
3176	GO:0042147
3177	GO:0006541
3178	GO:0006063
3179	GO:0005637
3180	GO:0048278
3181	GO:0016081
3182	GO:0006888
3183	GO:0042159
3184	out_local_density_0.8_max
3185	GO:0008366
3186	GO:0019585
3187	GO:0007520
3188	GO:0051539
3189	GO:0042401
3190	GO:0009166
3191	GO:0044271
3192	GO:0016709
3193	GO:0030149
3194	GO:0047429
3195	GO:0006094
3196	GO:0030833
3197	GO:0019886
3198	polarity_distribution_P.0.5_mean
3199	polarizability_composition_P_mean
3200	GO:0042402
3201	GO:0002495
3202	GO:0008064
3203	GO:0008415
3204	GO:0002478
3205	GO:0032613
3206	GO:0048640
3207	VanDerWaal_distribution_H.1.0_max
3208	GO:0030832

3209	GO:0016458
3210	hydrophobicity_transition_PH_mean
3211	GO:0019884
3212	GO:0016409
3213	polarizability_distribution_H.1.0_max
3214	GO:0002886
3215	GO:0021904
3216	GO:0016747
3217	GO:0043507
3218	GO:0033135
3219	polarity_distribution_H.0.25_mean
3220	GO:0016705
3221	GO:0004553
3222	GO:0043506
3223	GO:0045933
3224	GO:0005102
3225	GO:0016798
3226	GO:0015144
3227	GO:0008378
3228	GO:0042734
3229	GO:0046689
3230	GO:0003676
3231	GO:0051119
3232	GO:0005782
3233	GO:0055094
3234	GO:0019439
3235	GO:0048662
3236	topological_change_0.1_0.2
3237	GO:0015145
3238	GO:0005525
3239	GO:0031907
3240	GO:0007620
3241	GO:0051789
3242	GO:0051640
3243	GO:0045453
3244	GO:0015149
3245	GO:0004175
3246	GO:0006739
3247	GO:0005355
3248	GO:0042737
3249	GO:0035250
3250	GO:0031214
3251	GO:0042537
3252	GO:0004601
3253	GO:0016684
3254	GO:0032580
3255	GO:0003677

3256	GO:0019362
3257	GO:0048521
3258	GO:0019905
3259	GO:0042277
3260	GO:0007411
3261	GO:0035270
3262	GO:0031420
3263	GO:0043526
3264	GO:0008306
3265	GO:0032501
3266	GO:0016712
3267	GO:0010884
3268	GO:0008131
3269	GO:0001756
3270	GO:0005737
3271	GO:0061053
3272	topological_change_0.4_0.5
3273	GO:0048668
3274	GO:0032760
3275	GO:0019825
3276	GO:0002418
3277	GO:0050780
3278	GO:0009595
3279	GO:0043516
3280	GO:0030219
3281	GO:0003254
3282	GO:0006069
3283	GO:0006067
3284	GO:0001916
3285	GO:0005501
3286	GO:0006974
3287	GO:0043255
3288	hydrophobicity_distribution_H.0.5_mean
3289	GO:0050839
3290	GO:0019840
3291	GO:0031639
3292	GO:0043195
3293	GO:0004024
3294	GO:0008066
3295	GO:0007409
3296	GO:0019228
3297	GO:0004022
3298	GO:0045161
3299	GO:0046883
3300	GO:0001554
3301	GO:0021511
3302	GO:0042803

3303	GO:0004745
3304	GO:0021513
3305	GO:0045779
3306	GO:0045923
3307	GO:0006979
3308	GO:0034308
3309	GO:0003300
3310	GO:0046851
3311	GO:0016101
3312	GO:0014743
3313	GO:0010611
3314	GO:0051882
3315	GO:0001523
3316	GO:0034104
3317	GO:0014897
3318	GO:0051900
3319	GO:0050770
3320	GO:0010517
3321	GO:0010518
3322	GO:0030104
3323	GO:0001824
3324	weight_edge_mean.without_missing_edge.
3325	GO:0060193
3326	GO:0031300
3327	GO:0050690
3328	GO:0032846
3329	GO:0007220
3330	weight_edge_mean.with_missing_edge.
3331	GO:0007033
3332	GO:0006631
3333	GO:0002032
3334	GO:0031348
3335	GO:0016742
3336	GO:0042362
3337	GO:0046686
3338	GO:0034708
3339	GO:0004372
3340	GO:0019934
3341	hydrophobicity_distribution_P.0.5_mean
3342	GO:0043167
3343	GO:0043588
3344	GO:0042304
3345	GO:0035097
3346	GO:0044260
3347	GO:0044272
3348	GO:0046872
3349	secondary_structure_transition_PN_mean

3350	GO:0006661
3351	GO:0043169
3352	GO:0032720
3353	GO:0060089
3354	GO:0006810
3355	GO:0004871
3356	GO:0016417
3357	GO:0071103
3358	GO:0006323
3359	GO:0050661
3360	GO:0044429
3361	GO:0001819
3362	GO:0030073
3363	GO:0042448
3364	GO:0042593
3365	GO:0016829
3366	GO:0043368
3367	GO:0019966
3368	GO:0033500
3369	GO:0032230
3370	GO:0044062
3371	GO:0051087
3372	GO:0050707
3373	GO:0000084
3374	GO:0002920
3375	GO:0004908
3376	GO:0051320
3377	GO:0005062
3378	GO:0008641
3379	GO:0008343
3380	GO:0016052
3381	GO:0045987
3382	GO:0030529
3383	GO:0060397
3384	GO:0000780
3385	GO:0000793
3386	GO:0051004
3387	GO:0000794
3388	GO:0021515
3389	GO:0007338
3390	GO:0008329
3391	in_out_topological_variance
3392	GO:0042474
3393	GO:0016298
3394	GO:0007160
3395	GO:0055102
3396	GO:0071715

3397	GO:0007229
3398	GO:0004679
3399	in_locta_density_0.8_mean
3400	GO:0045776
3401	VanDerWaal_composition_N_mean
3402	GO:0031588
3403	GO:0032309
3404	GO:0005173
3405	hydrophobicity_distribution_P.0.0_mean
3406	GO:0014075
3407	GO:0042439
3408	GO:0050435
3409	GO:0050680
3410	GO:0046320
3411	GO:0042834
3412	GO:0034332
3413	GO:0004197
3414	GO:0048712
3415	GO:0017053
3416	GO:0043304
3417	GO:0001825
3418	GO:0042755
3419	GO:0007292
3420	GO:0043300
3421	GO:0030850
3422	GO:0033006
3423	GO:0005694
3424	GO:0001542
3425	GO:0051354
3426	GO:0045931
3427	out_local_density_0.4_max
3428	GO:0015844
3429	GO:0006892
3430	GO:0012506
3431	GO:0007263
3432	GO:0030325
3433	GO:0007498
3434	GO:0016627
3435	GO:0002667
3436	GO:0010975
3437	GO:0008294
3438	GO:0043422
3439	GO:0030728
3440	GO:0002913
3441	GO:0051591
3442	GO:0042578
3443	GO:0002669

3444	GO:0022836
3445	GO:0008395
3446	GO:0002249
3447	GO:0001938
3448	GO:0048471
3449	GO:0042074
3450	GO:0019216
3451	GO:0016494
3452	GO:0005792
3453	GO:0005172
3454	GO:0002870
3455	GO:0030514
3456	GO:0042533
3457	polarizability_distribution_N.0.0_mean
3458	GO:0002911
3459	GO:0040015
3460	GO:0004528
3461	GO:0042440
3462	GO:0042534
3463	GO:0006518
3464	GO:0042598
3465	hydrophobicity_distribution_H.1.0_max
3466	GO:0042535
3467	GO:0002673
3468	GO:0070566
3469	GO:0031133
3470	GO:0023036
3471	GO:0004016
3472	GO:0046582
3473	GO:0000049
3474	in_degree_mean
3475	GO:0032536
3476	GO:0023049
3477	GO:0015012
3478	GO:0051349
3479	GO:0071157
3480	GO:0020037
3481	GO:0019221
3482	GO:0031281
3483	GO:0005539
3484	GO:0005874
3485	GO:0007018
3486	GO:0023038
3487	out_degree_mean
3488	GO:0042558
3489	GO:0046906
3490	GO:0051225

3491	GO:0046121
3492	GO:0046514
3493	in_clustering_mean
3494	GO:0007017
3495	GO:0016814
3496	GO:0009120
3497	GO:0008289
3498	GO:0005543
3499	GO:0042588
3500	GO:0009164
3501	GO:0003014
3502	GO:0008299
3503	GO:0019961
3504	GO:0016840
3505	GO:0043473
3506	GO:0004904
3507	GO:0005143
3508	GO:0030203
3509	GO:0042462
3510	GO:0032729
3511	in_local_density_0.5_max
3512	GO:0005184
3513	GO:0009069
3514	GO:0070723
3515	GO:0001890
3516	GO:0006208
3517	GO:0019935
3518	GO:0005149
3519	GO:0006022
3520	GO:0008328
3521	GO:0035254
3522	GO:0045026
3523	GO:0004970
3524	GO:0070498
3525	GO:0009112
3526	GO:0006734
3527	GO:0019859
3528	GO:0007187
3529	GO:0046466
3530	GO:0016208
3531	GO:0009074
3532	GO:0009116
3533	GO:0000097
3534	GO:0007188
3535	GO:0006210
3536	GO:0048247
3537	GO:0019933

3538	GO:0009068
3539	GO:0006633
3540	GO:0008168
3541	GO:0006749
3542	polarizability_distribution_P.1.0_max
3543	GO:0018065
3544	GO:0030147
3545	GO:0016741
3546	GO:0030545
3547	GO:0008589
3548	GO:0015925
3549	GO:0002467
3550	GO:0008202
3551	GO:0019377
3552	AA_composition_N_mean
3553	GO:0050714
3554	GO:0060443
3555	GO:0001829
3556	GO:0042773
3557	GO:0006558
3558	GO:0009611
3559	GO:0006641
3560	GO:0060056
3561	GO:0005996
3562	GO:0044450
3563	GO:0006572
3564	GO:0019865
3565	GO:0005773
3566	GO:0042775
3567	out_degree_correlation_max
3568	GO:0048873
3569	GO:0019674
3570	GO:0006559
3571	GO:0006090
3572	GO:0005885
3573	GO:0009100
3574	GO:0009154
3575	GO:0004602
3576	GO:0019318
3577	GO:0032319
3578	GO:0005743
3579	GO:0009261
3580	GO:0032045
3581	GO:0019319
3582	GO:0060271
3583	GO:0016071
3584	GO:0042384

3585	GO:0019866
3586	secondary_structure_distribution_H.1.0_max
3587	GO:0042420
3588	GO:0006508
3589	GO:0031058
3590	GO:0005777
3591	GO:0031056
3592	GO:0042424
3593	GO:0048541
3594	GO:0048306
3595	GO:0005900
3596	GO:0003091
3597	GO:0006085
3598	GO:0005896
3599	GO:0042579
3600	GO:0048537
3601	GO:0034313
3602	GO:0048861
3603	GO:0019955
3604	GO:0032002
3605	GO:0050891
3606	GO:0009404
3607	GO:0019614
3608	GO:0004896
3609	in_degree_median
3610	GO:0070120
3611	GO:0004897
3612	GO:0019976
3613	secondary_structure_transition_NH_mean
3614	GO:0004924
3615	GO:0016098
3616	GO:0015697
3617	GO:0009605
3618	GO:0005127
3619	in_locta_density_0.6_mean
3620	GO:0001750
3621	GO:0004911
3622	GO:0008344
3623	GO:0034754
3624	GO:0008582
3625	GO:0016624
3626	GO:0002220
3627	GO:0071339
3628	GO:0046930
3629	GO:0031301
3630	GO:0000096

3631	polarity_distribution_H.0.5_mean
3632	GO:0042733
3633	GO:0030178
3634	GO:0031253
3635	GO:0009914
3636	GO:0006171
3637	GO:0048038
3638	GO:0032642
3639	GO:0030072
3640	GO:0006140
3641	out_local_density_0.5_max
3642	GO:0016881
3643	GO:0055008
3644	GO:0046058
3645	GO:0046879
3646	GO:0016072
3647	GO:0002714
3648	GO:0051339
3649	GO:0060415
3650	GO:0005739
3651	GO:0002891
3652	GO:0002790
3653	GO:0009975
3654	GO:0006364
3655	GO:0016849
3656	GO:0002524
3657	GO:0014805
3658	GO:0015833
3659	GO:0005001
3660	GO:0042254
3661	GO:0002883
3662	GO:0030799
3663	GO:0019198
3664	GO:0051234
3665	GO:0051262
3666	GO:0002864
3667	GO:0031279
3668	GO:0001840
3669	GO:0002861
3670	GO:0004725
3671	GO:0009124
3672	GO:0001839
3673	GO:0070469
3674	GO:0002438
3675	GO:0009123
3676	GO:0043200
3677	GO:0030802

3678	GO:0042789
3679	GO:0051651
3680	GO:0030808
3681	GO:0034393
3682	GO:0030817
3683	GO:0010661
3684	in_degree_correlation_max
3685	GO:0045454
3686	GO:0009190
3687	GO:0055010
3688	GO:0046148
3689	GO:0030814
3690	GO:0010742
3691	GO:0042445
3692	GO:0009187
3693	GO:0003229
3694	GO:0001573
3695	GO:0015718
3696	GO:0014069
3697	GO:0003208
3698	GO:0030133
3699	GO:0007156
3700	GO:0090077
3701	GO:0003205
3702	GO:0015908
3703	GO:0009954
3704	GO:0003231
3705	GO:0008015
3706	GO:0003013
3707	GO:0003206
3708	GO:0070229
3709	GO:0042095
3710	GO:0006259
3711	GO:0070228
3712	GO:0005125
3713	GO:0045072
3714	GO:0006260
3715	GO:0032813
3716	GO:0042730
3717	GO:0005903
3718	GO:0035019
3719	GO:0019432
3720	GO:0005080
3721	GO:0031981
3722	GO:0008631
3723	GO:0007215
3724	GO:0042612

3725	GO:0000209
3726	GO:0030903
3727	GO:0032393
3728	GO:0048570
3729	GO:0030864
3730	GO:0048265
3731	GO:0042611
3732	GO:0002474
3733	in_local_density_0.7_max
3734	GO:0019882
3735	GO:0010887
3736	GO:0006101
3737	GO:0048753
3738	GO:0044275
3739	GO:0006582
3740	GO:0030878
3741	GO:0014848
3742	GO:0030282
3743	GO:0007585
3744	GO:0014832
3745	GO:0070011
3746	GO:0006600
3747	secondary_structure_distribution_N.0.0_mean
3748	GO:0016070
3749	GO:0022904
3750	GO:0007586
3751	GO:0006599
3752	GO:0002449
3753	GO:0008207
3754	GO:0044259
3755	GO:0009374
3756	GO:0031929
3757	GO:0070102
3758	GO:0006941
3759	GO:0044242
3760	GO:0050731
3761	GO:0030193
3762	GO:0019637
3763	GO:0009409
3764	GO:0006281
3765	GO:0006644
3766	GO:0034331
3767	GO:0045217
3768	GO:0048484
3769	GO:0016817
3770	GO:0042491
3771	GO:0031256

3772	GO:0002446
3773	GO:0014820
3774	GO:0014824
3775	GO:0046364
3776	GO:0005740
3777	GO:0045879
3778	GO:0046165
3779	GO:0046880
3780	GO:0045087
3781	GO:0031649
3782	GO:0005720
3783	GO:0001880
3784	GO:0032276
3785	GO:0031966
3786	GO:0016327
3787	GO:0042036
3788	GO:0046882
3789	out_degree_median
3790	GO:0016338
3791	GO:0032924
3792	GO:0005504
3793	GO:0032277
3794	GO:0042165
3795	GO:0043296
3796	GO:0048185
3797	GO:0005923
3798	GO:0030510
3799	GO:0070160
3800	GO:0008374
3801	GO:0048066
3802	GO:0007223
3803	GO:0033540
3804	GO:0016055
3805	GO:0007512
3806	GO:0033631
3807	GO:0043120
3808	GO:0046521
3809	GO:0019841
3810	GO:0051926
3811	GO:0006953
3812	GO:0060042
3813	GO:0000776
3814	GO:0044438
3815	GO:0030140
3816	GO:0010906
3817	GO:0042271
3818	GO:0044439

3819	GO:0015291
3820	GO:0030201
3821	GO:0033280
3822	GO:0006109
3823	GO:0005244
3824	GO:0043129
3825	GO:0022832
3826	GO:0001505
3827	GO:0015931
3828	GO:0010675
3829	GO:0019321
3830	GO:0048875
3831	GO:0042364
3832	GO:0016780
3833	GO:0048562
3834	GO:0016646
3835	GO:0016877
3836	GO:0005770
3837	GO:0006027
3838	GO:0005681
3839	GO:0030176
3840	GO:0051351
3841	GO:0004622
3842	GO:0018130
3843	GO:0006752
3844	GO:0016857
3845	GO:0051352
3846	GO:0006026
3847	GO:0031397
3848	GO:0009062
3849	GO:0009113
3850	GO:0016411
3851	GO:0031145
3852	GO:0004091
3853	GO:0051437
3854	GO:0043498
3855	out_degree_max
3856	GO:0032279
3857	GO:0030433
3858	GO:0034702
3859	GO:0006635
3860	GO:0051436
3861	GO:0009130
3862	GO:0051444
3863	GO:0007605
3864	GO:0042288
3865	GO:0051439

3866	GO:0006534
3867	GO:0046398
3868	GO:0009129
3869	GO:0051443
3870	GO:0003678
3871	GO:0030594
3872	hydrophobicity_distribution_N.0.25_mean
3873	GO:0004981
3874	GO:0010744
3875	GO:0006547
3876	GO:0015464
3877	GO:0046835
3878	GO:0031952
3879	GO:0016907
3880	GO:0051213
3881	GO:0006548
3882	GO:0007207
3883	GO:0031968
3884	in_degree_variance
3885	GO:0032259
3886	GO:0016763
3887	GO:0009077
3888	GO:0031398
3889	GO:0016701
3890	GO:0016832
3891	GO:0019867
3892	GO:0016884
3893	GO:0006479
3894	GO:0016861
3895	GO:0009311
3896	GO:0051380
3897	GO:0009075
3898	GO:0004952
3899	GO:0016702
3900	GO:0006954
3901	GO:0043648
3902	GO:0003321
3903	GO:0043414
3904	GO:0019239
3905	GO:0051378
3906	GO:0017040
3907	GO:0009119
3908	GO:0048665
3909	GO:0004935
3910	GO:0008213
3911	GO:0030432
3912	GO:0009156

3913	GO:0021520
3914	GO:0009071
3915	GO:0004993
3916	GO:0021521
3917	GO:0006106
3918	GO:0008227
3919	GO:0006047
3920	GO:0060420
3921	GO:0042033
3922	GO:0009163
3923	GO:0004939
3924	GO:0021514
3925	GO:0030672
3926	GO:0042153
3927	GO:0055024
3928	GO:0014821
3929	GO:0008211
3930	GO:0060579
3931	GO:0017085
3932	GO:0046479
3933	GO:0060043
3934	GO:0030819
3935	GO:0042455
3936	GO:0045073
3937	GO:0051954
3938	GO:0007224
3939	GO:0055021
3940	GO:0001993
3941	GO:0034394
3942	GO:0046129
3943	GO:0001976
3944	GO:0019852
3945	GO:0050755
3946	GO:0030816
3947	GO:0043413
3948	GO:0042451
3949	GO:0009220
3950	GO:0070085
3951	GO:0045080
3952	GO:0005496
3953	GO:0016868
3954	GO:0007218
3955	GO:0008308
3956	GO:0031225
3957	GO:0046125
3958	GO:0009101
3959	GO:0046457

3960	GO:0004614
3961	GO:0034404
3962	GO:0006486
3963	GO:0034654
3964	GO:0001516
3965	GO:0048531
3966	GO:0006740
3967	GO:0006663
3968	GO:0034446
3969	GO:0016405
3970	GO:0070646
3971	VanDerWaal_distribution_P.0.75_mean
3972	GO:0016878
3973	GO:0022613
3974	GO:0046469
3975	GO:0006516
3976	GO:0046461
3977	GO:0042987
3978	GO:0030258
3979	GO:0015732
3980	GO:0045088
3981	GO:0009898
3982	GO:0005588
3983	GO:0019433
3984	GO:0005583
3985	GO:0002020
3986	GO:0032892
3987	GO:0046464
3988	GO:0007168
3989	GO:0032303
3990	GO:0044269
3991	GO:0070325
3992	out_local_density_0.6_max
3993	GO:0043518
3994	GO:0030553
3995	GO:0050750
3996	GO:0045254
3997	GO:0032306
3998	GO:0042246
3999	GO:0048013
4000	GO:0006086
4001	GO:0006776
4002	GO:0008234
4003	GO:0032925
4004	GO:0032308
4005	GO:0007628
4006	GO:0003708

4007	GO:0002281
4008	secondary_structure_distribution_P.1.0_mean
4009	GO:0032310
4010	GO:0022838
4011	GO:0070988
4012	GO:0052033
4013	GO:0022803
4014	GO:0052169
4015	GO:0009822
4016	GO:0045342
4017	GO:0032305
4018	GO:0002282
4019	GO:0015267
4020	GO:0070989
4021	GO:0052167
4022	GO:0045346
4023	GO:0005216
4024	GO:0030512
4025	GO:0052257
4026	GO:0004623
4027	GO:0007205
4028	GO:0052255
4029	GO:0052166
4030	GO:0047498
4031	GO:0007589
4032	GO:0001774
4033	GO:0006986
4034	GO:0052555
4035	GO:0052509
4036	GO:0052553
4037	GO:0052552
4038	GO:0052510
4039	GO:0052031
4040	GO:0045341
4041	GO:0005267
4042	GO:0052556
4043	GO:0045343
4044	GO:0016812
4045	GO:0016885
4046	GO:0005164
4047	GO:0052305
4048	GO:0031670
4049	GO:0004385
4050	GO:0052308
4051	GO:0033993
4052	GO:0052306
4053	GO:0032757

4054	GO:0043403
4055	GO:0001875
4056	GO:0045089
4057	GO:0055080
4058	GO:0046849
4059	GO:0006091
4060	GO:0002054
4061	GO:0046504
4062	GO:0034440
4063	GO:0019203
4064	GO:0046460
4065	GO:0016634
4066	GO:0046463
4067	GO:0042220
4068	GO:0002029
4069	GO:0019395
4070	GO:0015631
4071	GO:0006705
4072	GO:0015807
4073	GO:0022401
4074	GO:0042752
4075	GO:0008212
4076	GO:0014073
4077	GO:0032342
4078	GO:0023058
4079	GO:0019861
4080	GO:0032341
4081	GO:0002755
4082	GO:0034121
4083	GO:0004704
4084	GO:0031967
4085	GO:0006105
4086	GO:0034105
4087	GO:0000299
4088	GO:0016607
4089	GO:0031975
4090	GO:0004867
4091	GO:0045851
4092	GO:0006289
4093	GO:0019320
4094	GO:0000287
4095	GO:0051452
4096	GO:0046365
4097	out_locta_density_0.5_mean
4098	GO:0032934
4099	GO:0006096
4100	GO:0046164

4101	GO:0021549
4102	GO:0031965
4103	GO:0060137
4104	GO:0002027
4105	GO:0015297
4106	GO:0015300
4107	GO:0051001
4108	GO:0010243
4109	GO:0005793
4110	GO:0014866
4111	GO:0046912
4112	polarizability_distribution_H.0.25_mean
4113	GO:0048770
4114	GO:0048487
4115	GO:0050811
4116	GO:0006812
4117	GO:0006811
4118	GO:0042470
4119	GO:0000502
4120	GO:0032637
4121	VanDerWaal_distribution_H.0.25_mean
4122	GO:0044437
4123	GO:0016020
4124	GO:0000151
4125	GO:0002444
4126	GO:0031461
4127	GO:0031396
4128	GO:0002791
4129	GO:0021795
4130	GO:0019953
4131	GO:0090276
4132	GO:0019370
4133	GO:0043450
4134	GO:0090087
4135	GO:0033596
4136	GO:0032602
4137	GO:0050796
4138	GO:0004075
4139	GO:0005606
4140	GO:0005604
4141	GO:0006584
4142	GO:0030136
4143	GO:0005581
4144	GO:0050885
4145	in_degree_correlation_mean
4146	GO:0005881
4147	GO:0005605

4148	GO:0018958
4149	GO:0051233
4150	GO:0042461
4151	GO:0030198
4152	GO:0030935
4153	GO:0043121
4154	GO:0043256
4155	GO:0009712
4156	GO:0044420
4157	GO:0046942
4158	GO:0042310
4159	GO:0005587
4160	topological_change_0.3_0.4
4161	GO:0034311
4162	GO:0021510
4163	out_degree_variance
4164	GO:0070013
4165	GO:0015849
4166	GO:0031224
4167	GO:0006457
4168	GO:0016021
4169	GO:0019217
4170	GO:0005654
4171	GO:0030299
4172	GO:0016874
4173	GO:0020027
4174	GO:0010565
4175	GO:0051919
4176	GO:0008375
4177	GO:0008217
4178	GO:0016421
4179	GO:0046339
4180	GO:0002504
4181	GO:0032395
4182	GO:0042613
4183	GO:0032606
4184	GO:0043227
4185	GO:0033089
4186	GO:0030246
4187	GO:0008035
4188	GO:0043231
4189	GO:0030173
4190	GO:0031113
4191	GO:0031228
4192	GO:0031112
4193	secondary_structure_distribution_H.0.75_mean
4194	GO:0031116

4195	GO:0046785
4196	GO:0007091
4197	GO:0005625
4198	GO:0007029
4199	GO:0050873
4200	GO:0043178
4201	GO:0016775
4202	GO:0006820
4203	GO:0019835
4204	GO:0015695
4205	GO:0033691
4206	GO:0007413
4207	GO:0032982
4208	GO:0008091
4209	GO:0030660
4210	in_local_density_0.4_max
4211	GO:0055117
4212	GO:0008081
4213	GO:0055066
4214	GO:0044248
4215	GO:0003987
4216	GO:0000038
4217	GO:0031282
4218	GO:0016291
4219	GO:0003854
4220	GO:0042743
4221	GO:0010008
4222	GO:0006695
4223	GO:0008135
4224	GO:0004117
4225	GO:0003743
4226	GO:0033865
4227	GO:0044440
4228	GO:0043621
4229	GO:0007602
4230	GO:0014049
4231	GO:0001783
4232	GO:0043088
4233	GO:0009583
4234	topological_change_0.7_0.8
4235	GO:0006023
4236	GO:0032489
4237	GO:0042354
4238	GO:0019201
4239	GO:0046653
4240	GO:0006024
4241	GO:0006004

4242	GO:0007339
4243	GO:0032225
4244	GO:0031227
4245	GO:0042541
4246	GO:0006760
4247	GO:0055088
4248	GO:0009988
4249	GO:0016748
4250	GO:0070569
4251	GO:0050897
4252	GO:0035036
4253	polarity_distribution_N.1.0_max
4254	GO:0019238
4255	GO:0006011
4256	GO:0016209
4257	GO:0006555
4258	GO:0001101
4259	GO:0010578
4260	GO:0016782
4261	GO:0046476
4262	GO:0042278
4263	GO:0046474
4264	GO:0009173
4265	GO:0045762
4266	GO:0004689
4267	GO:0046128
4268	GO:0005964
4269	GO:0046477
4270	GO:0016416
4271	GO:0046049
4272	GO:0007189
4273	GO:0016406
4274	GO:0006544
4275	GO:0005984
4276	GO:0006222
4277	GO:0004095
4278	GO:0009312
4279	GO:0004177
4280	GO:0008195
4281	GO:0007190
4282	GO:0006206
4283	GO:0009070
4284	GO:0042392
4285	GO:0016841
4286	GO:0006207
4287	GO:0010579
4288	GO:0016801

4289	GO:0005741
4290	GO:0050667
4291	GO:0019856
4292	GO:0016645
4293	GO:0015980
4294	GO:0009174
4295	GO:0009060
4296	GO:0008203
4297	GO:0003836
4298	GO:0006533
4299	GO:0006084
4300	GO:0006098
4301	GO:0016125
4302	GO:0008373
4303	GO:0046487
4304	GO:0006531
4305	GO:0009264
4306	GO:0004396
4307	GO:0046033
4308	GO:0004872
4309	GO:0008499
4310	GO:0009185
4311	GO:0031645
4312	GO:0006112
4313	GO:0016833
4314	GO:0005977
4315	GO:0046349
4316	GO:0006412
4317	GO:0051970
4318	in_degree_max
4319	GO:0002064
4320	GO:0042572
4321	GO:0015036
4322	GO:0009219
4323	GO:0042393
4324	GO:0050805
4325	in_locta_density_0.5_mean
4326	out_degree_correlation_mean
4327	GO:0009394
4328	GO:0005815
4329	GO:0009448
4330	GO:0002675
4331	GO:0009161
4332	GO:0044446
4333	GO:0007618
4334	GO:0051117
4335	GO:0009218

4336	GO:0005774
4337	GO:0000323
4338	GO:0050857
4339	GO:0051928
4340	GO:0017111
4341	GO:0005764
4342	GO:0051605
4343	GO:0016818
4344	GO:0046486
4345	GO:0030279
4346	GO:0051604
4347	GO:0032508
4348	GO:0051340
4349	GO:0043462
4350	GO:0015850
4351	GO:0016462
4352	GO:0016485
4353	GO:0070979
4354	GO:0006270
4355	GO:0000777
4356	GO:0006268
4357	GO:0000808
4358	GO:0007062
4359	GO:0000152
4360	GO:0042287
4361	GO:0005680
4362	GO:0005664
4363	GO:0043531
4364	GO:0003688
4365	GO:0032392
4366	GO:0051438
4367	GO:0048029
4368	GO:0051322
4369	GO:0008236
4370	GO:0000090
4371	GO:0004252
4372	GO:0033555
4373	GO:0005536
4374	GO:0019992
4375	GO:0017171
4376	GO:0016879
4377	GO:0019200
4378	GO:0022627
4379	GO:0022626
4380	GO:0046470
4381	GO:0015935
4382	GO:0006195

4383	GO:0033279
4384	GO:0005840
4385	GO:0006007
4386	GO:0052564
4387	GO:0005778
4388	GO:0052572
4389	GO:0042416
4390	GO:0051084
4391	GO:0005615
4392	GO:0005507
4393	GO:0031903
4394	GO:0006458
4395	GO:0051085
4396	GO:0007595
4397	GO:0006700
4398	GO:0008038
4399	GO:0045329
4400	AA_composition_K_mean
4401	GO:0032561
4402	GO:0051181
4403	GO:0019001
4404	GO:0051019
4405	GO:0016776
4406	GO:0008432
4407	GO:0043449
4408	GO:0001967
4409	in_in_topological_mean
4410	GO:0006691
4411	GO:0004467
4412	GO:0015645
4413	GO:0032769
4414	GO:0010872
4415	GO:0010873
4416	GO:0015698
4417	GO:0005031
4418	GO:0045907
4419	GO:0005035
4420	GO:0016728
4421	GO:0035005
4422	GO:0004748
4423	GO:0019205
4424	GO:0004726
4425	GO:0009263
4426	GO:0004953
4427	GO:0045665
4428	GO:0009262
4429	GO:0030166

4430	GO:0004954
4431	GO:0031404
4432	GO:0002903
4433	GO:0004955
4434	GO:0009132
4435	GO:0002902
4436	GO:0003682
4437	GO:0006107
4438	GO:0006650
4439	GO:0009247
4440	GO:0045744
4441	GO:0060405
4442	GO:0044238
4443	GO:0043620
4444	GO:0033057
4445	out_degree_correlation_variance
4446	GO:0008654
4447	GO:0016308
4448	GO:0016309
4449	GO:0045909
4450	GO:0006396
4451	GO:0009395
4452	GO:0016307
4453	GO:0008233
4454	GO:0045347
4455	GO:0005254
4456	GO:0006677
4457	GO:0005217
4458	GO:0021602
4459	GO:0042359
4460	GO:0021631
4461	GO:0021554
4462	GO:0005044
4463	GO:0030247
4464	GO:0050892
4465	GO:0043233
4466	GO:0050746
4467	GO:0006914
4468	GO:0001871
4469	GO:0017162
4470	GO:0006111
4471	GO:0035235
4472	GO:0034470
4473	GO:0006884
4474	GO:0045354
4475	VanDerWaal_distribution_H.0.75_mean
4476	GO:0032607

4477	GO:0050872
4478	GO:0045078
4479	GO:0070372
4480	GO:0005126
4481	GO:0045349
4482	GO:0032647
4483	GO:0070371
4484	GO:0033235
4485	GO:0001702
4486	GO:0033233
4487	GO:0046873
4488	GO:0043274
4489	GO:0019789
4490	GO:0005262
4491	GO:0070374
4492	GO:0016925
4493	polarizability_distribution_H.0.75_mean
4494	GO:0007567
4495	GO:0005261
4496	GO:0015270
4497	GO:0014044
4498	GO:0019724
4499	GO:0006325
4500	GO:0016064
4501	GO:0016568
4502	GO:0044428
4503	in_local_density_0.3_max
4504	GO:0003018
4505	GO:0031579
4506	GO:0006958
4507	GO:0006639
4508	GO:0008094
4509	GO:0006959
4510	GO:0016199
4511	GO:0002541
4512	GO:0006638
4513	GO:0016198
4514	GO:0004866
4515	GO:0006956
4516	GO:0033829
4517	GO:0006662
4518	GO:0033628
4519	GO:0005112
4520	GO:0002526
4521	GO:0007386
4522	GO:0007219
4523	GO:0002455

4524	GO:0048566
4525	GO:0006957
4526	GO:0030414
4527	GO:0005790
4528	GO:0042813
4529	GO:0006200
4530	GO:0046473
4531	GO:0005758
4532	GO:0007164
4533	GO:0060228
4534	GO:0005876
4535	GO:0001738
4536	GO:0033700
4537	GO:0001736
4538	GO:0034380
4539	GO:0032488
4540	GO:0004926
4541	GO:0015914
4542	GO:0046887
4543	GO:0017127
4544	GO:0004718
4545	GO:0031513
4546	GO:0042166
4547	GO:0002031
4548	GO:0007418
4549	GO:0021938
4550	GO:0050916
4551	GO:0021937
4552	GO:0016799
4553	GO:0050917
4554	GO:0009146
4555	GO:0021910
4556	GO:0050909
4557	GO:0003073
4558	GO:0008527
4559	GO:0005113
4560	GO:0044264
4561	GO:0042742
4562	GO:0044042
4563	GO:0060292
4564	GO:0008048
4565	GO:0007588
4566	GO:0010851
4567	GO:0002087
4568	GO:0008330
4569	GO:0006073
4570	GO:0010853

4571	GO:0044065
4572	GO:0043576
4573	GO:0030250
4574	GO:0030249
4575	GO:0000421
4576	GO:0004886
4577	GO:0022624
4578	GO:0018205
4579	GO:0008538
4580	GO:0010860
4581	hydrophobicity_distribution_P.0.75_mean
4582	GO:0008537
4583	GO:0016602
4584	GO:0005776
4585	GO:0045136
4586	GO:0001669
4587	GO:0005976
4588	GO:0035240
4589	secondary_structure_distribution_N.0.25_mean
4590	GO:0014051
4591	GO:0050432
4592	GO:0006014
4593	GO:0009110
4594	GO:0015812
4595	GO:0051937
4596	GO:0005640
4597	GO:0008209
4598	GO:0016051
4599	GO:0008331
4600	GO:0050433
4601	GO:0018904
4602	GO:0005042
4603	in_degree_correlation_variance
4604	GO:0090181
4605	GO:0001660
4606	GO:0002042
4607	GO:0045540
4608	GO:0009072
4609	GO:0016570
4610	GO:0016740
4611	GO:0016569
4612	GO:0004972
4613	GO:0007623
4614	GO:0000228
4615	GO:0017146
4616	GO:0016615
4617	GO:0016859

4618	GO:0043176
4619	GO:0044454
4620	GO:0006108
4621	GO:0045019
4622	GO:0004659
4623	GO:0016713
4624	GO:0006333
4625	GO:0004311
4626	GO:0043954
4627	GO:0045017
4628	GO:0046174
4629	GO:0015936
4630	GO:0034599
4631	GO:0042745
4632	GO:0030145
4633	GO:0008199
4634	GO:0031974
4635	GO:0048512
4636	GO:0006826
4637	GO:0016722
4638	GO:0006012
4639	GO:0000041
4640	GO:0016093
4641	GO:0016090
4642	GO:0004875
4643	GO:0009186
4644	GO:0005579
4645	hydrophobicity_distribution_P.1.0_max
4646	GO:0001867
4647	GO:0043043
4648	GO:0009200
4649	GO:0007598
4650	GO:0009396
4651	GO:0001848
4652	GO:0016778
4653	GO:0006750
4654	GO:0005746
4655	GO:0003840
4656	GO:0046502
4657	VanDerWaal_distribution_P.1.0_max
4658	GO:0016755
4659	VanDerWaal_distribution_P.1.0_mean
4660	GO:0005245
4661	GO:0006006
4662	GO:0008332
4663	GO:0007625
4664	GO:0008080

4665	GO:0006551
4666	GO:0008171
4667	GO:0005891
4668	GO:0009083
4669	GO:0034704
4670	GO:0001892
4671	GO:0022843
4672	GO:0021988
4673	GO:0034703
4674	GO:0021891
4675	GO:0010889
4676	GO:0005003
4677	GO:0042116
4678	GO:0005005
4679	GO:0031403
4680	GO:0030259
4681	GO:0055085
4682	GO:0021889
4683	GO:0021772
4684	GO:0031290
4685	GO:0006537
4686	GO:0046875
4687	GO:0042069
4688	GO:0008046
4689	GO:0009064
4690	GO:0046356
4691	GO:0048843
4692	GO:0070471
4693	GO:0045499
4694	GO:0042923
4695	GO:0046688
4696	GO:0048846
4697	GO:0070472
4698	GO:0009084
4699	GO:0051187
4700	GO:0006563
4701	GO:0033539
4702	GO:0004995
4703	GO:0048841
4704	GO:0042053
4705	GO:0030517
4706	GO:0043010
4707	GO:0070474
4708	GO:0006565
4709	GO:0009109
4710	GO:0017154
4711	GO:0033017

4712	GO:0046467
4713	GO:0005388
4714	GO:0005542
4715	GO:0001992
4716	GO:0006099
4717	GO:0042355
4718	GO:0003078
4719	GO:0008088
4720	GO:0005000
4721	GO:0030148
4722	GO:0004974
4723	GO:0008417
4724	GO:0015085
4725	GO:0008188
4726	GO:0046519
4727	GO:0004937
4728	GO:0019317
4729	GO:0007217
4730	GO:0046100
4731	GO:0004936
4732	GO:0006672
4733	GO:0030890
4734	GO:0009086
4735	GO:0006261
4736	GO:0001614
4737	GO:0016502
4738	GO:0015929
4739	GO:0004969
4740	GO:0000731
4741	GO:0006687
4742	GO:0016591
4743	GO:0046950
4744	GO:0006664
4745	GO:0006103
4746	GO:0043596
4747	GO:0004047
4748	GO:0006643
4749	GO:0004143
4750	GO:0030894
4751	GO:0009203
4752	GO:0006546
4753	GO:0043020
4754	GO:0006665
4755	GO:0016175
4756	GO:0016714
4757	GO:0005658
4758	GO:0009207

4759	GO:0043601
4760	GO:0045333
4761	GO:0004332
4762	GO:0006144
4763	GO:0009127
4764	GO:0030062
4765	GO:0005765
4766	GO:0017169
4767	GO:0009168
4768	GO:0005635
4769	GO:0045239
4770	GO:0019695
4771	GO:0009167
4772	GO:0009126
4773	GO:0004364
4774	GO:0000779
4775	GO:0016765
4776	GO:0060192
4777	GO:0070508
4778	GO:0090208
4779	GO:0035376
4780	GO:0035382
4781	GO:0070653
4782	GO:0090207
4783	GO:0015245
4784	GO:0010903
4785	GO:0034366
4786	GO:0046027
4787	GO:0042904
4788	GO:0010901
4789	GO:0030135
4790	GO:0010896
4791	GO:0019864
4792	GO:0003841
4793	GO:0035238
4794	GO:0016803
4795	GO:0042905
4796	GO:0005529
4797	GO:0004739
4798	GO:0004738
4799	GO:0031503
4800	GO:0034383
4801	GO:0030641
4802	GO:0006163
4803	GO:0008277
4804	GO:0051453
4805	GO:0031970

4806	GO:0006164
4807	GO:0022600
4808	GO:0008380
4809	GO:0051028
4810	GO:0009191
4811	GO:0050830
4812	GO:0009134
4813	GO:0005104
4814	GO:0008543
4815	GO:0005111
4816	GO:0046503
4817	GO:0004383
4818	GO:0045350
4819	GO:0034123
4820	GO:0046068
4821	GO:0045357
4822	GO:0045359
4823	GO:0034614
4824	GO:0006182
4825	GO:0006029
4826	GO:0009259
4827	GO:0008253
4828	GO:0009260
4829	GO:0008252
4830	GO:0009265
4831	GO:0006775
4832	GO:0009133
4833	GO:0006244
4834	GO:0043587
4835	GO:0046085
4836	GO:0050906
4837	GO:0006221
4838	GO:0006220
4839	GO:0042744
4840	GO:0043094
4841	GO:0006213
4842	GO:0070301
4843	GO:0046131
4844	GO:0019136
4845	GO:0009268
4846	GO:0005798
4847	GO:0019206
4848	GO:0043266
4849	GO:0009744
4850	GO:0009250
4851	GO:0005978
4852	GO:0045745

4853	GO:0034285
4854	GO:0001990
4855	GO:0002793
4856	GO:0090277
4857	GO:0033189
4858	GO:0009593
4859	in_in_topological_variance
4860	GO:0031953
4861	GO:0000036
4862	GO:0010885
4863	GO:0046459
4864	GO:0016594
4865	GO:0022900
4866	GO:0006839
4867	GO:0031402
4868	GO:0046483
4869	GO:0040020
4870	out_out_topological_variance
4871	GO:0051445
4872	GO:0016529
4873	GO:0046527
4874	GO:0035251
4875	GO:0007622
4876	GO:0016528
4877	GO:0033549
4878	GO:0005078
4879	GO:0008238
4880	GO:0017017
4881	GO:0009743
4882	GO:0009251
4883	GO:0048857
4884	GO:0006869
4885	GO:0044247
4886	GO:0071514
4887	GO:0007274
4888	GO:0006349
4889	GO:0006570
4890	GO:0006821
4891	GO:0042597
4892	GO:0032024
4893	GO:0030288
4894	GO:0000272
4895	GO:0019359
4896	GO:0006694
4897	GO:0070542
4898	GO:0042379
4899	GO:0001664

4900	GO:0019956
4901	GO:0019957
4902	GO:0001637
4903	GO:0008009
4904	GO:0016493
4905	GO:0051024
4906	GO:0001961
4907	GO:0004950
4908	GO:0010460
4909	VanDerWaal_distribution_N.0.5_mean
4910	GO:0006000
4911	GO:0006002
4912	GO:0034379
4913	GO:0019693
4914	GO:0008034
4915	GO:0009566
4916	GO:0031018
4917	GO:0005945
4918	GO:0070061
4919	GO:0050953
4920	GO:0070095
4921	GO:0004470
4922	GO:0034451
4923	GO:0007601
4924	GO:0006692
4925	GO:0004331
4926	GO:0006693
4927	GO:0008443
4928	GO:0050665
4929	GO:0003872
4930	GO:0042312
4931	GO:0030388
4932	GO:0048384
4933	GO:0006656
4934	GO:0021536
4935	GO:0015485
4936	GO:0046039
4937	GO:0030204
4938	GO:0009125
4939	GO:0055086
4940	GO:0070567
4941	GO:0003044
4942	GO:0051276
4943	GO:0009165
4944	GO:0046520
4945	GO:0045104
4946	GO:0035150

4947	GO:0060052
4948	GO:0045103
4949	GO:0005791
4950	GO:0044425
4951	GO:0045109
4952	GO:0005883
4953	GO:0050880
4954	GO:0060053
4955	GO:0033762
4956	GO:0045110
4957	GO:0005875
4958	GO:0051766
4959	GO:0046513
4960	GO:0034593
4961	GO:0008440
4962	GO:0031405
4963	GO:0034595
4964	GO:0046030
4965	GO:0004437
4966	GO:0004445
4967	polarity_distribution_N.1.0_mean
4968	GO:0010817
4969	GO:0006020
4970	GO:0060024
4971	GO:0042605
4972	GO:0004439
4973	GO:0006414
4974	GO:0005643
4975	GO:0030057
4976	GO:0055093
4977	GO:0015248
4978	GO:0048821
4979	GO:0046500
4980	GO:0034637
4981	GO:0006654
4982	GO:0034235
4983	GO:0008021
4984	GO:0008172
4985	GO:0000002
4986	GO:0006688
4987	GO:0006772
4988	GO:0042723
4989	GO:0048515
4990	GO:0005775
4991	GO:0004806
4992	GO:0042559
4993	GO:0043202

4994	GO:0046146
4995	GO:0047372
4996	GO:0045777
4997	GO:0034185
4998	GO:0003723
4999	GO:0019369
5000	GO:0007286
5001	GO:0003071
5002	GO:0046498
5003	GO:0044237
5004	GO:0003084
5005	GO:0008614
5006	GO:0050651
5007	GO:0006013
5008	GO:0016126
5009	GO:0008615
5010	GO:0050655
5011	GO:0004769
5012	GO:0042816
5013	GO:0030206
5014	GO:0019374
5015	GO:0042819
5016	GO:0006681
5017	in_local_density_0.1_max
5018	GO:0019213
5019	GO:0033765
5020	GO:0008376
5021	in_local_density_0_max
5022	GO:0006702
5023	in_local_density_0.2_max
5024	GO:0016717
5025	hydrophobicity_distribution_N.1.0_max
5026	GO:0006493
5027	GO:0004060
5028	GO:0034416
5029	GO:0015014
5030	GO:0019405
5031	GO:0042157
5032	secondary_structure_distribution_N.1.0_max
5033	GO:0016887
5034	GO:0006636
5035	GO:0004065
5036	GO:0006403
5037	GO:0042623
5038	GO:0042711
5039	GO:0008484
5040	GO:0045814

5041	GO:0060746
5042	GO:0046456
5043	GO:0050654
5044	GO:0050658
5045	GO:0018211
5046	GO:0019363
5047	GO:0006342
5048	GO:0018406
5049	GO:0033014
5050	GO:0006690
5051	GO:0009435
5052	GO:0051236
5053	GO:0008757
5054	GO:0018103
5055	GO:0046874
5056	GO:0006779
5057	GO:0004774
5058	GO:0018317
5059	GO:0019441
5060	GO:0033559
5061	GO:0050657
5062	GO:0006783
5063	GO:0015662
5064	GO:0004775
5065	GO:0019627
5066	GO:0003831
5067	GO:0046218
5068	GO:0042158
5069	GO:0006703
5070	GO:0006104
5071	GO:0000050
5072	GO:0002686
5073	GO:0042430
5074	GO:0004461
5075	GO:0046489
5076	GO:0043604
5077	GO:0006569
5078	GO:0003945
5079	GO:0000030
5080	GO:0015874
5081	GO:0006586
5082	GO:0019322
5083	GO:0014061
5084	GO:0035268
5085	GO:0042436
5086	GO:0048243
5087	GO:0004582

5088	GO:0042434
5089	GO:0035269
5090	GO:0006568
5091	GO:0031501
5092	GO:0006505
5093	GO:0004305
5094	GO:0034645
5095	GO:0004103
5096	GO:0006497
5097	GO:0006506
5098	GO:0031418
5099	GO:0019751
5100	GO:0006753
5101	GO:0016595
5102	GO:0009059
5103	GO:0042396
5104	GO:0006521
5105	GO:0004090
5106	GO:0009117
5107	GO:0050327
5108	GO:0006646
5109	GO:0004301
5110	GO:0050997
5111	GO:0004849
5112	GO:0047115
5113	GO:0015721
5114	GO:0006580
5115	GO:0048148
5116	GO:0004558
5117	GO:0046337
5118	GO:0004457
5119	GO:0004459
5120	GO:0046335
5121	GO:0042428
5122	GO:0006072
5123	GO:0006167
5124	GO:0008237
5125	GO:0016255
5126	GO:0045721
5127	GO:0034341
5128	GO:0015075
5129	GO:0006352
5130	GO:0006367
5131	GO:0006188
5132	GO:0008401
5133	GO:0006189
5134	GO:0003995

5135	GO:0046040
5136	GO:0006172
5137	GO:0016165
5138	GO:0009150
5139	out_local_density_0.7_max
5140	GO:0006177
5141	GO:0009180
5142	GO:0004312
5143	GO:0046037
5144	GO:0009135
5145	GO:0006166
5146	GO:0008270
5147	GO:0009141
5148	GO:0043174
5149	GO:0009136
5150	GO:0043101
5151	GO:0046031
5152	GO:0009152
5153	GO:0050664
5154	GO:0009179
5155	GO:0006704
5156	GO:0009188
5157	GO:0016234
5158	GO:0042438
5159	GO:0033162
5160	GO:0030384
5161	GO:0006527
5162	GO:0045009
5163	GO:0021952
5164	GO:0048593
5165	GO:0008144
5166	GO:0040029
5167	GO:0032608
5168	GO:0004339
5169	GO:0032648
5170	GO:0034369
5171	GO:0015926
5172	GO:0032479
5173	GO:0032481
5174	GO:0004768
5175	GO:0070328
5176	GO:0034367
5177	GO:0045980
5178	GO:0019098
5179	GO:0034368
5180	GO:0008184
5181	GO:0034381

5182	GO:0021983
5183	GO:0004645
5184	GO:0007617
5185	GO:0003997
5186	GO:0043691
5187	GO:0045717
5188	GO:0005657
5189	GO:0006659
5190	GO:0006658
5191	GO:0045187
5192	GO:0042749
5193	GO:0022410
5194	GO:0050802
5195	GO:0002636
5196	GO:0002634
5197	GO:0008354
5198	GO:0006183
5199	GO:0046051
5200	GO:0006297
5201	GO:0006241
5202	GO:0043619
5203	GO:0006228
5204	GO:0043618
5205	GO:0034062
5206	GO:0032728
5207	GO:0004017
5208	GO:0032993
5209	GO:0003887
5210	GO:0003896
5211	in_clustering_variance
5212	GO:0034061
5213	GO:0003899
5214	GO:0009214
5215	GO:0004550
5216	GO:0004115
5217	GO:0017110
5218	GO:0009208
5219	GO:0001963
5220	GO:0009148
5221	GO:0009147
5222	GO:0043168
5223	GO:0006360
5224	GO:0017158
5225	GO:0033483
5226	GO:0016779
5227	GO:0016791
5228	GO:0000428

5229	GO:0051705
5230	GO:0055029
5231	GO:0030880
5232	GO:0006813
5233	GO:0006354
5234	GO:0010878
5235	GO:0006368
5236	GO:0042423
5237	GO:0032549
5238	GO:0005736
5239	GO:0016410
5240	GO:0046036
5241	GO:0009209
5242	GO:0006383
5243	GO:0005665
5244	GO:0005666
5245	GO:0055092
5246	GO:0090101
5247	GO:0042632
5248	GO:0044306
5249	GO:0007140
5250	GO:0043679
5251	GO:0032781
5252	GO:0042982
5253	GO:0030534
5254	GO:0043206
5255	GO:0009750
5256	GO:0030867
5257	polarity_distribution_H.1.0_max
5258	GO:0009081
5259	GO:0004630
5260	GO:0010181
5261	GO:0016706
5262	GO:0030823
5263	GO:0030826
5264	GO:0009791
5265	GO:0051606
5266	GO:0022891
5267	GO:0010522
5268	GO:0008324
5269	GO:0042301
5270	GO:0050650
5271	GO:0032364
5272	GO:0006807
5273	GO:0010876
5274	GO:0002063
5275	GO:0006801

5276	GO:0007271
5277	GO:0042554
5278	GO:0070874
5279	GO:0006397
5280	GO:0006591
5281	GO:0009111
5282	GO:0042363
5283	GO:0006434
5284	GO:0042627
5285	GO:0004828
5286	GO:0034361
5287	GO:0007422
5288	GO:0034382
5289	GO:0005319
5290	GO:0005882
5291	GO:0034385
5292	GO:0045111
5293	GO:0034384
5294	GO:0006800
5295	GO:0031543
5296	GO:0001972
5297	GO:0010288
5298	GO:0005788
5299	GO:0042415
5300	GO:0006139
5301	GO:0045912
5302	GO:0005813
5303	GO:0010677
5304	GO:0016802
5305	GO:0004013
5306	GO:0004653
5307	GO:0003829
5308	GO:0018242
5309	GO:0018243
5310	GO:0008109
5311	GO:0003993
5312	GO:0017060
5313	GO:0004594
5314	GO:0015937
5315	GO:0034481
5316	GO:0047756
5317	GO:0004062
5318	GO:0008146
5319	GO:0004415
5320	GO:0015018
5321	GO:0070568
5322	GO:0006596

5323	GO:0050510
5324	GO:0009226
5325	GO:0045426
5326	GO:0070035
5327	GO:0000309
5328	GO:0006595
5329	GO:0047238
5330	GO:0003873
5331	GO:0042765
5332	GO:0006743
5333	GO:0008186
5334	GO:0004515
5335	GO:0008216
5336	GO:0006432
5337	GO:0006003
5338	GO:0017176
5339	GO:0005980
5340	GO:0016997
5341	GO:0006744
5342	GO:0050508
5343	GO:0004386
5344	GO:0008295
5345	GO:0004826
5346	GO:0003923
5347	GO:0006684
5348	GO:0043237
5349	GO:0004004
5350	GO:0016254
5351	GO:0016744
5352	GO:0006685
5353	GO:0003724
5354	GO:0030810
5355	GO:0004802
5356	GO:0004308
5357	GO:0004035
5358	GO:0004767
5359	GO:0008026
5360	GO:0042168
5361	GO:0033015
5362	GO:0030801
5363	GO:0033013
5364	GO:0016724
5365	GO:0051567
5366	GO:0006778
5367	GO:0030828
5368	GO:0047760
5369	GO:0051568

5370	GO:0006787
5371	GO:0034968
5372	GO:0004322
5373	GO:0042054
5374	GO:0030825
5375	GO:0016279
5376	GO:0016278
5377	GO:0070815
5378	GO:0030804
5379	GO:0008276
5380	GO:0030730
5381	GO:0008170
5382	GO:0042799
5383	GO:0004499
5384	GO:0045981
5385	GO:0006306
5386	GO:0008475
5387	GO:0018024
5388	GO:0016571
5389	GO:0006305
5390	GO:0000033
5391	GO:0006491
5392	GO:0006560
5393	GO:0006488
5394	GO:0006562
5395	GO:0006487
5396	GO:0006707
5397	GO:0006304
5398	GO:0006561
5399	GO:0015924
5400	GO:0000104
5401	GO:0006526
5402	GO:0015923
5403	GO:0004449
5404	GO:0031545
5405	GO:0009103
5406	GO:0004448
5407	GO:0042594
5408	GO:0016127
5409	GO:0019798
5410	GO:0008653
5411	GO:0006102
5412	GO:0050905
5413	GO:0016813
5414	GO:0004579
5415	GO:0004111
5416	GO:0004576

5417	GO:0045240
5418	GO:0004656
5419	GO:0004571
5420	GO:0006552
5421	GO:0004735
5422	GO:0008250
5423	GO:0005947
5424	GO:0045986
5425	GO:0018196
5426	GO:0048385
5427	GO:0018279
5428	GO:0034653
5429	GO:0048387
5430	GO:0090075
5431	GO:0006564
5432	GO:0031526
5433	GO:0000015
5434	GO:0060087
5435	GO:0016774
5436	GO:0008206
5437	GO:0046034
5438	GO:0004619
5439	polarity_distribution_H.0.75_mean
5440	GO:0045261
5441	GO:0004082
5442	GO:0044455
5443	GO:0004083
5444	GO:0034220
5445	GO:0004634
5446	GO:0045259
5447	GO:0055081
5448	GO:0045263
5449	GO:0033180
5450	GO:0033176
5451	GO:0046219
5452	GO:0033178
5453	GO:0009331
5454	GO:0042435
5455	GO:0033177
5456	GO:0034377
5457	GO:0004046
5458	GO:0033692
5459	GO:0033179
5460	GO:0003847
5461	GO:0042776
5462	GO:0009205
5463	GO:0065005

5464	GO:0009206
5465	GO:0009201
5466	GO:0009199
5467	GO:0009144
5468	GO:0008900
5469	GO:0015077
5470	GO:0008553
5471	GO:0009142
5472	GO:0015405
5473	GO:0015399
5474	GO:0000276
5475	GO:0015078
5476	GO:0000275
5477	GO:0009145
5478	GO:0022890
5479	GO:0022857
5480	GO:0022892
5481	GO:0043492
5482	GO:0022804
5483	GO:0019829
5484	GO:0006754
5485	GO:0042625
5486	GO:0042626
5487	GO:0006818
5488	GO:0046933
5489	GO:0016820
5490	GO:0046961
5491	GO:0016469
5492	GO:0016471
5493	GO:0006119
5494	GO:0015985
5495	GO:0005753
5496	GO:0015988
5497	GO:0015986
5498	GO:0015992
5499	GO:0015991
5500	GO:0005215
5501	GO:0015672
5502	GO:0001659
5503	GO:0032994
5504	GO:0034358
5505	GO:0034372
5506	GO:0034375
5507	GO:0006885
5508	GO:0034370
5509	GO:0043226
5510	GO:0034364

5511	GO:0043229
5512	GO:0021761
5513	GO:0009215
5514	GO:0009151
5515	GO:0047555
5516	GO:0004114
5517	GO:0003876
5518	GO:0004112
5519	GO:0030312
5520	GO:0046716
5521	GO:0030002
5522	GO:0006699
5523	GO:0051597
5524	GO:0033238
5525	GO:0042446
5526	GO:0035094
5527	GO:0034435
5528	GO:0034433
5529	GO:0008037
5530	GO:0034434
5531	GO:0000271
5532	GO:0006525
5533	GO:0055067
5534	GO:0007186
5535	GO:0012510
5536	GO:0004568
5537	GO:0004815
5538	secondary_structure_distribution_P.1.0_max
5539	GO:0006422
5540	GO:0007606
5541	GO:0003008
5542	GO:0050877
5543	GO:0050890
5544	GO:0035381
5545	GO:0003886
5546	GO:0006030
5547	GO:0004984
5548	GO:0009008
5549	GO:0006032
5550	GO:0046920
5551	GO:0004064
5552	GO:0004063
5553	GO:0007600
5554	GO:0004931
5555	GO:0007608
5556	polarizability_distribution_N.1.0_max
5557	GO:0030130

5558	GO:0004930
5559	GO:0004888
5560	VanDerWaal_distribution_N.1.0_max
5561	GO:0050954
5562	GO:0044462
5563	GO:0030313
5564	GO:0009058
5565	GO:0016787
5566	GO:0045932
5567	GO:0042311
5568	GO:0042417
5569	GO:0044249
5570	GO:0001540
